# Supplementary material for: Cross-sectional questionnaire study to gather the teaching preferences and expectations of UK undergraduate medical students for culinary medicine learning
Source: BMJ Open. 2020 Oct 14;10(10):e036410. doi: 10.1136/bmjopen-2019-036410 (PMC7559047; doi:10.1136/bmjopen-2019-036410)
Supplement: Author's manuscript [file bmjopen-2019-036410.draft_revisions.pdf]

BMJ Open

BMJ Open is committed to open peer review. As part of this commitment we make the peer review history of every article we publish publicly available.

When an article is published we post the peer reviewers' comments and the authors' responses online. We also post the versions of the paper that were used during peer review. These are the versions that the peer review comments apply to.

The versions of the paper that follow are the versions that were submitted during the peer review process. They are not the versions of record or the final published versions. They should not be cited or distributed as the published version of this manuscript.

BMJ Open is an open access journal and the full, final, typeset and author-corrected version of record of the manuscript is available on our site with no access controls, subscription charges or pay-per-view fees (http://bmjopen.bmj.com).

If you have any questions on BMJ Open's open peer review process please email info.bmjopen@bmj.com

BMJ Open

What Are the Preferences and Expectations of UK Undergraduate Medical Students From a Culinary Medicine Course?

Journal:	*BMJ Open*
Manuscript ID	bmjopen-2019-036410
Article Type:	Original research
Date Submitted by the Author:	16-Dec-2019
Complete List of Authors:	Xie, Jessica; University College London Research Department of Primary Care and Population Health,<br>Poduval, Shoba; University College London, Research Department of Primary Care & Population Health<br>Vickerstaff, Victoria; University College London, Division of Psychiatry; University College London, University College London Research Department of Primary Care and Population Health<br>Park, Sophie; University College London Research Department of Primary Care and Population Health
Keywords:	MEDICAL EDUCATION & TRAINING, NUTRITION & DIETETICS, EDUCATION & TRAINING (see Medical Education & Training)

SCHOLARONE™
Manuscripts

I, the Submitting Author has the right to grant and does grant on behalf of all authors of the Work (as defined in the below author licence), an exclusive licence and/or a non-exclusive licence for contributions from authors who are: i) UK Crown employees; ii) where BMJ has agreed a CC-BY licence shall apply, and/or iii) in accordance with the terms applicable for US Federal Government officers or employees acting as part of their official duties; on a worldwide, perpetual, irrevocable, royalty-free basis to BMJ Publishing Group Ltd ("BMJ") its licensees and where the relevant Journal is co-owned by BMJ to the co-owners of the Journal, to publish the Work in this journal and any other BMJ products and to exploit all rights, as set out in our licence.

The Submitting Author accepts and understands that any supply made under these terms is made by BMJ to the Submitting Author unless you are acting as an employee on behalf of your employer or a postgraduate student of an affiliated institution which is paying any applicable article publishing charge ("APC") for Open Access articles. Where the Submitting Author wishes to make the Work available on an Open Access basis (and intends to pay the relevant APC), the terms of reuse of such Open Access shall be governed by a Creative Commons licence – details of these licences and which Creative Commons licence will apply to this Work are set out in our licence referred to above.

Other than as permitted in any relevant BMJ Author's Self Archiving Policies, I confirm this Work has not been accepted for publication elsewhere, is not being considered for publication elsewhere and does not duplicate material already published. I confirm all authors consent to publication of this Work and authorise the granting of this licence.

For peer review only

What Are the Preferences and Expectations of UK Undergraduate Medical Students From a Culinary Medicine Course?

Author 1: Jessica Ying-Yi Xie BSc

Research Department of Primary Care and Population Health, Upper Third Floor, UCL Medical School, Rowland Hill Street, London, NW3 2PF, UK

jx9988_@hotmail.co.uk

Author 2: Dr Shoba Poduval

Research Department of Primary Care and Population Health, Upper Third Floor, UCL Medical School, Rowland Hill Street, London, NW3 2PF, UK

s.poduval@ucl.ac.uk

+44 (0)20 8016 8357

Author 3: Dr Victoria Vickerstaff

Division of Psychiatry, UCL, 6th Floor, Wing B, Maple House, 149 Tottenham Court Road London, W1T 7NF

Research Department of Primary Care and Population Health, Upper Third Floor, UCL Medical School, Rowland Hill Street, London, NW3 2PF, UK v.vickerstaff@ucl.ac.uk

v.vickerstaff@ucl.ac.uk

+44 (0)20 7679 9721

Author 4: Dr Sophie Park (Corresponding Author)

Research Department of Primary Care and Population Health, Upper Third Floor, UCL Medical School, Rowland Hill Street, London, NW3 2PF, UK

sophie.park@ucl.ac.uk

+44 (0)20 8016 8276

Total word count: 4040

Keywords: undergraduate medical education & training, quantitative research, culinary medicine, lifestyle medicine

3 tables, 4 figures

For peer review only

Abstract

Objective: To determine undergraduate medical students' learning needs and preferences in the area of Culinary Medicine (CM) with a view to informing development of a CM course at a UK medical school.

Setting: a single, urban UK medical school.

Participants: 180 undergraduate UK medical students.

Study Design: Cross-sectional questionnaire study collecting quantitative and qualitative (free-text) data.

Methods and Outcome Measures: An online questionnaire consisting of 16 questions of various styles (Likert-type, multiple choice and free-text). Quantitative analysis of multiple choice and Likert-type scale questions was conducted. Qualitative thematic analysis was used to analyse the free-text responses and identify themes.

Results: Three core themes related to students' understanding of CM were identified. (1) "CM Learning" – Students' perceived relevance of CM knowledge, perceived relevance of CM to healthcare, and their expectations for training. (2) "The Relationship between Food and Health" – and links between diet, social factors and health. (3) "Evidence-based Medicine" – and Student perceptions about scientific principles underlying CM. Quantitative analysis revealed that, although 83% of students felt that CM training is important for their future clinical practice, 56% felt unable to take a dietary history. 73% of students were dissatisfied with the quality, and 78% were dissatisfied with the quantity, of CM training provided by their medical school. Topics that students would like to be included in a CM course included weight management and portion control, and they felt that problem-based-styled learning would be the most appropriate method for delivering CM teaching.

Conclusions:

We gathered students' preferences for CM teaching, including course topics and delivery, which, if integrated into undergraduate medical training, could address students' learning needs. Further research in the area of CM in undergraduate medical education is required to confirm whether or not the findings of this study are transferrable to other medical schools.

For peer review only

Article Summary

Strengths and Limitations of this study

- The questionnaire was piloted to confirm that the questions fulfilled the study aim.
- All students enrolled at a UK medical school (academic year 2018-19) were invited to take part in this study to gather a wide breadth of views and experiences of CM.
- The study was conducted at one medical school only, limiting the transferability of the study findings to other medical schools.
- Although the target number of questionnaire responses (313) was not achieved, theoretical saturation was reached in the qualitative thematic analysis of the first 148 responses free-text responses, implying that sufficient qualitative data had been collected.
- JX and ShP independently analysed the free-text responses to gather different perspectives on the data.

Introduction

Culinary Medicine (CM) may be defined as "an evidence-based field in Medicine that blends the art of food and cooking with the science of Medicine".[1] A CM course can offer online learning, didactic teaching and practical hands-on culinary skills in a kitchen setting. The aim is to give students the knowledge and skills to help patients make informed decisions about accessing and eating healthy meals. The General Medical Council (GMC) expects UK medical school graduates to be competent in recognising ill health as a result of poor nutrition, and to be able to apply dietary knowledge to clinical practice.[2] However, it has been reported that medical students and doctors worldwide feel under-equipped to provide dietary advice for patients.[3–6] Studies have found that medical students who receive CM training are more likely to have a positive attitude towards, and better knowledge of, diet and nutrition; adopt better health habits themselves; and are more competent and confident in dietary counselling.[7–10] With input from experts in the field of CM, the Primary Care Medical Education Team at a UK medical school designed a CM course that was introduced into the Primary Care module of Year 5 of the undergraduate medical (MBBS) curriculum in September 2019. The aims of this CM course are to improve students' confidence in raising the topic of healthy eating, and giving simple dietary advice based on evidence-based recommendations; and to increase students' awareness about potential barriers to healthy eating (for example, cultural norms and low household income). This study was conducted to inform the development of the course by determining undergraduate medical students' learning needs and preferences in the area of CM.

Methods

We carried out a questionnaire study and collected quantitative and qualitative data using an online (Google Forms) questionnaire. JX (a fourth-year medical student and intern in the University College London (UCL) department of Primary Care and Population Health, carried out all data collection and led the analysis.

For peer review only

The study was conducted at a single, urban UK medical school from December 2018 to April 2019.

Our study population was 1669 undergraduate medical students enrolled in the academic year 2018-19 (data obtained from UCL Student Data Analyst).

Our target sample size was calculated using an online calculator.[11] We aimed to obtain 313 survey responses for our results to be statistically significant at the 95% confidence interval with a 5% margin for error.

The questionnaire was advertised to all medical students via posters, social media and in student societies' e-newsletters, as well as word of mouth.

Informed consent was obtained from study participants at the start of the questionnaire. Students were required to read the participant information sheet (PIS) on the first page of the questionnaire and to confirm that they fully understood their role in the study and how the data collected could be used. Once confirmed, they were able to proceed to the questionnaire on the next page.

Questionnaires were completed anonymously and no participant-identifiable data was collected. The only personal data collected was year of study.

There was a total of 16 questions of various styles (Likert-type, multiple choice and free-text) across four main topics (see Table 1).

Table 1: *The topic, description and type of questions included in the questionnaire.*

Topic	Question	Question type
(1) Education	We asked students for their understanding of CM and if they have any relevant previous experience of this style of teaching.	Free-text
(2) Integrating CM knowledge into clinical practice	We asked if students feel able to facilitate discussions about diet and provide dietary advice for patients.	Likert-type
(3) Provision of CM training	We asked how satisfied students are with the current standard of CM training delivered by their medical school.	Multiple choice and Likert-type
(4) Features of CM training	We asked about students' expectations of CM training for undergraduate medical students, including when they feel the training should be delivered.	Multiple choice

At the end of the questionnaire, we provided a free-text box for additional general comments and weblinks for those who wished to learn more about CM.

Patient and Public Involvement

Prior to data collection, two medical students (third-year and fifth-year) were asked to pilot the questionnaire. These students suggested ideas for improvement, including re-wording of questions to improve clarity. The questionnaire was subsequently updated accordingly.

Ethical Approval

Ethical approval to carry out this study was received from University College London Research Ethics Committee (ref.12471/002).

Analysis

Qualitative

Nvivo 12[(12)] was used to store free-text data in order to conduct a qualitative thematic analysis and identify core themes.[(13)] JX and ShP independently coded all free-text responses, and discussed their different interpretations of the data. Codes were derived deductively from the questionnaire and inductively upon analysis of the free-text responses.[(14)] Careful consideration was given upon coding of the data: new codes were compared against preliminary codes and refined, and the data was re-coded using the updated set of codes. This improved the quality of analysis.[(15)] Similar codes were categorized under a sub-theme or core theme. JX and ShP checked that each theme contained data relevant to only that theme. Theoretical saturation was identified after coding the first 148 responses in an interim analysis.

Quantitative

We carried out descriptive statistics to describe which year group the students were in. We presented responses to Likert-type and multiple-choice questions using proportions and frequencies, and compared responses from pre-clinical (years 1-3) and clinical (years 4-6) students using Chi-squared and Fisher's Exact tests where possible. Statistical significance level was set to 5%. SPSS Statistics 25[(16)] was used to conduct the quantitative analysis.

Results

STUDY POPULATION

We obtained an 11% response rate (180 responses). Fourth-year medical students were particularly interested in participating in this study, contributing to 25% of respondents (see Figure 1), as they were to be the first cohort to receive CM training.

STUDENTS' UNDERSTANDING OF CULINARY MEDICINE

There was a wide range of expectations for CM teaching. Three core themes were identified (see Table 2).

Table 2: *Themes and sub-themes identified from qualitative thematic analysis of responses to the free-text question 'What do you understand by the term 'CM'?'.*

	Core Theme	Sub-themes

For peer review only

1	**CM Learning**	Students' Perceived Relevance of CM Knowledge and Its Application for their Future Clinical Practice
		Students' Perceived Relevance of CM to Healthcare Professionalisms
		How to Approach Learning CM
2	**The Relationship Between Food and Health**	Diet and Physical and Mental Health
		Social Factors
3	**Evidence-based Medicine**	

CM Learning

Medical students expressed their understanding of CM in terms of what it might add to their training.

Students' Perceived Relevance of CM Knowledge and Its Application for their Future Clinical Practice

Many students believed that they should gain practical clinical knowledge and skills from the CM course, which can be applied to improving the health of patients.

> ***"'clinical' and applicable to a clinical session"*** (Third Year).

> ***"how to elicit behaviour change"*** (Second Year).

Learning how to facilitate discussions about diet with patients arose as a key topic.

> ***"sensitively debunk health fads"*** (Fifth Year).

> ***"how to conduct a consultation without body shaming"*** (Fifth Year).

Many students stated that they would like to be taught how to recognise different eating habits and patterns in order to better understand patients' diets, increase doctor-patient rapport and better facilitate future planning discussions.

> ***"understanding different people's feeding behaviours"*** (Second Year).

> ***"diet lifestyles**, such as **veganism"*** (Second Year).

A small minority of students had never heard of the term CM or were uncertain about its definition. This suggests a gap in their knowledge and an area for improvement in the MBBS curriculum.

> "***first time I came across this term***" (Fifth Year)

Most students were for the idea of the introduction of CM into the MBBS curriculum and believed that learning CM is important and relevant to their future clinical practice.

> *"**diet is**… in the context of obesity, longevity and mental health, a **contemporary issue in UK medical practice**."* (Second Year).

A small minority of students felt that a CM course would not benefit them greatly. Some expressed worry that other topics in the current MBBS curriculum, which they feel are more valuable than CM, may be compromised or completely removed.

> *"**other parts of the curriculum… should be better developed and prioritised**."* (Third Year).

However, others considered how they think the knowledge and skills that they will gain from CM training will enable them to be more competent to provide more effective advice and thus allow them to provide better patient-centred care.

> "*we are **taught very little about [food]** and **may have to go on our own knowledge to give patients advice, which may be biased** or **misguided**.*" (Fifth Year).

Students' Perceived Relevance of CM to Healthcare Professional Practice

There were different ideas about how CM relates to medical specialities, and the role of someone trained in CM in the multidisciplinary healthcare team (MDT).

Most students thought that CM is related to, or encompasses, nutrition.

> *"**related to nutrition**"* (Fifth Year).

Students also associated CM with lifestyle medicine.

> *"part of the **wider discipline of 'lifestyle medicine'**"* (Third Year).

Some students felt that learning about food and nutrition is in the realm of a dietician and that CM should not be included on the MBBS curriculum.

> *"there are… **dieticians**. [Doctors] **refer** patients to them"* (Fifth Year).

A few students believed that qualified psychologists or other HCPs are, compared to doctors, in positions of greater clinical expertise to encourage patients to make positive changes to their diet.

> *"**allied health professionals**… **are better suited in coaching patients in dietary change,** such as **dieticians** and **psychologists**. I don't think that having doctors educate and coach patients on dietary change is efficient for the physician or for the system"* (Fifth Year).

How to Approach Learning CM

Students held a variety of opinions on how CM training could be delivered for undergraduate medical students.

Students who were enthusiastic about the idea of learning practical culinary skills believed that this teaching method would be engaging, and allow them to gain essential life skills that would enable them to make improvements to their own health and wellbeing.

> *"a practical kitchen/cooking session… would add **variety**"* (First Year).

> *"practical kitchen sessions would **provide great insight into how to prepare healthy meals for students as well as future doctors**. Students are known for eating very poor diets and it affects our physical & mental health too"* (Fourth Year).

However, other students expressed distaste for a practical kitchen session, believing that the session would be poorly taught and that the skills gained would not useful in clinical practice.

For peer review only

"a practical "kitchen session" could be very ***difficult to organise*** *and the* ***quality of it is likely to be poor"*** (Fifth Year).

"I ***don't really see how it is practical*** *to teach practical culinary skills… I* ***don't see what advantage this would confer*** *as* ***a) we aren't going to be cooking patient's food*** *and b)* ***we won't have time to show them how to cook a meal****…being able to* ***advise [patients] on heathy choices*** *and being able to give them* ***adequate resources*** *is more feasible"* (Fourth Year).

A few students felt that shadowing HCPs would be a valuable learning experience.

*"****shadow a dietician****"* (Fifth Year).

"sitting in with ***diabetic nurses*** *and going through some of the* ***dietary restrictions*** *[for] patients"* (Fifth Year).

Other students expressed preference for more traditional teaching methods.

"a ***formal tutorial*** *on the…* ***DAFNE*** *[Dose Adjustment For Normal Eating]* ***diet*** *would [be] even more useful"* (Fifth Year).

Students suggested how CM could be integrated into the current MBBS curriculum.

A few clinical students felt that CM teaching should be taught alongside motivational interviewing. This would enable students to practice exploring patients' ideas, concerns and expectations regarding diet and health, and to work together with the patient towards mutual goals for health and wellbeing improvement.

"lifestyle advice [teaching] could be ***combined with the patient centred pathway teaching*** *in year 5* ***on motivational interviewing****"* (Fifth Year).

Some students felt that it would be appropriate to dedicate one day in the MBBS curriculum to CM teaching, emphasising that teaching should not exceed this amount, as they felt that medical students are likely to already have a good understanding of what constitutes a healthy diet from teaching received elsewhere in the MBBS curriculum.

"it would make sense to have a ***culinary medicine day****…* ***any more than that would be excessive****.* ***Most students will already know the basics of constructing a good diet****"* (Fifth Year).

There were a range of opinions on which year of the MBBS curriculum CM training should be delivered in.

"integrate ***preclin[ical]*** *science into FNM ['Fluids, Nutrition and Metabolism' - a module taught in Year 1 of the MBBS curriculum], then teach clinically in* ***year 5****"* (Fourth Year).

"take this module as a ***final year*** *medical student… as an* ***optional extra module****"* (Fifth Year).

The Relationship Between Food and Health

Diet and Physical and Mental Health

Most students recognised a link between diet and physical and/or mental health.

"nutrition and its impact on a patient's ***physical*** *and* ***mental wellbeing****"* (Third Year).

Some students reported that diet plays a central role in preventing, treating and managing disease, and alleviating symptoms due to ill-health.

For peer review only

> *"helping your patients [to] maintain healthy and well-balanced diets in order to live healthier lives and **prevent many diseases**"* (Fourth Year).

A few students highlighted that it may be dangerous to place a strong focus on how dietary changes can induce weight loss, as this could negatively impact mental wellbeing.

> *"it is really important that… there is more than just a focus on **'healthy' diets and weight** loss, but also to focus on **eating disorders** including **anorexia**, **bulimia**, **binge eating disorder** and other **behavioural disorders**"* (Fourth Year).

These students suggested that it would be better to adopt a patient-centred approach, in order to recognise individual patients' relationship with food, and for HCPs to promote the health benefits of a balanced diet, rather than labelling foods as inherently good or bad.

> *"**dieting** has been proven to have numerous **negative effects on health**… [training] should **focus [on] the health benefits of good diet** [and] should not… label foods as 'good' or 'bad' as this leads to **moral judgement**, **restriction** and **reactive bingeing** on 'forbidden' foods"* (Fourth Year).

<u>Social Factors</u>

Students commented on factors that could influence diet and health, including ethnic and cultural background, socio-economic status and level of education.

> "***level of education**… affects your health*" (Fourth Year).

> "***tailor** [patients' diets] to **lifestyle**, **ethnic**, **economic/ social factors***" (Third Year).

Some students stressed the importance of HCPs identifying and addressing these factors to identify how to best work with patients to improve their diet and health.

> "*understanding… [patients'] **individual barriers to healthy eating***" (Second Year).

A few students believed that doctors should have an understanding of cultural variation in diets to be able to provide patient-centred dietary advice. This will improve patients' understanding of, and is likely to increase their adherence to, dietary advice.

> *"my mum **cooks traditional Indian food on a daily basis** and hence they **prefer seeing an Indian GP when they go to the doctors**"* (Third Year).

<u>**Evidence-Based Medicine**</u>

Many students felt that a key principal of CM is that it is evidence-based.

> *"**evidence-based practice** of influencing health through lifestyle changes in food consumption"* (First Year).

However, a few students expressed concern about the about the amount of poorly evidenced information about diet and nutrition that is publicly available.

> *"I'm… worried about the **quality** and the **evidence base** for [CM]. **Diet and nutrition are**… **affected by**… **low-quality research**"* (Fifth Year).

INTEGRATING CM KNOWLEDGE INTO CLINICAL PRACTICE

48% of pre-clinical and 25% clinical students felt unable to facilitate a discussion with patients about diet and/or dietary change ($p = 0.005$, see Table 3), suggesting that students

For peer review only - http://bmjopen.bmj.com/site/about/guidelines.xhtml

felt that they were incompetent and/or they lacked confidence in their communication skills, particularly in the pre-clinical years.

72% of pre-clinical and 43% clinical students felt unable to take a dietary history from a patient (p = 0.001, see Table 3). This indicates that students would value additional teaching on this, again, particularly in the pre-clinical years.

64% pre-clinical and 57% clinical students felt unable to apply principles, methods and knowledge relating to nutrition to medical practice and integrate these into patient care (p = 0.498**,** see Table 3). This suggests a gap in students' knowledge related to nutrition and an uncertainty of how to apply this knowledge to clinical practice to allow for patient-centred care.

7% pre-clinical and 10% clinical students felt able to provide dietary advice for patients with varying cultural, social and economic needs (p = 0.617**,** see Table 3). This reveals the need to increase students' awareness of the social factors specific to individual patients that influence diet and access to food, in order for enable students to provide patients with personalised dietary advice.

80% of pre-clinical and 86% of clinical students felt that CM training is important for their future clinical practice (p = 0.482, see Table 3). This implies that most students felt that an understanding of CM is relevant to, and required for, clinical practice.

Table 3: *Results from quantitative analysis of responses to the Likert-type questions. Results are presented as number (percentage).*

**Statement**		**Pre-Clinical (n=81)**	**Clinical (n=99)**	**Total (n = 180)**	**p-value**
**I am able to facilitate a discussion with patients about diet and/or dietary change.**	Disagree	39 (48.1)	25 (25.3)	64 (35.6)	**p = 0.005**
	Neutral	28 (34.6)	44 (44.4)	72 (40.0)	
	Agree	14 (17.3)	30 (30.3)	44 (24.4)	
**I am able to take dietary history from a patient.**	Disagree	59 (71.6)	43 (43.4)	101 (56.1)	**p = 0.001**
	Neutral	14 (17.3)	31 (31.3)	45 (25.0)	
	Agree	9 (11.1)	25 (25.3)	34 (18.9)	
**I am able to apply principles, methods and knowledge relating to nutrition to medical practice and integrate these into patient care.**	Disagree	52 (64.2)	56 (56.6)	108 (60.0)	**p = 0.498**
	Neutral	22 (27.2)	30 (30.3)	52 (28.9)	
	Agree	7 (8.6)	13 (13.1)	20 (11.1)	
**I am able to provide dietary advice for patients with varying cultural, social and economic needs.**	Disagree	57 (70.4)	63 (63.6)	120 (66.7)	**p = 0.617**
	Neutral	18 (22.2)	26 (26.3)	44 (24.4)	
	Agree	6 (7.4)	10 (10.1)	16 (8.9)	
**I believe that learning Culinary Medicine is important for my future clinical practice.**	Disagree	6 (7.4)	4 (4.0)	10 (5.6)	**p = 0.482**
	Neutral	10 (12.3)	10 (10.1)	20 (11.1)	

For peer review only

	Agree	65 (80.2)	85 (85.9)	150 (83.3)	

PROVISION OF CM TRAINING

62% of students reported having received no prior CM training (see Figure 2).

68% pre-clinical and 77% clinical students were dissatisfied with the quality of CM training provided by the medical school. 72% of pre-clinical and 84% of clinical students were dissatisfied with the current quantity of CM training provided by the medical school (see Figure 3).

FEATURES OF CM TRAINING

Students were asked to suggest features of an ideal CM course, including course content and delivery. Students felt that it is most appropriate for a CM course to include teaching on weight management and portion control (157 responses), followed by the types of diet and their evidence base (156 responses). Nutrition psychology was the least popular topic (128 responses, see Figure 4).

Students felt that the most appropriate methods of providing CM training are problem-based learning (140 responses) and small-group seminars/ tutorials (138 responses, see Figure 2).

67% of students expressed a preference for CM training to be delivered in pre-clinical training (pre-clinical students 72%, clinical students 64%, p = 0.257).

Discussion

This is the first questionnaire study that has explored UK medical students' learning needs and preferences in the area of CM.

Our study findings revealed a variety of students' understandings and attitudes towards CM training. Some students positioned CM as highly relevant to patient care. These findings are consistent with a study carried out at an American University[8], where students believed that CM training would improve their ability to facilitate dietary counselling, and that they would be able to apply the knowledge and skills from the training to improve patients' overall health.

Some students perceived CM as relevant to clinical years' training, whereas others felt this was a pre-clinical topic and not directly related to clinical practice with patients. A few students believed that CM was irrelevant to medical student training and anticipated that the task of dietary counselling would be delegated to other HCPs, such as dieticians. These findings are inconsistent with literature that suggests the health benefits of dietary advice given by doctors, especially in general practice. The CM course is currently being delivered in the year 5 general practice module.[17–19]

Despite this, most students felt that CM training is important for their future clinical practice, although many did not feel confident in their knowledge, nor competent to provide specific dietary advice. These findings are similar to that of an Australian study[5], in which the majority of preclinical medical students felt that it was important for doctors to have an

For peer review only

understanding of nutrition in relation to conditions such as coeliac disease, but fewer than half were confident in applying their knowledge to clinical practice.

Furthermore, many students in our study believed that practical kitchen-based sessions would be an effective method of training. Their belief is consistent with a study[8] that found that medical students' confidence, nutrition knowledge and food identification skills improved after completing a CM course with hands-on culinary skills training.

Our findings suggest that an key components of a CM training course are: explaining the clinical and professional relevance of this learning, and supporting medical students in developing an understanding of how this knowledge might be applied in clinical practice to maximise opportunities for supporting health promotion and managing ill health.

Strengths and Limitations

An important potential limitation to consider is that CM is a relatively new term in medicine and is not widely used amongst HCPs in the UK. This is due to CM training not yet being included in the curriculum of the majority of UK medical schools. The authors anticipated that only students who were familiar with the term CM were likely to complete the questionnaire. Therefore, in order to encourage all students to participate, advertisements for the questionnaire, as well as the PIS on the first page of the questionnaire, emphasised that students do not need to have any understanding of CM or lifestyle medicine or nutrition in order to take part in this study.

Prior to data collection, the questionnaire was piloted by two current undergraduate medical students to confirm that the questions fulfill the study aim. This validation step increased the reliability of the questionnaire.

The questionnaire responses were received from one UK medical school only. This limits the transferability of the study findings to other medical schools. However, these findings may be useful in informing the development of future CM courses for undergraduate medical students elsewhere.

Also, the sample size was small (180), and the target number of questionnaire responses (313) was not achieved. However, theoretical saturation was reached after coding the first 148 responses in an interim analysis, implying that sufficient data has been obtained and strengthening the study's credibility.

Furthermore, thematic analysis of free-text responses was carried out independently by both JX and ShP. This allowed a range of interpretations on the data to be gathered.

However, most of the probability (p) values obtained for the quantitative data were greater than 0.05, implying that results were not significant at the 95% confidence interval and there may have been a greater than 5% probability that results were due to chance.

Implications

The findings from this study will be used to inform the development of a CM course for fifth-year undergraduate students at a UK medical school during their training in Primary Care.

For peer review only

A number of recommendations can be made. A small number of our study participants had never come across the term CM. This suggests that there is a need for the CM course to define the key principles and aims of CM.

Students are currently taught CM through case-based discussions, practical culinary skills training and online learning. Our findings suggest that students would like to be taught using additional teaching methods, such as problem-based learning and small group-seminars/ tutorials. These could be trialled in the future.

The CM course is currently integrated into the penultimate year of the MBBS curriculum to allow students to draw explicit links between patients, whom they encounter in general practice, and CM knowledge. Students also expressed a desire for pre-clinical training, with the option of enrolling on a student-selected module in CM in clinical training. The new CM course at this medical school could be evaluated in the future to assess whether students' learning needs have been sufficiently addressed, whether the course achieves its aims, and whether CM training could be integrated into multiple areas of the MBBS curriculum in the future.

Furthermore, this questionnaire study could be repeated in the future using a larger sample size from different UK medical schools to explore how medical students feel about CM training across the UK.

Conclusions

Most students believed that CM training is important for their future clinical practice. However, many felt unprepared to take a dietary history and give appropriate advice. The majority of students were dissatisfied with the quality and quantity of CM training provided by the medical school. Students expressed a preference for formal small group teaching about different types of diet and their evidence-base, and for teaching to be delivered at multiple stages during their undergraduate medical training. Future research in the area of CM in undergraduate medical education is required to confirm whether or not the findings of this study are transferrable to other medical schools.

Acknowledgements

We would like to thank Dr Ann Griffin (Director of the Research Department of Medical Education (RDME) of UCL Medical School), for granting us permission to recruit study participants from the UCL medical student cohort of 2018-19, and Professor Elizabeth Murray (Head of the Research Department of Primary Care and Population Health at UCL), for approving the study. We are thankful to the National Institute for Health Research School for Primary Care Research (NIHR SPCR) for providing a grant to fund this research project. Thank you to UCL Student Data Analyst for providing us with the number of medical students enrolled at UCL in the academic year 2018-19. Gratitude must also be expressed to Miss Valeriya Kopanitsa (stakeholder) and Mr Deepash Hosadurg (stakeholder) for reviewing the preliminary questionnaire. Finally, we are thankful to all the study participants who took the time to complete the questionnaire and share their invaluable thoughts on CM training.

For peer review only

Author Statement

JX was the main author who designed the study, collected and analysed the data and was the main author for the write-up. SP (fourth author) was the chief investigator. ShP (second author) was the project supervisor. Both SP and ShP helped design the study. ShP also contributed to participant recruitment and data analysis. VV supervised the statistical analysis. All authors provided input in the write-up and have approved the final manuscript.

Funding

This work was supported by the National Institute for Health Research School for Primary Care Research (NIHR SPCR) grant number 156780. VV receives seedcorn funding from the NIHR SPCR.

Competing Interests

None declared.

Patient Consent for Publication

Not required.

Ethics Approval

University College London Ethics (ref.12471/002).

Provenance and Peer Review

This paper was not peer reviewed prior to submission.

Data Availability Statement

The data generated from this study is not suitable for sharing beyond that contained within the report. Further information can be obtained from the corresponding author.

References

1. La Puma J. What Is Culinary Medicine and What Does It Do? Popul Health Manag. 2016 Feb;19(1):1–3.
2. General Medical Council. Outcomes For Graduates 2018. 2018; Available from: https://www.gmc-uk.org/-/media/documents/dc11326-outcomes-for-graduates-2018_pdf-75040796.pdf
3. Campbell D. Doctors 'know too little about nutrition and exercise' [Internet]. The Guardian; 2016 [cited 2019 Feb 17]. Available from: https://www.theguardian.com/society/2016/oct/19/doctors-know-too-little-about-effects-of-nutrition-and-exercise
4. Crowley J, Ball L, Hiddink GJ. Nutrition in medical education: a systematic review. Lancet Planet Heal. 2019 Sep;3(9):e379–89.
5. Perlstein R, McCoombe S, Shaw C, Nowson C. Medical students' perceptions regarding the importance of nutritional knowledge and their confidence in providing competent nutrition practice. Public Health [Internet]. 2016;140:27–34. Available from: http://www.sciencedirect.com/science/article/pii/S0033350616302207
6. Telegraph Reporters. GP training is outdated and needs to teach young doctors about

For peer review only

nutrition, experts claim [Internet]. Telegraph. 2018 [cited 2019 Feb 17]. Available from: https://www.telegraph.co.uk/news/2018/03/25/gp-training-outdated-needs-teach-young-doctors-nutrition-experts

7. Conroy MB, Delichatsios HK, Hafler JP, Rigotti NA. Impact of a preventive medicine and nutrition curriculum for medical students. Am J Prev Med. 2004 Jul;27(1):77–80.

8. Pang B, Memel Z, Diamant C, Clarke E, Chou S, Gregory H. Culinary medicine and community partnership: hands-on culinary skills training to empower medical students to provide patient-centered nutrition education. Med Educ Online. 2019 Dec;24(1):1630238.

9. Ring M, Cheung E, Mahadevan R, Folkens S, Edens N. Cooking Up Health: A Novel Culinary Medicine and Service Learning Elective for Health Professional Students. J Altern Complement Med. 2019 Jan;25(1):61–72.

10. Schlair S, Hanley K, Gillespie C, Disney L, Kalet A, Darby PC, et al. How Medical Students' Behaviors and Attitudes affect the Impact of a Brief Curriculum on Nutrition Counseling. J Nutr Educ Behav [Internet]. 2012;44(6):653–7. Available from: http://www.sciencedirect.com/science/article/pii/S149940461100529X

11. SurveyMonkey. Sample Size Calculator [Internet]. [cited 2019 Feb 17]. Available from: https://www.surveymonkey.co.uk/mp/sample-size-calculator/

12. NVivo qualitative data analysis Software, Version 12. QSR International Pty Ltd; 2018.

13. Braun V, Clarke V. Using thematic analysis in psychology. Qual Res Psychol [Internet]. 2006 Jan 1;3(2):77–101. Available from: https://www.tandfonline.com/doi/abs/10.1191/1478088706qp063oa

14. Stuckey H. The second step in data analysis: Coding qualitative research data . J Soc Heal Diabetes [Internet]. 2015 Jan 1;3(1):7–10. Available from: http://www.joshd.net/article.asp?issn=2321-0656

15. Hewitt-Taylor J. Use of constant comparative analysis in qualitative research. Nurs Stand. 2001 Jul;15(42):39–42.

16. IBM Corp. IBM SPSS Statistics for Macintosh, Version 25.0. Armonk, NY: IBM Corp; 2017.

17. Willaing I, Ladelund S, Jørgensen T, Simonsen T, Nielsen LM. Nutritional counselling in primary health care: a randomized comparison of an intervention by general practitioner or dietician. Eur J Cardiovasc Prev Rehabil [Internet]. 2004;11(6):513–20. Available from: https://doi.org/10.1097/01.hjr.0000152244.58950.5f

18. Pritchard DA, Hyndman J, Taba F. Nutritional counselling in general practice: a cost effective analysis. J Epidemiol Community Health [Internet]. 1999 May;53(5):311–6. Available from: https://www.ncbi.nlm.nih.gov/pubmed/10396539

19. Sacerdote C, Fiorini L, Rosato R, Audenino M, Valpreda M, Vineis P. Randomized controlled trial: effect of nutritional counselling in general practice. Int J Epidemiol [Internet]. 2005;35(2):409–15. Available from: https://doi.org/10.1093/ije/dyi170

For peer review only

Figure 1: *Results from quantitative analysis of responses to the single choice question 'Which year of the medical (MBBS) programme are you currently in?'.*

Figure 2: *A comparison of the results from quantitative analysis of responses to two multiple choice questions: 'What types of CM training have you received so far in the MBBS curriculum?' and 'Select the 3 most appropriate teaching methods for a CM course'.*

Figure 3: *Results from quantitative analysis of responses to the Likert-type questions: 'To what extent do you agree with the statements: "I am satisfied with the QUALITY of CM training that my medical school provides"? and 'To what extent do you agree with the statement "I am satisfied with the QUANTITIY of CM training that my medical school provides"?'.*

Figure 4: *Results from quantitative analysis of responses to the multiple choice question 'Which topics and/or skills do you think should be taught on the CM course?'.*

For peer review only

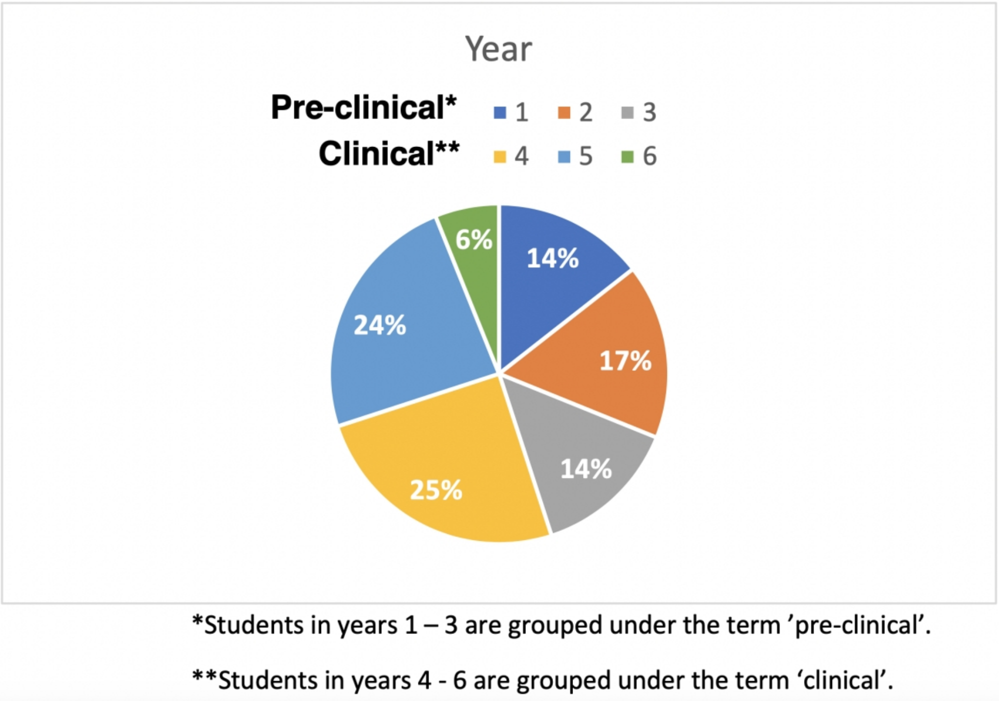

Figure 1: Results from quantitative analysis of responses to the single choice question 'Which year of the medical (MBBS) programme are you currently in?'.

351x245mm (144 x 144 DPI)

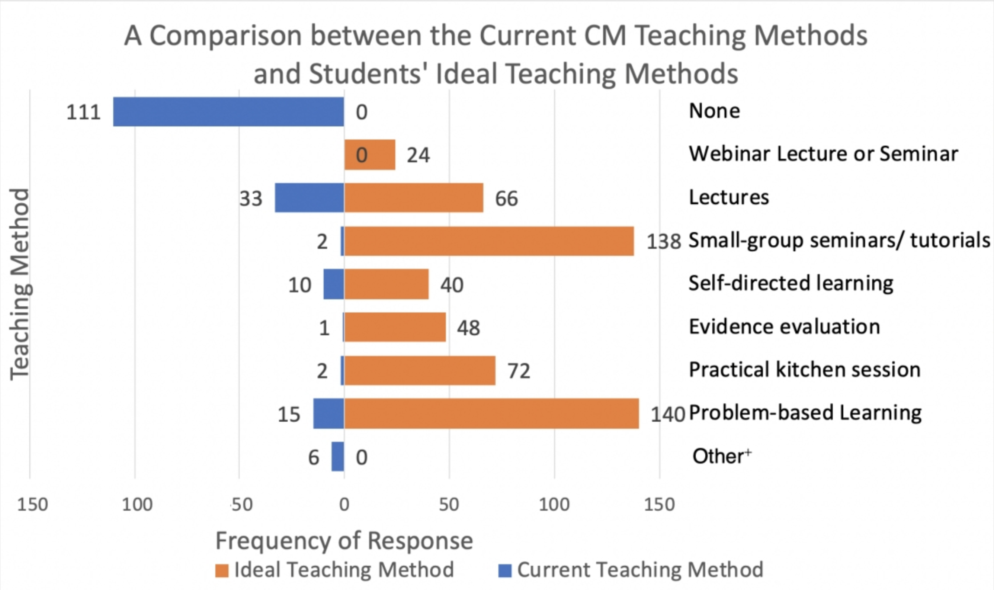

+ = Other includes "teaching on nutrition and vitamin deficiencies in relation to gastroenterology"; "clinical experience in GP"; "bedside teaching and clinics"; "general dietary tips"; "I can't remember"; and "not sure as I'm not sure what culinary medicine includes".

Figure 2: A comparison of the results from quantitative analysis of responses to two multiple choice questions: 'What types of CM training have you received so far in the MBBS curriculum?' and 'Select the 3 most appropriate teaching methods for a CM course'.

348x237mm (144 x 144 DPI)

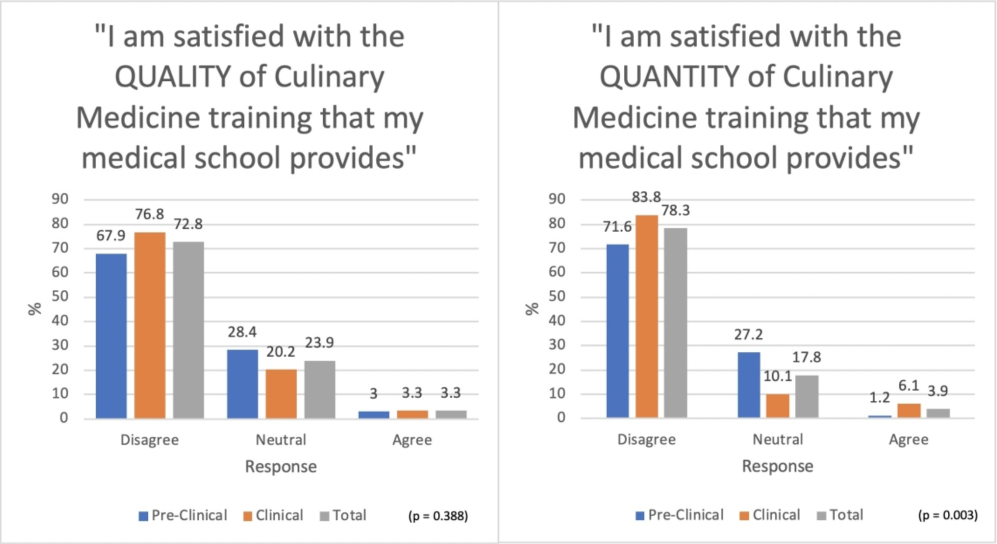

Figure 3: Results from quantitative analysis of responses to the Likert-type questions: 'To what extent do you agree with the statements: "I am satisfied with the QUALITY of CM training that my medical school provides"? and 'To what extent do you agree with the statement "I am satisfied with the QUANTITIY of CM training that my medical school provides"?'.

410x225mm (144 x 144 DPI)

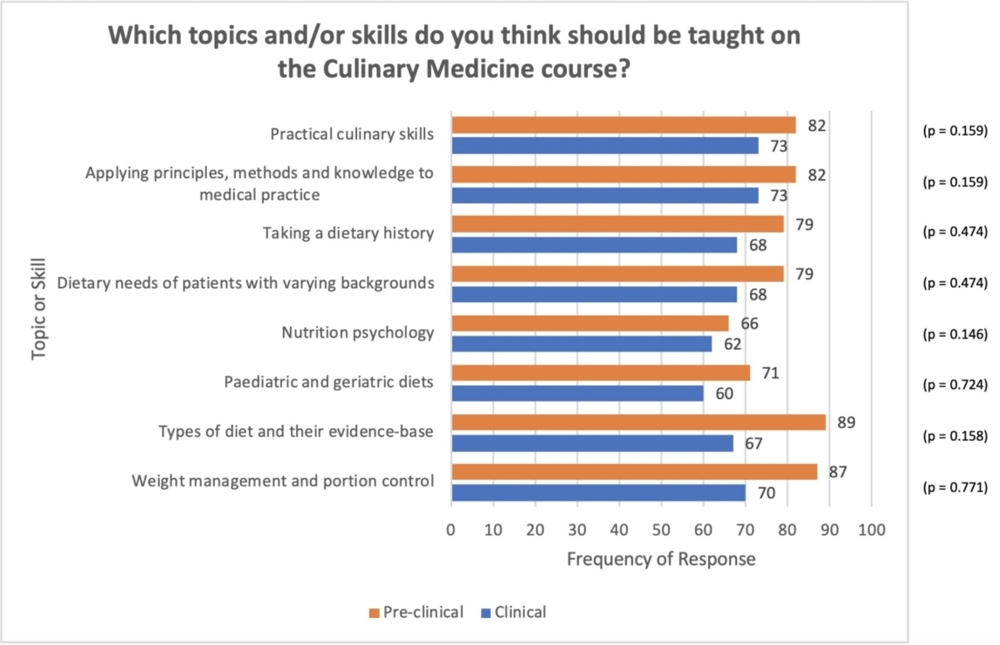

Figure 4: Results from quantitative analysis of responses to the multiple choice question 'Which topics and/or skills do you think should be taught on the CM course?'.

377x244mm (144 x 144 DPI)

A Cross-sectional Questionnaire Study to Gather the Teaching Preferences and Expectations of UK Undergraduate Medical Students For Culinary Medicine Learning

Journal:	*BMJ Open*
Manuscript ID	bmjopen-2019-036410.R1
Article Type:	Original research
Date Submitted by the Author:	03-Jun-2020

Complete List of Authors:	Xie, Jessica; University College London Research Department of Primary Care and Population Health,<br>Poduval, Shoba; University College London, Research Department of Primary Care & Population Health<br>Vickerstaff, Victoria; University College London, Division of Psychiatry; University College London, University College London Research Department of Primary Care and Population Health<br>Park, Sophie; University College London Research Department of Primary Care and Population Health

**Primary Subject Heading**:	Medical education and training
Secondary Subject Heading:	Nutrition and metabolism, General practice / Family practice, Medical education and training
Keywords:	MEDICAL EDUCATION & TRAINING, NUTRITION & DIETETICS, EDUCATION & TRAINING (see Medical Education & Training), PRIMARY CARE

SCHOLARONE™ Manuscripts

I, the Submitting Author has the right to grant and does grant on behalf of all authors of the Work (as defined in the below author licence), an exclusive licence and/or a non-exclusive licence for contributions from authors who are: i) UK Crown employees; ii) where BMJ has agreed a CC-BY licence shall apply, and/or iii) in accordance with the terms applicable for US Federal Government officers or employees acting as part of their official duties; on a worldwide, perpetual, irrevocable, royalty-free basis to BMJ Publishing Group Ltd ("BMJ") its licensees and where the relevant Journal is co-owned by BMJ to the co-owners of the Journal, to publish the Work in this journal and any other BMJ products and to exploit all rights, as set out in our licence.

The Submitting Author accepts and understands that any supply made under these terms is made by BMJ to the Submitting Author unless you are acting as an employee on behalf of your employer or a postgraduate student of an affiliated institution which is paying any applicable article publishing charge ("APC") for Open Access articles. Where the Submitting Author wishes to make the Work available on an Open Access basis (and intends to pay the relevant APC), the terms of reuse of such Open Access shall be governed by a Creative Commons licence – details of these licences and which Creative Commons licence will apply to this Work are set out in our licence referred to above.

Other than as permitted in any relevant BMJ Author's Self Archiving Policies, I confirm this Work has not been accepted for publication elsewhere, is not being considered for publication elsewhere and does not duplicate material already published. I confirm all authors consent to publication of this Work and authorise the granting of this licence.

For peer review only

A Cross-sectional Questionnaire Study to Gather the Teaching Preferences and Expectations of UK Undergraduate Medical Students For Culinary Medicine Learning

For peer review only

Author 1: Miss Jessica Ying-Yi Xie

Research Department of Primary Care and Population Health, Upper Third Floor, UCL Medical School, Rowland Hill Street, London, NW3 2PF, UK

jx9988_@hotmail.co.uk

Author 2: Dr Shoba Poduval

Research Department of Primary Care and Population Health, Upper Third Floor, UCL Medical School, Rowland Hill Street, London, NW3 2PF, UK

s.poduval@ucl.ac.uk

+44 (0)20 8016 8357

Author 3: Dr Victoria Vickerstaff

Division of Psychiatry, UCL, 6th Floor, Wing B, Maple House, 149 Tottenham Court Road London, W1T 7NF

Research Department of Primary Care and Population Health, Upper Third Floor, UCL Medical School, Rowland Hill Street, London, NW3 2PF, UK
v.vickerstaff@ucl.ac.uk

+44 (0)20 7679 9721

Author 4: Dr Sophie Park (Corresponding Author)

Research Department of Primary Care and Population Health, Upper Third Floor, UCL Medical School, Rowland Hill Street, London, NW3 2PF, UK

sophie.park@ucl.ac.uk

+44 (0)20 8016 8276

Total word count: 4135

Keywords: undergraduate medical education, quantitative research, questionnaire, culinary medicine, general practice, primary care

3 tables, 4 figures

Abstract

Aim: To determine undergraduate medical students' teaching preferences and expectations for Culinary Medicine (CM) learning with a view to informing development of a CM course at a UK medical school.

Setting: a single, urban UK medical school.

Participants: 180 undergraduate medical students.

Study Design: Cross-sectional questionnaire study collecting quantitative and qualitative (free-text) data.

Methods and Outcome Measures: An online questionnaire consisting of 16 questions of various styles (Likert-type, multiple choice and free-text). Quantitative analysis of multiple choice and Likert-type scale questions was conducted. Qualitative thematic analysis was used to analyse the free-text responses and identify themes.

Results: Three core themes related to students' understanding of CM were identified. (1) "CM Learning": students' perceived relevance of CM knowledge, perceived relevance of CM to healthcare, and their expectations for teaching. (2) "The Relationship between Food and Health": and links between diet, social factors and health. (3) "Evidence-based Medicine": and students' perceptions about scientific principles underlying CM. Quantitative analysis revealed that, although 83% of students felt that learning CM is important for their future clinical practice, 56% felt unable to take a dietary history. 73% of students were dissatisfied with the quality, and 78% were dissatisfied with the quantity, of CM teaching understood to be relevant to CM provided by their medical school. Topics that students would like to be included in a CM course included weight management and portion control. Students felt that problem-based-styled learning would be the most appropriate method for delivering CM teaching.

Conclusions:

This study revealed that medical students felt that their dietary counselling skills could be improved with further clinically-relevant teaching in the undergraduate medical curriculum. Students' preferences for CM learning have been taken into consideration in the development of a CM course for fifth-year undergraduate students at a UK medical school during their General Practice placements.

For peer review only

Article Summary

Strengths and Limitations of This Study

- The questionnaire was piloted to confirm readability and ensure questions fulfilled the study aim.
- At interim analysis of the first 148 questionnaire responses, no new codes were generated during qualitative thematic analysis of free-text responses, signifying that theoretical saturation had been reached.
- JX and ShP analysed the free-text responses independently to gather a wide range of interpretations of the data.
- The study was conducted at one medical school only, limiting the transferability of the study findings to other medical schools.
- The response rate (11%) was low which limits the generalisability of the results to the student population.

Introduction

Culinary Medicine (CM) may be defined as "an evidence-based field in Medicine that blends the art of food and cooking with the science of Medicine".[1] A CM course can offer online learning, didactic teaching and practical hands-on culinary skills in a kitchen setting. The aim is to give students the knowledge and skills to help patients make informed decisions about accessing and eating healthy meals. The General Medical Council (GMC) expects UK medical school graduates to be competent in recognising ill health as a result of poor nutrition, and to be able to apply dietary knowledge to clinical practice.[2] However, it has been reported that medical students and doctors worldwide feel under-equipped to provide dietary advice for patients.[3–6] Studies have found that medical students who learn CM are likely to develop positive attitude towards, and better knowledge of, diet and nutrition; adopt better health habits themselves; and become more competent and confident in dietary counselling.[7–10]

The setting for this study was a single, urban UK medical school, with a five-year undergraduate medical (MBBS) curriculum plus an Integrated Bachelor of Science (iBSc) in third year. In their first year, students receive lectures on nutritional science, such as the digestion of macronutrients and gastrointestinal physiology, and public health nutrition. These lectures are delivered in the pre-clinical part of the MBBS curriculum.

The Primary Care Medical Education Team at this UK medical school collaborated with a team of doctors, dietitians and chefs from Culinary Medicine UK[11] to create a CM course that was introduced into the Primary Care module of the year five MBBS curriculum in September 2019. Set in a teaching kitchen, the one-day course teaches students culinary skills, evidence-based medicine related to dietary counselling, and motivational interviewing skills. Believed to be the first medical school in Europe to provide mandatory CM teaching, the aims of the course are to improve students' confidence in raising the topic of healthy eating and giving simple dietary advice based on evidence-based recommendations; and to increase students' awareness about potential barriers to healthy eating (for example, cultural norms and low household income). This study was conducted prior to the introduction of the

For peer review only

Topic	Information to Gather	Question type

course to inform its development by determining students’ teaching preferences and expectations for CM learning.

Methods

This was a cross-sectional questionnaire study, collecting quantitative and qualitative data using an online (Google Forms) questionnaire. JX (a fourth-year medical student and School for Primary Care Research (SPCR) intern in the University College London (UCL) department of Primary Care and Population Health), carried out all data collection and led the analysis.

The study was conducted at a single, urban UK medical school from December 2018 to April 2019.

The study population was 1669 undergraduate medical students enrolled in the academic year 2018/19 (data supplied by UCL Student Data Analyst).

The questionnaire was advertised on posters and via social media and student societies' e-newsletters and word of mouth.

Informed consent was obtained from study participants at the start of the questionnaire. Students were required to read the participant information sheet (PIS) on the first page of the questionnaire and to confirm that they fully understood their role in the study and how the data collected would be used. Once confirmed, they were able to proceed to the questionnaire on the next page.

Questionnaires were completed anonymously, and no participant-identifiable data was collected. The only personal data collected was year of study.

There was a total of 16 questions of various styles (Likert-type, multiple choice and free-text) across four main topics (see Table 1). The first topic included establishing students’ understanding of the term ‘CM’. A free-text response was requested to explore the different ways students understood and interpreted the concept.

Table 1: *The topic, description and type of questions included in the questionnaire.*

For peer review only

(1) Education	Students' understanding of CM<br><br>Previous experience(s) of teaching understood to be relevant to CM	Free-text
(2) Integrating CM knowledge into clinical practice	Students' self-perceived abilities to facilitate discussions about diet and provide dietary advice for patients	Likert-type
(3) Teaching CM	Students' satisfaction with the current standard of teaching understood to be relevant to CM, delivered by their medical school	Multiple choice and Likert-type
(4) Features of a CM course	Students' expectations for a CM course for undergraduate medical students, including methods of delivering teaching and teaching topics	Multiple choice

At the end of the questionnaire, we provided a free-text box for additional general comments and weblinks for those students wished to learn more about CM.

Patient and Public Involvement

Prior to data collection, two medical students (iBSc third-year and fifth-year) were invited to pilot the questionnaire and comment on the readability. These students suggested re-wording of a few questions to improve clarity. The questionnaire was updated accordingly. Students were not invited to contribute to the study design, analysis or writing of this manuscript.

Ethical Approval

Ethical approval to carry out this study was received from UCL Research Ethics Committee (ref.12471/002).

Analysis

Qualitative

NVivo 12[12] was used to store free-text data in order to conduct a qualitative thematic analysis and identify core themes.[13] JX and ShP independently coded all free-text responses before sharing their interpretations with the qualitative results analysis team (JX, ShP and SP) to discuss any areas of dissonance or contrasting interpretation. Codes were derived deductively from the questionnaire (for example, 'Understanding of CM'), and inductively (for example, 'Social factors contributing to diet') upon analysis of the free-text responses.[14] Careful consideration was given upon coding of the data: new codes were compared against preliminary codes and refined, and the data was re-coded using the updated set of codes. This improved the quality of analysis.[15] Similar codes were categorized under a sub-theme or core theme. No new codes emerged after coding responses from the first 148 students in an interim analysis, suggesting that theoretical saturation had been reached.

For peer review only

Quantitative

SPSS Statistics 25[16] was used to conduct the quantitative analysis. The quantitative results analysis team (JX and VV) carried out descriptive statistics to describe which year group the students were in. Responses to Likert-type and multiple-choice questions were presented using proportions and frequencies, and responses from pre-clinical (years 1-3) and clinical (years 4-6) students were compared using Chi-squared and Fisher's Exact tests where possible. Statistical significance level was set to 5%.

Results

STUDY POPULATION

An 11% response rate (180 responses) was achieved. Fourth-year medical students were the largest contributors to the study (25% of respondents, see Figure 1). This may be because they were to be the first cohort to study the CM course.

STUDENTS' UNDERSTANDING OF CM

There was a wide range of expectations for CM teaching. Three core themes were identified (see Table 2).

Table 2: *Themes and sub-themes identified from qualitative thematic analysis of responses to the free-text question 'What do you understand by the term 'CM'?'.*

Core Theme		Sub-themes	
1	**CM Learning**	1	Students' Perceived Relevance of CM Knowledge and Its Application for their Future Clinical Practice
		2	Students' Perceived Relevance of CM to Healthcare Professionalisms
		3	How to Approach Learning CM
2	**The Relationship Between Food and Health**	1	Diet and Physical and Mental Health
		2	Social Factors
3	**Evidence-based Medicine**		

Core Theme 1: CM Learning

Medical students shared their understanding of CM, suggesting where CM training might fit their medical training.

Sub-theme 1: Students' Perceived Relevance of CM Knowledge and Its Application for their Future Clinical Practice

Students shared their expectations to gain clinical knowledge and practical skills from the CM course.

> *"**'clinical' and applicable to a clinical session**"* (Third Year).

Learning how to facilitate discussions about diet with patients arose as a key topic.

For peer review only

*"**sensitively debunk health fads**"* (Fifth Year).

*"**how to conduct a consultation without body shaming**"* (Fifth Year).

Students stated that they would like to be taught how to recognise different eating habits and patterns in order to better understand patients' diets, increase doctor-patient rapport and better facilitate future planning discussions.

*"**understanding different people's feeding behaviours**"* (Second Year).

***"diet lifestyles**, such as **veganism**"* (Second Year).

Other students were unfamiliar with the term CM.

"***first time I came across this term***" (Fifth Year).

Students who were in favour of a CM course stressed the importance of the topic and considered how the knowledge and skills that they will learn from the course will make them more competent to educate patients about diet patients and thus improve patients' health outcomes.

"*this **valuable knowledge**… is **applicable to every specialty***" (Fifth Year).

"*CM involves using nutrition to **improve patient's health**, including **teaching patients how to eat and cook healthily***" (Fourth Year).

However, some students felt that learning CM would not benefit them greatly. Students expressed worry that other topics in the current MBBS curriculum, which they felt were more useful than CM, might be compromised or completely removed.

*"**other parts of the curriculum… should be better developed and prioritised**"* (Third Year).

Sub-theme 2: Students' Perceived Relevance of CM to Healthcare Professional Practice

A variety of perspectives on how CM relates to medical specialities arose, as well as the role of someone trained in CM in the multidisciplinary healthcare team (MDT).

Students associated CM with nutrition and lifestyle medicine.

*"related to **nutrition**"* (Fifth Year).

*"part of the wider discipline of **'lifestyle medicine'**"* (Third Year).

Some students suggested that Healthcare Professionals (HCPs) with greater clinical expertise in this area compared to doctors would be make better dietary counsellors.

*"**allied health professionals**… **are better suited in coaching patients in dietary change,** such as **dietitians** and **psychologists**. I don't think that having doctors educate and coach patients on dietary change is efficient for the physician or for the system"* (Fifth Year).

Sub-theme 3: How to Approach Learning CM

Students offered a wide variety of opinions on the most appropriate methods for teaching CM in the MBBS curriculum. Students who were enthusiastic about the idea of learning culinary skills believed that practical learning would not only be engaging, but would also allow them

For peer review only

to gain knowledge and skills that would enable them to improve their own health and wellbeing.

> *"a practical kitchen/cooking session… would add* ***variety****"* (First Year).

> *"practical kitchen sessions would* ***provide great insight into how to prepare healthy meals for students as well as future doctors****. Students are known for eating very poor diets and it affects our physical and mental health too"* (Fourth Year).

However, other students expressed distaste for a practical kitchen session, questioning the feasibility of teaching in a kitchen and whether the skills gained would be useful in future clinical practice.

> *"a practical "kitchen session" could be very* ***difficult to organise*** *and the* ***quality of it is likely to be poor****"* (Fifth Year).

> *"I* ***don't really see how it is practical*** *to teach practical culinary skills [or]* ***what advantage this would confer****…* ***we aren't going to be cooking patients' food****"* (Fourth Year).

Some students suggested that opportunities to shadow HCPs with expertise in diet and nutrition would be a valuable learning experiences.

> *"****shadow a dietitian****"* (Fifth Year).

> *"sitting in with* ***diabetic nurses*** *and going through some of the* ***dietary restrictions*** *[for] patients"* (Fifth Year).

Other students expressed preference for more traditional teaching methods.

> *"a* ***formal tutorial*** *on the…* ***DAFNE*** *[Dose Adjustment For Normal Eating]* ***diet*** *would [be] even more useful"* (Fifth Year).

Students suggested how CM could be integrated into the current MBBS curriculum. Students associated CM with motivational interviewing, which is covered more broadly elsewhere in the MBBS curriculum outside of the context of diet. Motivational interviewing involves exploring patients' ideas, concerns and expectations regarding a lifestyle habit, enabling patients to recognise the need for behaviour change, establishing how motivated patients are to change their behaviour and supporting patients in achieving goals for health and wellbeing improvement.

> *"****how to elicit behaviour change****"* (Second Year).

> *"lifestyle advice [teaching] could be* ***combined with the patient centred pathway teaching*** *in year 5* ***on motivational interviewing****"* (Fifth Year).

Some students felt that it would be appropriate to dedicate up to one day to learning CM and explained that medical students are likely to already have a good understanding of what constitutes a healthy diet from teaching received elsewhere in the MBBS curriculum.

> *"CM* ***day****…* ***any more than that would be excessive****.* ***Most students will already know the basics of constructing a good diet****"* (Fifth Year).

There were a range of opinions on which year(s) of the MBBS curriculum the CM course should be delivered in.

> *"integrate* ***preclin[ical]*** *science into FNM ['Fluids, Nutrition and Metabolism' - a module taught in year one of the MBBS curriculum], then teach clinically in* ***year 5****"* (Fourth Year).

For peer review only

*"take this module as a **final year** medical student… as an **optional extra module**"* (Fifth Year).

Core Theme 2: The Relationship Between Food and Health

Students described the links between diet, social factors and health.

Sub-theme 1: Diet and Physical and Mental Health

Students recognised a link between diet and physical and/or mental health.

*"nutrition and its impact on a patient's **physical** and **mental wellbeing**"* (Third Year).

Students felt that diet plays a central role in preventing, treating and managing disease, and alleviating symptoms due to ill-health.

"*Using knowledge of nutrition and lifestyle factors associated with diet to both **prevent disease** and to **improve morbidity and mortality***" (Fifth Year).

Students expressed a desire for the CM teaching to include teaching on mental health problems related to eating.

*"it is really important that [the CM course] more than just a focus[es] on **'healthy' diets and weight** loss, but also to focus[es] on **eating disorders** including **anorexia**, **bulimia**, **binge eating disorder** and other **behavioural disorders**"* (Fourth Year).

Students suggested the need for doctors to recognise individual patients' relationship with food, and to promote the health benefits of a balanced diet, rather than labelling foods as inherently good or bad.

*"**dieting** has been proven to have numerous **negative effects on health**. [CM teaching] should **focus [on] the health benefits of good diet** [and] should not… label foods as 'good' or 'bad' as this leads to **moral judgement**, **restriction** and **reactive bingeing** on 'forbidden' foods"* (Fourth Year).

Sub-theme 2: Social Factors

Students suggested factors that determine diet and health, including ethnic and cultural background, socio-economic status and level of education.

"***level of education**… affects your health*" (Fourth Year).

"***tailor** [patients' diets] to **lifestyle**, **ethnic**, **economic/ social factors***" (Third Year).

Students stressed the importance of HCPs identifying and addressing social factors to identify how to best help patients to improve their diet and health.

"*understanding… [patients'] **individual barriers to healthy eating***" (Second Year).

Students also believed that doctors should have an understanding of cultural variation in diets to be able to provide patient-centred dietary advice. Students suggested that this will improve patients' understanding of diet and health and may increase patients' adherence to dietary advice.

*"my mum **cooks traditional Indian food on a daily basis** and hence [my parents] **prefer seeing an Indian GP when they go to the doctors**"* (Third Year).

Core Theme 3: Evidence-Based Medicine

For peer review only

Some students expressed concern over the extent of poorly evidenced diet and nutrition information that is publicly available.

> *"I'm… worried about the* ***quality*** *and the* ***evidence base*** *for [CM].* ***Diet and nutrition are****…* ***affected by****…* ***low-quality research****"* (Fifth Year).

Students suggested that the course should teach them evidence-based knowledge and skills to support patients in making dietary changes.

> *"****evidence-based practice*** *of influencing health through lifestyle changes in food consumption"* (First Year).

INTEGRATING CM KNOWLEDGE INTO CLINICAL PRACTICE

Table 3: *Results from quantitative analysis of responses to the Likert-type questions. Results are presented as number (percentage).*

**Statement**		**Pre-Clinical (n=81)**	**Clinical (n=99)**	**Total (n=180)**	**p-value**
**I am able to facilitate a discussion with patients about diet and/or dietary change.**	Disagree	39 (48.1)	25 (25.3)	64 (35.6)	p = 0.005
	Neutral	28 (34.6)	44 (44.4)	72 (40.0)	
	Agree	14 (17.3)	30 (30.3)	44 (24.4)	
**I am able to take dietary history from a patient.**	Disagree	59 (71.6)	43 (43.4)	101 (56.1)	p = 0.001
	Neutral	14 (17.3)	31 (31.3)	45 (25.0)	
	Agree	9 (11.1)	25 (25.3)	34 (18.9)	
**I am able to apply principles, methods and knowledge relating to nutrition to medical practice and integrate these into patient care.**	Disagree	52 (64.2)	56 (56.6)	108 (60.0)	p = 0.498
	Neutral	22 (27.2)	30 (30.3)	52 (28.9)	
	Agree	7 (8.6)	13 (13.1)	20 (11.1)	
**I am able to provide dietary advice for patients with varying cultural, social and economic needs.**	Disagree	57 (70.4)	63 (63.6)	120 (66.7)	p = 0.617
	Neutral	18 (22.2)	26 (26.3)	44 (24.4)	
	Agree	6 (7.4)	10 (10.1)	16 (8.9)	
**I believe that learning CM is important for my future clinical practice.**	Disagree	6 (7.4)	4 (4.0)	10 (5.6)	p = 0.482
	Neutral	10 (12.3)	10 (10.1)	20 (11.1)	
	Agree	65 (80.2)	85 (85.9)	150 (83.3)	

PROVISION OF CM TEACHING

62% of students reported having received no prior CM teaching (see Figure 2).

68% pre-clinical and 77% clinical students were dissatisfied with the quality of teaching understood to be relevant to CM provided by the medical school. 72% of pre-clinical and 84% of clinical students were dissatisfied with the current quantity of teaching understood to be relevant to CM provided by the medical school (see Figure 3).

FEATURES OF CM TEACHING

For peer review only

Students were asked to suggest features of an ideal CM course, including course content and delivery. Students felt that it is most appropriate for a CM course to include teaching on weight management and portion control (157 responses, see Figure 4), followed by the types of diet and their evidence base (156 responses). Nutrition psychology was the least popular topic (128 responses).

Students felt that the most appropriate methods of providing CM teaching are problem-based learning (140 responses, see Figure 2) and small-group seminars/ tutorials (138 responses).

67% of students expressed a preference for CM to be taught in pre-clinical training (pre-clinical students 72%, clinical students 64%, p = 0.257).

Discussion

Most medical students who participated in this study felt that learning CM is integral to their future role as a doctor. However, a few students anticipated that the task of dietary counselling would be delegated to other HCPs, such as dietitians. The latter views are contradicted by literature that suggest the health benefits for patients when dietary advice is given by doctors, especially in General Practice[17–19]. This supports the delivery of CM teaching in the year five General Practice placement in the MBBS curriculum. Also, the GMC states that all newly-graduated doctors should be capable of providing basic dietary advice.[2]. Furthermore, teaching on all three domains of nutrition education (basic nutritional science, public health and clinical nutrition) is recommended by the European Society for Clinical Nutrition and Metabolism (ESPEN).[20]

Although 83% of students felt that learning CM is important for their future clinical practice, 67% did not feel confident in their knowledge, nor competent to provide specific dietary advice These findings are similar to that of an Australian study[5], which found that the majority of pre-clinical medical students felt it was important for doctors to have an understanding of nutrition in relation to conditions, such as coeliac disease, but fewer than half were confident in applying their knowledge to clinical practice.

Clinical students reported higher levels of self-perceived confidence and competence in their abilities to conduct dietary counselling compared to pre-clinical students. This may be because clinical placements predominantly occur during years 4–6 of the MBBS curriculum, during which students are more likely to observe HCPs discussing diet and health with their patients. Students on these placements may have also been offered opportunities to practice dietary counselling with patients. The variation in clinical exposure and availability of practical learning opportunities may have contributed to the differences in self-reported capabilities between these two groups. One aim of placing the CM teaching in the General Practice placement is to enable students to draw upon their experiences of encounters with patients in General Practice and to share these experiences in group discussion about how to approach patient-based problems and cases effectively. Another aim is to allow students to apply the dietary counselling skills taught during the CM course to future interactions with patients, including during their on-going placements in General Practice.

This overall low self-perceived confidence and competence reported by questionnaire respondents may be related to their dissatisfaction with the quality and quantity of teaching

For peer review only

perceived to be relevant to CM in the MBBS curriculum at the time of this study. This may suggest that this cohort of students had particular interest in, and therefore concern for, undergraduate medical education.

The findings from this study suggest that key components of a CM teaching course placed in the clinical part of an undergraduate medical curriculum are: explaining the clinical and professional relevance of drawing upon patient cases from General Practice placements to aid CM learning and supporting medical students in developing an understanding of how this knowledge might be applied in clinical practice to maximise opportunities for supporting health promotion and managing ill health. These findings were subsequently used to inform the development of a CM course at a UK medical school, as described below.

Strengths and Limitations

A strength of this study is that the questionnaire waspiloted by two undergraduate medical students, who checked the readability. The students also confirmed that the questions would enable medical students' teaching preferences and expectations for CM learning to be gathered. This validation step increased the reliability of the questionnaire.

Moreover, JX and ShP conducted the early stages of thematic analysis independently to allow for a wider breadth of interpretations to be captured. The analysis team (JX, ShP and SP) then synthesised and discussed the interpretations before reaching an agreement.

Additionally, the questionnaire response rate was low (11%) which limits the generalisability of the findings. The low response rate may be due to the fact that CM is a relatively new term in medicine and is not commonly used amongst HCPs in the UK. This may be because CM is not yet included on most UK medical schools' curricula. The authors anticipated that students with prior understanding and/or interest in CM, were more likely to complete the questionnaire. Therefore, in order to encourage students to participate, advertisements for the questionnaire, as well as the PIS, emphasised that all medical students were eligible to take part in the study and that a wide breadth of experiences and perspectives, including no prior understanding of CM, were welcome. Even with this additional encouragement, the response rate remained low. However, theoretical saturation was judged to have been reached after coding the first 148 responses in an interim analysis, implying that sufficient qualitative data had been gathered for the views of students to be represented in the free-text responses. This strengthened the credibility of the study.

Another limitation is that the questionnaire responses were received from one UK medical school only. Factors such as differences between medical school curricula may limit the transferability of the study findings to other medical schools.

Implications

The findings from this study have been used to inform the development of a CM course for fifth-year undergraduate students at a UK medical school during their General Practice placement.

A number of recommendations can be made:

CM Course Content

For peer review only

1. Some study participants had never come across the term CM. The CM course teaches students about the key principles and aims of CM.

CM Teaching Methods

2. The CM course is currently integrated into the General Practice placement of the penultimate year of the MBBS curriculum to allow students to draw explicit links between CM knowledge and patients requiring dietary advice whom they encounter in General Practice. The course provides hands-on culinary skills training, evidence-based teaching in small group-seminars/tutorials, and patient-based integration of this knowledge in case-based discussions. These teaching methods were chosen as a result of the preferences expressed by students in this study. According to an American study[8], medical students' nutrition knowledge and food identification skills improved following completion of a CM course with culinary skills training, suggesting that practical learning is an effective teaching method.
3. The course creators prepared relevant case discussions to maximise clinical relevance. Course facilitators encourage students to prepare their own summaries of relevant cases encountered during their General Practice placements, supporting students to think about how they will apply the teaching in their future patient interactions following the course.

Integrating CM Teaching into the Clinical Curriculum

4. Students expressed a desire for more CM teaching in pre-clinical training and for an optional student-selected module in clinical training. The latter approach is offered at another UK medical school.[11] However, the course creators felt that it was important for all students to have a basic grounding in this knowledge and practical skills, whatever their future career intentions, hence the decision to make CM teaching compulsory in the MBBS curriculum. Other medical schools could use this as an exemplar of how to introduce CM into the clinical curriculum.

The questionnaire used in this study has been adapted into a post-course questionnaire to gather students' feedback on their CM learning experiences. The course is being continually evaluated to assess if students' learning needs are being satisfactorily addressed. The post-course feedback data will be analysed in a separate study.

Furthermore, a repeat of this questionnaire study could be conducted at UK medical schools who do not currently provide CM teaching. This will enable those medical schools to explore how their students feel about learning CM as part of their undergraduate training and will gather students' teaching preferences and expectations.

Conclusion

This cross-sectional quantitative study has provided insight into medical students' understanding of and attitudes towards learning CM. The study has highlighted students' perceptions on how they feel CM knowledge and skills might help them to fulfil the GMC's Outcomes for Graduates and their future role as doctors. Students reported a lack of confidence in their dietary counselling skills and felt that they could benefit from further

For peer review only

integrated and clinically-relevant teaching. Students' preferences for CM teaching have been taken into consideration in the development of a CM course for fifth-year undergraduate students at a UK medical school during their General Practice placements. Future research in the area of CM in undergraduate medical education is required to confirm whether the findings from this study are transferrable to other medical schools.

Acknowledgements

We would like to thank Professor Ann Griffin (Director of the Research Department of Medical Education (RDME) of UCL Medical School), for granting us permission to recruit study participants from UCLMS, and Professor Elizabeth Murray (Head of the Research Department of Primary Care and Population Health at UCL), for approving the study protocol. We are thankful to the National Institute for Health Research School for Primary Care Research (NIHR SPCR) for providing a grant to fund this research project. Thank you to UCL Student Data Analyst for providing us with the number of medical students enrolled at UCL in the academic year 2018/19. Gratitude must also be expressed to Miss Valeriya Kopanitsa and Dr Deepash Hosadurg for reviewing the preliminary questionnaire. Finally, we are thankful to all the study participants for taking the time to complete the questionnaire and for share their invaluable thoughts on CM teaching.

Contributions

JX was the main author who lead the study design, data collection and analysis and write-up. SP (fourth author) was the chief investigator. SP and ShP (second author) co-supervised the study and contributed towards designing the study, participant recruitment and data analysis. VV (third author) supervised the statistical analysis. All authors provided input in the write-up and have approved the final manuscript.

Funding

This work was supported by the National Institute for Health Research School for Primary Care Research (NIHR SPCR) grant number 156780. VV receives Seedcorn Funding from the NIHR SPCR.

Competing Interests

None declared.

Patient Consent for Publication

Not required.

Ethics Approval

University College London Ethics (ref.12471/002).

Provenance and Peer Review

This paper was not peer reviewed prior to submission.

Data Sharing Statement

The data generated from this study is not suitable for sharing beyond that contained within the report. Further information can be obtained from the corresponding author.

For peer review only

References

1. La Puma J. What Is Culinary Medicine and What Does It Do? Popul Health Manag. 2016 Feb;19(1):1–3.
2. General Medical Council. Outcomes For Graduates 2018. 2018; Available from: https://www.gmc-uk.org/-/media/documents/dc11326-outcomes-for-graduates-2018_pdf-75040796.pdf
3. Campbell D. Doctors 'know too little about nutrition and exercise' [Internet]. The Guardian; 2016 [cited 2019 Feb 17]. Available from: https://www.theguardian.com/society/2016/oct/19/doctors-know-too-little-about-effects-of-nutrition-and-exercise
4. Crowley J, Ball L, Hiddink GJ. Nutrition in medical education: a systematic review. Lancet Planet Heal. 2019 Sep;3(9):e379–89.
5. Perlstein R, McCoombe S, Shaw C, Nowson C. Medical students' perceptions regarding the importance of nutritional knowledge and their confidence in providing competent nutrition practice. Public Health [Internet]. 2016;140:27–34. Available from: http://www.sciencedirect.com/science/article/pii/S0033350616302207
6. Telegraph Reporters. GP training is outdated and needs to teach young doctors about nutrition, experts claim [Internet]. Telegraph. 2018 [cited 2019 Feb 17]. Available from: https://www.telegraph.co.uk/news/2018/03/25/gp-training-outdated-needs-teach-young-doctors-nutrition-experts
7. Conroy MB, Delichatsios HK, Hafler JP, Rigotti NA. Impact of a preventive medicine and nutrition curriculum for medical students. Am J Prev Med. 2004 Jul;27(1):77–80.
8. Pang B, Memel Z, Diamant C, Clarke E, Chou S, Gregory H. Culinary medicine and community partnership: hands-on culinary skills training to empower medical students to provide patient-centered nutrition education. Med Educ Online. 2019 Dec;24(1):1630238.
9. Ring M, Cheung E, Mahadevan R, Folkens S, Edens N. Cooking Up Health: A Novel Culinary Medicine and Service Learning Elective for Health Professional Students. J Altern Complement Med. 2019 Jan;25(1):61–72.
10. Schlair S, Hanley K, Gillespie C, Disney L, Kalet A, Darby PC, et al. How Medical Students' Behaviors and Attitudes affect the Impact of a Brief Curriculum on Nutrition Counseling. J Nutr Educ Behav [Internet]. 2012;44(6):653–7. Available from: http://www.sciencedirect.com/science/article/pii/S149940461100529X
11. Culinary Medicine UK. Culinary Medicine UK [Internet]. 2020 [cited 2020 May 12]. Available from: https://culinarymedicineuk.org
12. NVivo qualitative data analysis Software, Version 12. QSR International Pty Ltd; 2018.
13. Braun V, Clarke V. Using thematic analysis in psychology. Qual Res Psychol [Internet]. 2006 Jan 1;3(2):77–101. Available from: https://www.tandfonline.com/doi/abs/10.1191/1478088706qp063oa
14. Stuckey H. The second step in data analysis: Coding qualitative research data . J Soc Heal Diabetes [Internet]. 2015 Jan 1;3(1):7–10. Available from: http://www.joshd.net/article.asp?issn=2321-0656

15. Hewitt-Taylor J. Use of constant comparative analysis in qualitative research. Nurs Stand. 2001 Jul;15(42):39–42.

16. IBM Corp. IBM SPSS Statistics for Macintosh, Version 25.0. Armonk, NY: IBM Corp; 2017.

17. Willaing I, Ladelund S, Jørgensen T, Simonsen T, Nielsen LM. Nutritional counselling in primary health care: a randomized comparison of an intervention by general practitioner or dietician. Eur J Cardiovasc Prev Rehabil [Internet]. 2004;11(6):513–20. Available from: https://doi.org/10.1097/01.hjr.0000152244.58950.5f

18. Pritchard DA, Hyndman J, Taba F. Nutritional counselling in general practice: a cost effective analysis. J Epidemiol Community Health [Internet]. 1999 May;53(5):311–6. Available from: https://www.ncbi.nlm.nih.gov/pubmed/10396539

19. Sacerdote C, Fiorini L, Rosato R, Audenino M, Valpreda M, Vineis P. Randomized controlled trial: effect of nutritional counselling in general practice. Int J Epidemiol [Internet]. 2005;35(2):409–15. Available from: https://doi.org/10.1093/ije/dyi170

20. Cuerda C, Muscaritoli M, Donini LM, Baqué P, Barazzoni R, Gaudio E, et al. Nutrition education in medical schools (NEMS). An ESPEN position paper. Clin Nutr [Internet]. 2019;38(3):969–74. Available from: http://www.sciencedirect.com/science/article/pii/S0261561419300500

Figure 1: *Results from quantitative analysis of responses to the single choice question 'Which year of the medical (MBBS) programme are you currently in?'.*

Figure 2: *A comparison of the results from quantitative analysis of responses to two multiple choice questions: 'What types of CM training have you received so far in the MBBS curriculum?' and 'Select the 3 most appropriate teaching methods for a CM course'.*

Figure 3: *Results from quantitative analysis of responses to the Likert-type questions: 'To what extent do you agree with the statements: "I am satisfied with the QUALITY of CM training that my medical school provides"? and 'To what extent do you agree with the statement "I am satisfied with the QUANTITIY of CM training that my medical school provides"?'.*

Figure 4: *Results from quantitative analysis of responses to the multiple choice question 'Which topics and/or skills do you think should be taught on the CM course?'.*

For peer review only

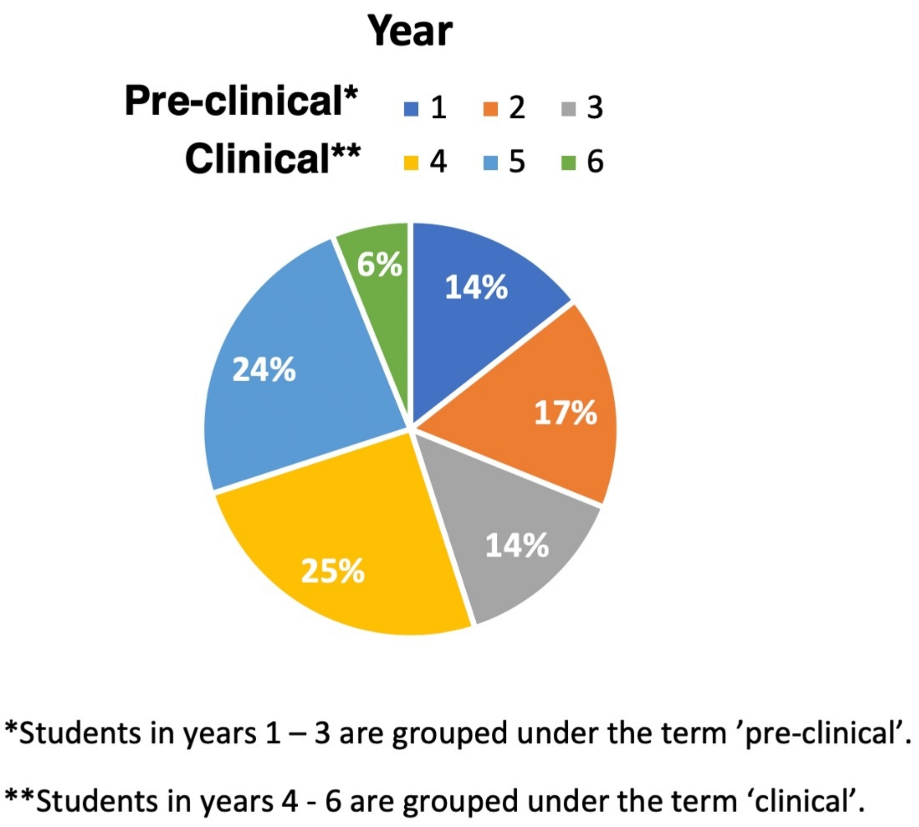

Figure 1: Results from quantitative analysis of responses to the single choice question ‘Which year of the medical (MBBS) programme are you currently in?'.

210x185mm (144 x 144 DPI)

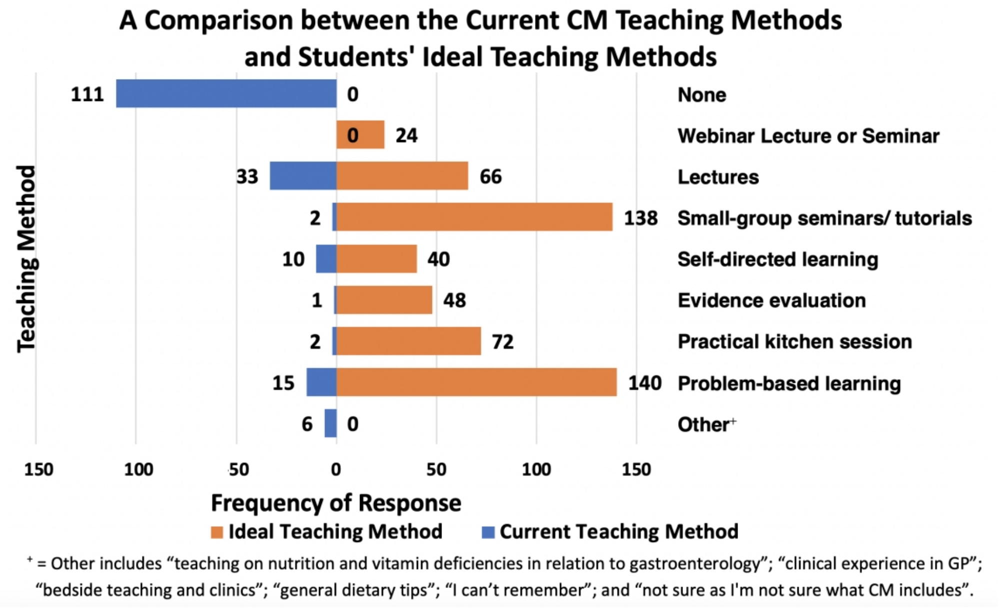

Figure 2: A comparison of the results from quantitative analysis of responses to two multiple choice questions: 'What types of CM training have you received so far in the MBBS curriculum?' and 'Select the 3 most appropriate teaching methods for a CM course'.

412x250mm (144 x 144 DPI)

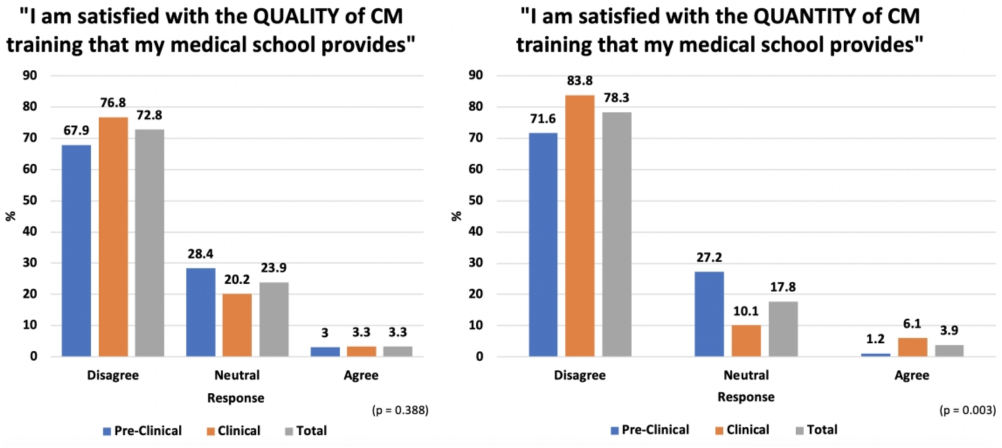

Figure 3: Results from quantitative analysis of responses to the Likert-type questions: ‘To what extent do you agree with the statements: “I am satisfied with the QUALITY of CM training that my medical school provides”? and ‘To what extent do you agree with the statement “I am satisfied with the QUANTITIY of CM training that my medical school provides”?’.

496x223mm (144 x 144 DPI)

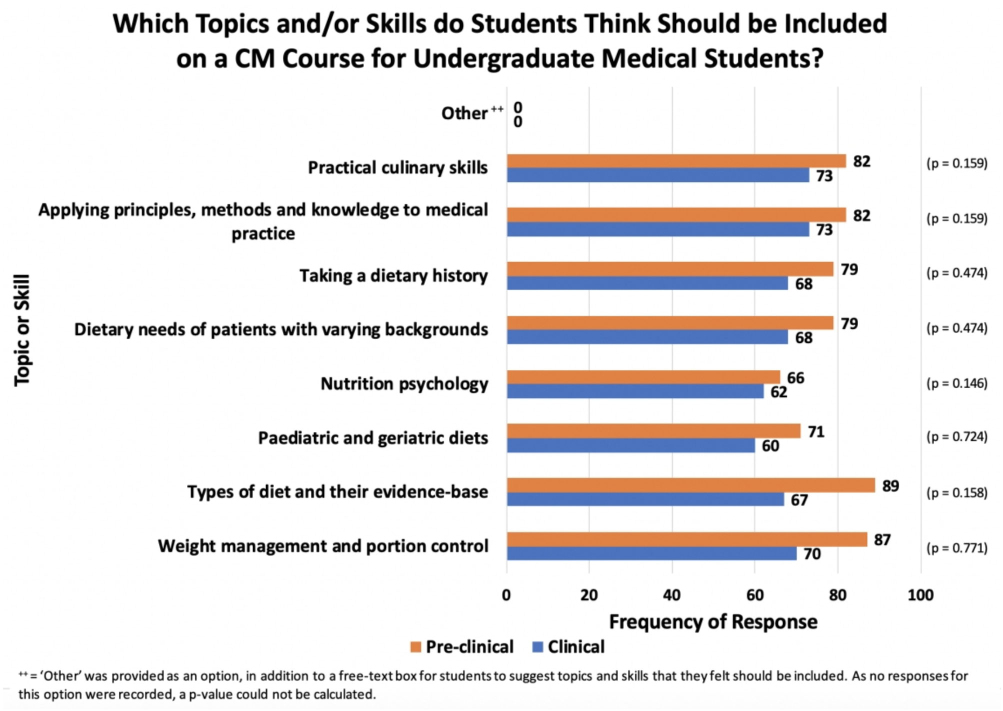

Figure 4: Results from quantitative analysis of responses to the multiple choice question 'Which topics and/or skills do you think should be taught on the CM course?'.

363x257mm (144 x 144 DPI)

A survey of the preferences and expectations of UK undergraduate medical students from a Culinary Medicine course.

Chief Investigator:
Dr Sophie Park

Supported by:

Sponsored by:
University College London (UCL)

Protocol version number and date:
Version 1
05/11/18

R&D / Sponsor Reference Number(s):

Not known at present.

Study Registration Number:
Not known at present.

For peer review only

For peer review only - http://bmjopen.bmj.com/site/about/guidelines.xhtml

PROTOCOL VERSIONS

Version Stage	Versions No	Version Date	Protocol updated & finalised by;	Appendix No detail the reason(s) for the protocol update
Current	Version 1	05/11/18	TBC	Appendix version 1

DECLARATIONS

The undersigned confirm that the following protocol has been agreed and accepted and that the investigator agrees to conduct the study in compliance with the approved protocol and will adhere to the Research Governance Framework 2005 (as amended thereafter), the Trust Data & Information policy, Sponsor and other relevant SOPs and applicable Trust policies and legal frameworks.

I (investigator) agree to ensure that the confidential information contained in this document will not be used for any other purposes other than the evaluation or conduct of the clinical investigation without the prior written consent of the Sponsor.

I (investigator) also confirm that an honest accurate and transparent account of the study will be given; and that any deviations from the study as planned in this protocol will be explained and reported accordingly.

For peer review only

Chief Investigator:

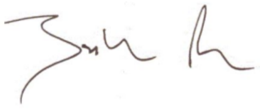

Signature: **Date** 06/11/2018

Print Name(in full): Sophie Park

Position: Director of UG Medical Education (Primary Care & Community)

On behalf of the Study Sponsor:

Signature:.. **Date....../....../.......**

Print Name(in full):...

Position:..

STUDY SUMMARY

**Identifiers**	
IRAS Number	Not known at present.
REC Reference No	Not known at present.
Sponsor Reference No	Not known at present.
Other research reference number(s) (if applicable)	Not applicable to this study.
Full (Scientific) title	A survey of the preferences and expectations of UK undergraduate medical students from a Culinary Medicine course.
Health condition(s) or problem(s) studied	UK medical students' preferences in an undergraduate Culinary Medicine course
Study Type i.e. Cohort etc	A quantitative study involving medical students completing a questionnaire.
Target sample size	330
**STUDY TIMELINES**	
Study Duration/length	November 2018 – February 2019.
Expected Start Date	30/11/18
End of Study definition and anticipated date	01/01/19
Key Study milestones	• Study submission<br>• Budget and contract to be finalised<br>• Recruitment of study participants<br>• Collecting data<br>• Statistical analysis of results<br>• Dissemination of results
**FUNDING & Other**	
Funding	National Institute for Health Research (NIHR) School for Primary Care Research (SPCR)
Other support	None
**STORAGE of SAMPLES (if applicable)**	
Human tissue samples	Not applicable to this study.
Data collected / Storage	Not applicable to this study.
**KEY STUDY CONTACTS**	Full contact details including phone, email and fax numbers
Chief Investigator	Dr Sophie Park

For peer review only - http://bmjopen.bmj.com/site/about/guidelines.xhtml

KEY ROLES AND RESPONSIBILITIES

SPONSOR: The sponsor is responsible for ensuring before a study begins that arrangements are in place for the research team to access resources and support to deliver the research as proposed and allocate responsibilities for the management, monitoring and reporting of the research. The Sponsor also has to be satisfied there is agreement on appropriate arrangements to record, report and review significant developments as the research proceeds, and approve any modifications to the design.

FUNDER: The funder is the entity that will provide the funds (financial support) for the conduction of the study. Funders are expected to provide assistance to any enquiry, audit or investigation related to the funded work.

CHIEF INVESTIGATOR (CI): Sophie Park is the chief investigator. The person who takes overall responsibility for the design, conduct and reporting of a study. If the study involves researchers at more than once site, the CI takes on the primary responsibility whether or not he/she is an investigator at any particular site.

The CI role is to complete and to ensure that all relevant regulatory approvals are in place before the study begins. Ensure arrangements are in place for good study conduct, robust monitoring and reporting, including prompt reporting of incidents, this includes putting in place adequate training for study staff to conduct the study as per the protocol and relevant standards.

The Chief Investigator is responsible for submission of annual reports as required. The Chief Investigator will notify the RE of the end of the study, including the reasons for the premature termination. Within one year after the end of study, the Chief Investigator will submit a final report with the results, including any publications/abstracts to the REC.

PRINCIPLE INVESTIGATOR (PI): Individually or as leader of the researchers at a site; ensuring that the study is conducted as per the approved study protocol, and report/notify the relevant parties – this includes the CI of any breaches or incidents related to the study.

PROJECT SUPERVISOR: Shoba Poduval has provided the principal researcher (Jessica Xie) with guidance at every stage of this project.

STATISTICIAN: a statistician based in the UCL department of Primary Care and Population Health will contribute to the data analysis.

PATIENT AND PARTICIPANT INVOLVEMENT (PPI): Two UCL medical students (one in fifth-year and one in third-year) will be recruited by the principal researcher through convenience sampling. PPI will be asked to provide feedback on the questionnaire questions, the recruitment flyer and will also be involved in the data analysis.

For peer review only - http://bmjopen.bmj.com/site/about/guidelines.xhtml

KEY WORDS

Culinary medicine, nutrition, diet, undergraduate, medical student, medical school.

LIST OF ABBREVIATIONS

AE	Adverse Event
AR	Adverse Reaction
CI	Chief Investigator
CRF	Case Report Form
CRO	Contract Research Organisation
DMC	Data Monitoring Committee
GAfREC	Governance Arrangement for NHS Research Ethics
HTA	Human Tissue Authority
IB	Investigator Brochure
ICF	Informed Consent Form
MD	Medical Device
ISRCTN	International Standard Randomised Controlled Studies Number
PI	Principle Investigator
PIS	Participant Information Sheet
QA	Quality Assurance
QC	Quality Control
RCT	Randomised Clinical Study
REC	Research Ethics committee
SAR	Serious Adverse Reaction
SAE	Serious Adverse Event
SDV	Source Data Verification
SOP	Standard Operating Procedure
SSI	Site Specific Information
TMF	Trial Master File

For peer review only

CONTENTS

1 INTRODUCTION 8

2 BACKGROUND AND RATIONALE 8

3 OBJECTIVES 8

3.1 Primary Objective 14

3.2 Secondary Objectives 14

4 STUDY DESIGN 15

5 STUDY SCHEDULE 19

6 CONSENT 20

7 ELIGIBILITY CRITERIA 21

7.1 Inclusion Criteria 21

7.2 Exclusion Criteria 21

8 RECRUITMENT 21

9 STATISTICAL METHODS 21

10 PATIENT AND PUBLIC INVOLVEMENT (PPI) 22

11 FUNDING AND SUPPLY OF EQUIPMENT 22

12 DATA HANDLING AND MANAGEMENT 22

13 MATERIAL/SAMPLE STORAGE 23

14 PEER AND REGULATORY REVIEW 24

15 ASSESMENT AND MANAGEMENT OF RISK 24

16 RECORDING AND REPORTING OF EVENTS AND INCIDENTS 24

16.1 Definitions of Adverse Events .... **Error! Bookmark not defined.**

16.2 Assessments of Adverse Events .... **Error! Bookmark not defined.**

16.3 Recording adverse events .... **Error! Bookmark not defined.**

16.4 Procedures for recording and reporting Serious Adverse Events .... **Error! Bookmark not defined.**

16.5 Serious Adverse Events that do not require reporting .... **Error! Bookmark not defined.**

16.6 Reporting Urgent Safety Measures .... **Error! Bookmark not defined.**

16.7 Protocol deviations and notification of protocol violations **Error! Bookmark not defined.**

For peer review only - http://bmjopen.bmj.com/site/about/guidelines.xhtml

16.8 Reporting incidents involving a medical device(s) (if applicable) .... **Error! Bookmark not defined.**

16.9 Trust incidents and near misses.. **Error! Bookmark not defined.**

17 MONITORING AND AUDITING.. 24

18 TRAINING 24

19 INTELLECTUAL PROPERTY 24

20 INDEMNITY ARRANGEMENTS 24

21 ARCHIVING 25

22 PUBLICATION AND DISSEMINATION POLICY 25

23 REFERENCES 25

24 APPENDICES 29

For peer review only

1 INTRODUCTION

Culinary Medicine is defined by La Puma and Marx (2008) as an "evidence-based field in Medicine that blends the art of food and cooking with the science of medicine". Culinary Medicine courses typically allow students to receive teaching about nutrition and its health-promoting properties, read the relevant literature, gain hands-on cooking experiences and apply the knowledge that they have learnt to provide appropriate dietary advice for patients in the clinical setting (Bendici, 2018).

The importance of nutrition and diet in undergraduate medical education teaching across all disciplines has long been under-recognized.

University College London (UCL) Medical School is in currently developing a Culinary Medicine course for its undergraduate students.

Research Question

A survey of the preferences and expectations of UK undergraduate medical students from a Culinary Medicine course.

Method

A quantitative questionnaire study with undergraduate medical students at a London medical school (see Appendix 1)

Aim

To determine University College London (UCL) medical students' learning needs and preferences in the area of Culinary Medicine with a view to informing development of a Culinary Medicine course at UCL.

Expected Outcomes

This study will determine undergraduate medical students' understanding of Culinary Medicine and their preferences and expectations of a Culinary Medicine course in their medical programme.

The results of this study will be used in the development of a Culinary Medicine course at UCL medical school for undergraduate students.

2 BACKGROUND AND RATIONALE

Introduction to Area of Research

The UK is reported to be the most obese country in Western Europe. 27% of the UK population are classified as obese - a body mass index of 30 and above (The Organisation for Economic Co-operation and Development, 2017), and approximately one-third of children aged 2 to 15 in the UK are overweight or obese (Buck, 2018) . It is well-known that obesity is a risk factor for chronic diseases, such as cancer and heart disease. These diseases may be prevented or, in the case of Type

For peer review only - http://bmjopen.bmj.com/site/about/guidelines.xhtml

2 diabetes mellitus, reversed through a planned and controlled diet consisting of healthy nutrients. There is also evidence to suggest that diet is important for mental health (Sarris *et al.*, 2015; Jacka *et al.*, 2017).

The General Medical Council (GMC) (2018) requires UK Medical School graduates to be competent in recognising ill health as a result of poor nutrition, and applying the principles, methods and knowledge of diet to medical practice and integrating these into patient care.

However, it is reported that the majority doctors in the UK feel incompetent to provide patients with lifestyle advice (The British Broadcasting Corporation, 2010; Campbell, 2016).

Dr Michael Mosley, presenter of the BBC One show 'Trust Me I'm A Doctor', stated that nutrition is "not part of the traditional training", claiming that he "learnt almost nothing about nutrition" when he was at Medical School. Womersley and Ripullone (2017), University of Cambridge graduate medical students, believe that the medical profession view this field as an unscientific subject that lies in the domain of dieticians, rather than doctors.

The evidence-base of Culinary Medicine has changed dramatically in the past few decades (Crawford and Aspry, 2016). There is growing interest in the relationship between food, eating, cooking and health and wellbeing in the medical profession and also in the public. For example, adopting an anti-inflammatory diet has been found to be as, or even more, effective than prescription medication for arthritis (Adam *et al.*, 2003) and nutritional support has been proven to improve the clinical outcomes of surgical patients (Ward, 2003; Coles, 2013; Tappenden *et al.*, 2013).

The interest for Culinary Medicine in the UK has grown in the recent years. In 2017, Nutritank was founded - an online, student-led organisation created by medical students for medical students to learn Culinary Medicine. The organisation has expanded and to date exists as a university society at approximately 15 UK universities (Dillon, 2018). Dr Rupy Aujla, founder of The Doctor's Kitchen, created the UK's first Culinary Medicine course, adapted from the course offered at Tulane Medical School, USA. The Royal College of General Practitioners accredited four of Dr Aujla's Culinary Medicine modules for inclusion among the optional courses doctors can take for their continuing professional development (CPD) requirement. In February 2018, the first course Culinary Medicine course was delivered in association with Westminster Kingsway Culinary school and in July 2018, a four-week Culinary Medicine course was offered as a student-selected component to students of Bristol University (Morrish, 2018; Moorhouse, 2018; Leith, 2018).

However, Culinary Medicine is more well-established in the USA compared to the UK. 25% of US Medical Schools have adopted Culinary Medicine programmes (Bendici, 2018). These courses are either offered to students as elective courses or are integrated as a compulsory component into the medical programme.

Culinary Medicine remains a non-mandatory component of UK Medical School curricula and many specialist training courses in the latter part of the junior doctor training programme (The British Broadcasting Corporation, 2010). One medical student of Bristol University claims that students are taught as little as 10 to 24 hours over five to six years in medical school Culinary Medicine (Dillon, 2018).

Current Literature

For peer review only - http://bmjopen.bmj.com/site/about/guidelines.xhtml

PubMed, Cochrane Review, Web of Science and Embase were used to perform a literature search (see Appendix 2). Google Scholar, the British Medical Journal (BMJ) and the student BMJ were also used to search for relevant studies. 8 studies were identified that are relevant to the proposed study (see Appendix 3). Four themes arose from the literature search: dietary knowledge, attitudes towards diet, confidence in providing dietary advice and methods of teaching Culinary Medicine.

Dietary Knowledge

Hargrove *et al.* (2017) conducted a two-part study: a descriptive cross-sectional study to evaluate 424 first- and second-year students' dietary knowledge and attitudes towards dietary counselling and a questionnaire study to assess students' attitude and knowledge of the role of food in Medicine. Their study findings suggest that only 11.9% of study participants were aware of the current dietary reference intakes, implying that the majority of the students did not have knowledge of the nutritional needs of a healthy person. However, the ability to generalise this finding to all medical schools is limited because the study sample was homogenous – participants were sampled from Ohio University Heritage College of Osteopathic Medicine (OU-HCOM).

Third- and fourth-year medical students in a US study conducted by Cooke *et al.* (2017) explained that, with their current level of knowledge, they would not be prepared to counsel patients: "When (my future patients) ask me something about nutrition, I'm going to have to Google (it)… because I don't feel like I have a very solid foundation" (p.158). These students seemed unsure about the treatment and prevention of obesity. The findings of this study may suggest that these US medical students lack knowledge, and/or confidence in their knowledge, of nutrition. If medical students feel incompetent in Culinary Medicine, their personal interest in this field may decrease and they may be less likely to discuss the topic with patients because they feel that they are not confident in discussing diet and/or dietary change with patients. This may suggest to healthcare professionals and patients that Culinary Medicine is a less important component of the clinical consultation. Moreover, since Cooke *et al.* (2017) conducted their study with a small number (78) of US medical students who had volunteered to participate in the study, the study participants may have been more interested in, and therefore may be more knowledgeable about, Culinary Medicine and/obesity than a typical US medical student. Therefore, this decreases the likelihood that the study sample is representative of the US medical student population.

In the USA, Mlodinow and Barrett-Connor (1989) investigated physicians' and 24 medical students' knowledge of diet. Their study findings included that physicians are more knowledgeable about diet in relation to coronary heart disease, rather than caries, whereas it was the opposite case for medical students. This could reflect that these physicians believe that caries topic lies in the domain of dentists, rather than doctors.

Attitude towards Diet

The results of a questionnaire study conducted by Schlair *et al*. (2012) suggest that medical students' behaviours may influence their attitudes towards, and knowledge of, nutrition and ability to provide dietary counselling. Students with greater dietary self-efficacy and greater fruit and vegetable intake appeared to be more likely to have higher dietary counselling confidence and competence. Therefore, this may imply that promoting healthy lifestyle amongst medical students and professionals could improve their own wellbeing, which in turn may improve the wellbeing of their

For peer review only - http://bmjopen.bmj.com/site/about/guidelines.xhtml

patients. Survey questions were selected from previously validated surveys and the response rates were high (69% and 75% for responses to the pre-curriculum and post-curriculum surveys respectively), suggesting that the results are valid. However, the results of this questionnaire should be interpreted with caution because the sample size was small (111 and 121 students completed the pre-curriculum and post-curriculum surveys respectively) and there was a possibility of recall bias during completion of the questionnaires.

Consistent with the study conducted by Hargrove *et al.* (2017), whereby 68% of 424 first- and second-year US medical students rated dietary education as ‘very important’, in an Australian study conducted by Perstein *et al.* (2016), the majority (79 – 99%) of 68 first-year and 63 second-year medical students agreed that it is important for medical graduates to understand dietary issues of disease related to conditions, such as elevated blood lipids. These results suggest that these medical students believe that qualified doctors should possess knowledge of Culinary Medicine and should be able to apply that knowledge to clinical scenarios. As the sample size for the study conducted by Perstein *et al.* (2016) was small (131 first- and second- year students and 66 third- and fourth-year students) and students were sampled from a single medical school (Deakin University), the credibility of the study may be limited.

Furthermore, 47% of participants (78% response rate from 424 medical students) in the US study conducted by Hargrove *et al.* (2017) expressed the importance of Culinary Medicine education as a means to provide educate patients, promote overall health and wellness, prevent disease and manage disease. This suggests that 47% of participants understand how to apply their knowledge of Culinary Medicine in the clinical setting to improve the health and wellbeing of patients.

Also, the results of a study conducted by Conroy *et al.* (2004) showed that students who undertake a preventive medicine and Culinary Medicine course may be more likely to adopt better health habits as a consequence.

<u>Confidence in Providing Dietary Advice</u>

According to the results of the Australian quantitative study conducted by Perstein *et al.* (2016), medical students’ confidence in demonstration of dietary knowledge increases with their medical training. For example 7% of 68 first- and 63 second-year students expressed confidence in their dietary knowledge in relation to chronic obstructive airways disease (COAD), whereas 19% of 66 third- and fourth-year students claimed to be confident in their dietary knowledge related to COAD. Third- and fourth-year students were most confident in in their dietary knowledge related to Type II diabetes (81%). Therefore, these students’ confidence in providing dietary advice in with clinical context depends on the specific condition or disease.

Perstein *et al.* (2016) also assessed third- and fourth-year medical students’ confidence in their knowledge of guidelines for the dietary management of diseases and their confidence in monitoring and evaluating patients’ diets. Less than 20% of the students felt confident in their knowledge of the dietary management of chronic diseases, including cardiovascular disease. Less than 20% felt confident in recommending food choices to patients with chronic disease and monitoring and evaluating patients’ diets. These results may suggest that students lack confidence in their abilities to provide patients with dietary advice A strength of this study is that follow-up reminder emails were sent to students requesting them to complete their surveys. However, despite this this effort,

For peer review only - http://bmjopen.bmj.com/site/about/guidelines.xhtml

Perstein *et al.* (2016) obtained a low response rate from study participants (24%), which may mean that these results ought to be interpreted with caution because poor response rate may indicate that the validity of the results are questionable.

Cooke *et al.* (2017) conducted a qualitative interview study to explore 78 third- and fourth-year US medical students' understanding of childhood obesity and their perceived educational deficits. The study participants reported possessing inadequate dietary counselling skills, expressing a lack of "confiden[ce] [in] helping patients overcome… barriers [to healthier lifestyles]" (p.159). The researchers took two measures to increase the trustworthiness of the data generated from this study: using non-judgemental and unbiased language when during the interviews and conducting participant validation. Although this study involved medical students of different ages (23 to 44 years old) and from different educational and demographic backgrounds (25 different US medical schools in 16 different states), the study sample may not be representative of the US medical student population because the sample size was small (78) and snowball sampling led to over-representation of the percentage of students pursuing careers in paediatrics and family medicine and some participant. Thus, the results may not be transferrable to other medical schools.

Conroy *et al.* (2004) evaluated the change in self-confidence in medical students after taking a Culinary and Lifestyle Medicine course. A questionnaire was used to assess the impact of a preventive medicine and nutrition course (PMN) on the confidence of 137 Harvard medical students in addressing patients' diet and exercise patterns and on their own lifestyle choices. Students' confidence in their ability to assess and counsel about diet and exercise significantly improved following the course (all p =0.001). Both the high response rates received for the baseline and follow-up surveys (98% and 86% respectively) and the fact that a comparison group was included to ensure that any changes in students' attitudes and behaviours were only attributable to participation in the course suggest that the results of the study may be valid. Observer bias was reduced as students were only identified by their unique confidential code, which was unknown to the investigators. However, the small sample size (137) limits the generalisability of the findings.

Additionally, it is important to note that data that is self-reported confidence is an subjective measure of counselling skills. Therefore, the results of studies conducted by Perstein *et al.* (2016), Cooke *et al.* (2017) and Conroy *et al.* (2004) may have been affected by measurement bias.

<u>Methods of Teaching Culinary Medicine</u>

In a study conducted in the USA by Walsh *et al.* (2011), 131 medical students completed a Preventative Medicine Nutrition course (content was delivered on a weekly basis over a period of three to four months) and 135 medical students received nutrition training as part of an integrated nutrition curriculum (content was taught over three half-days). There was no statistical difference in knowledge and attitude scores between students who completed the dedicated nutrition course compared with those who received nutrition training as part of an integrated nutrition curriculum. The results suggest that medical students were more satisfied with the dedicated nutrition course rather than the integrated curriculum, which may imply that the former course structure may provide a more positive, engaging learning experience for students. The results of a questionnaire study conducted by Walsh *et al.* (2011) suggest that medical students prefer a dedicated nutrition course (content delivered on a weekly basis over a period of three to four months) over an integrated curriculum (content taught over three half-days). There was no statistical difference in

For peer review only - http://bmjopen.bmj.com/site/about/guidelines.xhtml

knowledge and attitude scores between students who completed the dedicated nutrition course, compared to those who received nutrition training as part of an integrated nutrition curriculum. One strength of this study include was that five survey questions were included as validation items to control for possible response bias. However, the study findings should be interpreted with caution as one of the response rates (42.2%) was too low to meet the power calculation for statistically significant results at the 5% level and the sample size was small.

Engel *et al.* (1997) conducted a study in the USA to evaluate the third-year medical students', general care nurses' (with no diabetes-related dietary training) and dieticians' knowledge of diabetes nutrition prior to and after completing a computer-assisted diabetes instruction (CAI), lasting a mean of 27.9 minutes. Medical students' mean knowledge score prior to CAI was similar to the mean score of the nurses, but after competing CAI, medical students' mean score was similar to the mean score of dieticians. Engel *et al.* (1997) suggest that future dietary training for medical students should involve CAI because, as the course involves self-paced learning and technology, it is a practical and efficient method of teaching medical students. However, this study did not include a gold-standard method of dietary training to compare this teaching method to, which may reduce the usefulness of the study findings. Additionally, the sample size (41) was very small, which may suggest that the study sample is unrepresentative of the medical student population. A study involving a larger sample size is required to determine whether the results from the study conducted by Engel *et al.* (1997) are generalisable.

Medical students who participated in the study by Cooke *et al.* (2017) offered multiple methods for the delivery of Culinary Medicine courses, for example, seminars. Students also requested more hands-on learning opportunities, such as practicing counselling skills with children and families. This may imply that these students did not have a preference for a single method of teaching or that they prefer to be taught a module using a variety of teaching methods.

Cooke *et al.* (2017) suggest that Culinary Medicine courses taught in medical schools should contain more applicable nutrition and childhood obesity-specific knowledge and nutrition-related behavioural change counselling skill building through observation and practice.

Conroy *et al.* (2004) hypothesize that the active problem-based learning and counselling skills training components of the course were most effective in increasing students' confidence in counselling and that the self-assessment exercises engaged students' interest in the curriculum and encouraged them to make better lifestyle choices.

<u>Personal Interest in This Research Topic</u>

The researcher is a fourth-year medical student at UCL with a special interest in Culinary Medicine. In the 'Fluids, Nutrition and Metabolism' module in first year, the researcher learnt about the nutritional requirement of humans, how food is digested and how imbalanced diet is a risk factor for conditions such as Type II diabetes. Nutrition and diet were thus taught with a physiological approach, with little clinical context and no patient contact. In second year, the researcher attended seminars as part of the Cardiometabolic Patient Pathway component of the Clinical Professional Practice (CPP) module in which risk factors, including diet, for cardiovascular diseases were discussed. The researcher believes that healthcare professionals who are educated in Culinary

For peer review only - http://bmjopen.bmj.com/site/about/guidelines.xhtml

Medicine are empowered, and can consequently educate and empower their patients, to have greater control over improving their own health and wellbeing through positive, cost-effective lifestyle changes (Grewal, 2016; Vettel, 2018; Ferreira, 2018). Thus, the researcher aims to create a study that they hope will yield results that will contribute to the development and improvement of a Culinary Medicine course for undergraduate UK medical students.

Rationale for This Study

There is growing evidence of the health benefits of improving dietary health. The National Institute for Health and Care Excellence (NICE) has emphasised on the importance the role of healthcare professionals in educating patients in nutrition and lifestyle. NICE envisages that, in less than two years' time, nutrition could become the first-line intervention to tackle conditions such as heart disease and cancer by 2020 (Womersley and Ripullone, 2017).

Yet, the literature suggests that suggest that medical students, and even fully-qualified doctors, feel under-equipped to engage patients in conversations about this topic and provide dietary advice. The General Medical Council (GMC) (2018) requires UK Medical School graduates to be competent in recognising ill health as a result of poor nutrition, and applying the principles, methods and dietary knowledge to medical practice and integrating these into patient care.

UCL Medical School is currently in the process of creating an undergraduate Culinary Medicine course.

Research on medical students and nutrition education and Culinary Medicine training has been primarily conducted in the USA (Mlodinow, 1989; Engel, 1997; Walsh 2011; Schlair, 2012; Cooke, 2017). Therefore, the results of these studies may not be transferable to the UK. It will be useful to understand what UK medical students value in a Culinary Medicine course. Incorporating features that students value (including topics and learning strategies) will optimize students' potential to learn about the importance of diet in Medicine, and therefore their potential to apply the knowledge in a clinical setting to improve the health and wellbeing of their patients.

3 OBJECTIVES

3.1 Primary Objective

To determine UCL medical students' learning needs and preferences in the area of Culinary Medicine with a view to informing development of a culinary medicine course at UCL.

3.2 Secondary Objectives

- To determine undergraduate UCL medical students' understanding of Culinary Medicine and their preferences and expectations of a Culinary Medicine course in their medical programme.
- To determine the level of experiences and/or training in Culinary Medicine.

For peer review only - http://bmjopen.bmj.com/site/about/guidelines.xhtml

- To determine undergraduate medical students' perception of their current abilities to facilitate discussion about diet and provide dietary advice for patients.
- To evaluate the level of satisfaction of undergraduate medical students with the current quality and quantity of Culinary Medicine training delivered by UCL Medical School.

4 STUDY DESIGN

Study Design

	The overall aim of this study is to determine University College London (UCL) medical students' learning needs and preferences in the area of Culinary Medicine with a view to informing development of a culinary medicine course at UCL.			
	Methods			
	Design	Data collection	Data analysis	Outcome
To determine undergraduate medical students' understanding of Culinary Medicine and their preferences and expectations of a Culinary Medicine course in their medical programme.	Questionnaire	Free-text response	Thematic analysis	Themes for medical students' understanding of what Culinary Medicine is, what students think they should gain from an undergraduate Culinary Medicine course and what they think they should be taught
		Rating scale	Count the number of students who prefer each option. Calculate the proportion of students who select each option	Understanding of students' preferences for teaching methods
To determine the level of experiences and/or training in	Questionnaire	Multiple-choice questions	Count the number of responses for each option.	Students' experience and level of training in Culinary Medicine

For peer review only - http://bmjopen.bmj.com/site/about/guidelines.xhtml

Culinary Medicine			Calculate the proportion of students who select each option	
		Free-text response	Thematic analysis	Themes for medical students' experiences and/or training in Culinary Medicine
To determine undergraduate medical students' perception of their current abilities to facilitate discussion about diet and provide dietary advice for patients	Questionnaire	Likert scale	Calculate the proportion of students who strongly agree, disagree, are uncertain, agree or strongly agree that they are able to facilitate discussion about diet and provide dietary advice for patients	Understanding of whether students feel able to facilitate discussion about diet and provide dietary advice for patients
To evaluate the level of satisfaction of undergraduate medical students with the current quality and quantity of Culinary Medicine training delivered by UCL Medical School	Questionnaire	Likert scale	Calculate the proportion of students who strongly agree, disagree, are uncertain, agree or strongly agree that the current quality and quantity of Culinary Medicine training delivered by	Understanding of whether students think that the current quality and quantity of Culinary Medicine training delivered by UCL Medical School is adequate

For peer review only

			UCL Medical School is adequate	
		Multiple choice	Calculate the proportion of students who choose each option	Understanding of when and how students think Culinary Medicine training by UCL Medical School should be delivered

In the table above, the researcher has illustrated how the study design, data collection, data analysis and outcome match the study objectives.

The proposed study design is a quantitative study. The researcher hopes to determine medical students' learning needs and preferences in the area of Culinary Medicine with a view to informing development of a culinary medicine course at a London Medical School. The researcher aims to collect data using a questionnaire (see Appendix 4).

The questionnaire will be in electronic format (Google Forms) and a weblink to the questionnaire will be distributed via email to current students of UCL Medical School by The Royal Free, University College and Middlesex Medical Students' Association (RUMS MSA) on behalf of the researcher and student societies, such as UCL Medical Society, the RUMS review and RUMS Islamic Society. The researcher will also share the weblink to the questionnaire amongst colleagues and friends via university email (see Appendix 5).

The questionnaire will take approximately 15 minutes to complete.

Questionnaire studies enable data from a large number of study participants to be gathered without taking up too much of participants' time.

The questionnaire consists of a combination of questions: Likert-type scale (Sullivan *et al.* 2013), free-text, proportions and ranking.

The topics and the majority of the questions included in the questionnaire were the researcher's and chief investigator's own questions that they would like to be answered. The questions under the sub-heading 'Communication and Understanding' were developed from the GMC's 'Outcomes for Graduates 2018' document (General Medical Council, 2018). All questions were written prior to the conducting a literature review.

The only piece of demographic data that will be collected is the year of medical school training. The researcher feels that this information is important because the data may reveal a correlation between the year of study and students' understanding of Culinary Medicine and their preferences and expectations of a Culinary Medicine course in their medical programme. The course may be adjusted according to the education needs and preferences of different year groups.

For peer review only

Questionnaires will be anonymised to encourage participants to answer the questions honestly, with the hope of increasing the validity of the data collected.

Study Population

The study population is undergraduate medical students who are enrolled at UCL Medical School for the academic year 2018-19.

The researcher contacted the UCL Student Data Analyst to obtain the study population size, which is 1669.

A sample size calculation (https://www.surveymonkey.co.uk/mp/sample-size-calculator/) was done using total population (1669) of UCL medical students (undergraduates) in the academic year 2018-19. 313 responses are required for the results to be accurate at the 95% confidence level, with a 5% margin of error.

The researcher aims to receive approximately 330 responses to the questionnaire.

Duration of Enrolment and Follow-up

Participants will be recruited from November – December 2018.

There will be no participant follow-up. However, if participants wish, they may contact the researcher via email (an email address will be provided on the recruitment flyer) to ask for a summary of the study findings.

Methods Used to Determine the Appropriate Sample Size

A sample size calculation (https://www.surveymonkey.co.uk/mp/sample-size-calculator/) was done using total population (1669) of UCL medical students in the academic year 2018-19. 313 responses are required for the results to be accurate at the 95% confidence level, with a 5% margin of error.

Analysis Plan

The researcher will seek advice from their chief investigator, project supervisor and a statistician within the UCL Department of Primary care and Population Health when analysing the data collected from the quantitative part of the questionnaire.

The exact statistical analysis will depend on the distribution of the variables in the final sample. The researcher will use SPSS statistical software. The researcher will form a statistical analysis plan with the UCL statistician.

The researcher also intends on doing a thematic analysis of the free-text responses in the questionnaire. The method of thematic analysis is described by Braun and Clarke (2006) as follows:

1. Familiarisation with the raw data.
   - The researcher will read and re-read the free-text responses.
2. Generating initial codes.
   - Codes will be both informed by the questions and derived from the data (Stuckey, 2015).
   - Codes will be initially written on hard copies of transcripts and then input onto NVivo (11.4.3) software to allow for easier organisation of the codes.

For peer review only - http://bmjopen.bmj.com/site/about/guidelines.xhtml

- The researcher will code the full data set. The researcher will ask a project supervisor with expertise in qualitative methods to code a subset of free-text responses to allow for different perspectives on the emerging data and themes to be gathered.
- A constant comparative method of analysis will be used: emerging codes will be compared against the existing list of codes and refined. Previous the free-text responses will be re-coded using the revised list. This process will continue until no new codes emerge, ensuring thorough coding of all the free-text responses and more robust analysis (Hewitt-Taylor, 2001).
- The researcher will compare coded sections within, and between, the free-text responses to check the consistency of coding (Strauss and Corbin, 1990).

3. Searching for themes.
    - Codes that share common elements will be grouped together under a sub-theme. Sub-themes will be grouped together under a core theme (Strauss and Corbin, 1990).
4. Reviewing themes.
    - The researcher will examine if the data within each theme is coherent.
    - The researcher will assess the validity of each theme in relation to the data set and ensured that there is no overlap between themes.
    - The researcher will draw a thematic map to illustrate how the themes are related to one another.
5. Defining and naming themes.
    - The researcher, chief investigator and project supervisor will offered their interpretations of the data.
6. Produce a report.
    - The study will be disseminated.

This study is prone to response bias. Therefore, the questionnaire will consist of non-leading, short and clearly-written questions. The questions were independently reviewed by the chief investigator and project supervisor.

This study is prone to non-response bias. Therefore, the researcher will advertise the questionnaire via a wide range of university student societies and encourage these societies to advertise on multiple social media platforms, with the hope of increasing the questionnaire response rate and recruiting a wide range of students.

5 STUDY SCHEDULE

Study Plan

Action	Time for Completion
Protocol write up	July 2018 – October 2018
Ethical approval	November 2018

For peer review only - http://bmjopen.bmj.com/site/about/guidelines.xhtml

Seek permission from the Head of Divisional Research and Director of the Research Department of Medical Education (RDME) of UCL Medical School to ask for to carry out research which will involve UCL medical students being the study participants	November 2018
Identify and recruit participants	November - December 2018
Data analysis	December 2018 – February 2019
Dissemination of results	December 2018 – February 2019

Data Collection Process

The researcher will pilot the questionnaire with three colleagues (medical students at UCL Medical School) before data collection begins to validate the readability and acceptability of the questions and to increase the reliability of the questionnaire. If any changes are made to the questionnaire, an updated version of the questionnaire will be created and used for the study.

Prior to completing the questionnaire, participants must read and understand the PIS and the consent form and complete the consent form.

The questionnaire will be in electronic format (Google Forms) that should take participants approximately 15 minutes to complete.

End of Study

The end of the study will be when the number of questionnaire responses reaches the sample size figure calculated according to the total population size, the data has been analysed and the results have been disseminated.

6 CONSENT

Informed consent will be obtained from study participants prior to the start of the questionnaire.

The first page of the online questionnaire will be the participant information sheet (PIS; see Appendix 6) and the consent form (see Appendix 7), which study participants must read and complete before they can start in the questionnaire.

Should study participants have any questions or concerns to raise with the researcher, the researcher will be contactable via e-mail or telephone and these contact details will be written in the PIS. With this information, the participants may decide if they would like to take part in the study.

For peer review only - http://bmjopen.bmj.com/site/about/guidelines.xhtml

7 ELIGIBILITY CRITERIA

7.1 Inclusion Criteria

All students who are currently studying Medicine at UCL (academic year 2018-19) are eligible to be included in this study.

7.2 Exclusion Criteria

Any persons who is not currently studying Medicine at UCL will be excluded from this study.

8 RECRUITMENT

Participants will be recruited from November – December 2018.

Participant recruitment will cease when the number of questionnaire responses reaches the sample size figure calculated according to the total population size.

Recruitment Method

Participants will be recruited from November – December 2018 from UCL Medical School only.

The researcher will ask RUMS MSA to distribute a weblink to the questionnaire to current students of UCL Medical School on behalf of the researcher. The researcher will also ask student societies, such as UCL Medical Society, the RUMS review and RUMS Islamic Society, to include a weblink to the questionnaire in their electronic newsletters. The researcher will also share the weblink to the questionnaire amongst colleagues and friends via university email (see Appendix 5).

There will be no participant follow-up. However, if participants wish, they may contact the researcher via email (an email address will be provided on the recruitment flyer) to ask for a summary of the study findings.

9 STATISTICAL METHODS

A sample size calculator (https://www.surveymonkey.co.uk/mp/sample-size-calculator/) was used to calculate a sample size of 313 for a total population of 1669 undergraduate medical students (academic year 2018-19) at the 95% confidence level, with a 5% margin of error. Therefore, the researcher aims to receive approximately 330 responses to the questionnaire.

The researcher will seek advice from their chief investigator, project supervisor and a statistician within the UCL Department of Primary care and Population Health when analysing the data collected from the quantitative part of the questionnaire.

The researcher will form an analysis plan with a UCL statistician based at the UCL department of Primary Care and Population Health.

For peer review only - http://bmjopen.bmj.com/site/about/guidelines.xhtml

10 PATIENT AND PUBLIC INVOLVEMENT (PPI)

Participant Involvement

Two UCL medical students (one in fifth-year and one in third-year) will be recruited by the researcher through convenience sampling.

PPI will be asked to provide feedback on the quality and quantity of the questionnaire questions, for example if there is any ambiguity, and on the recruitment flyer. PPI will also be involved in the data analysis to check if the study findings are valid so will therefore have access to the S: drive in the UCL department of Primary Care and Population Health at the Royal Free Hospital Campus that will contain questionnaire responses and participant identification numbers.

PPI will be informed that they cannot participate in the study.

PPI will be reimbursed £50 each for their time.

No other members of the public or study participants will be involved in the planning and design of the study. Study participants will only be used as a source to collect data from.

Participant Withdrawal

During the recruitment and data collection processes, it is important that the researcher is transparent. It will be clearly stated in the PIS (see Appendix 6) that participants are not obliged to take part in this study and can withdraw from the study at any moment in time, without having to give a reason for their withdrawal.

Management of the Research

The members of the Medical School community who will have input in this study will be researcher, chief investigator and project supervisor(s), who are part of the UCL Department of Primary Care and Population Health at the Royal Free Hospital Campus.

Dissemination of Findings

The results of this study will be disseminated and a summary of the study findings will be provided for UCL Medical School. If participants wish, they may contact the researcher via email (an email address will be provided on the recruitment flyer) to ask for a summary of the study findings. Participants will be informed that the researcher may be contacted on the PIS and at the end of the questionnaire.

11 FUNDING AND SUPPLY OF EQUIPMENT

The study funding has been reviewed by the UCL/UCLH Research Office, and deemed sufficient to cover the requirements of the study. NHS costs will be supported via UCLH and/or the Local Clinical Research Network.

Equipment or Resources Required	Estimated Cost	Funded by

For peer review only - http://bmjopen.bmj.com/site/about/guidelines.xhtml

Reflective journal	Free	UCL Department of Primary Care and Population Health
Printing free-text responses	5p per A4 black and white page	UCL Department of Primary Care and Population Health
Data analysis software (SPSS)	Free	UCL Department of Primary Care and Population Health
Patient and Participant Involvement (PPI)	£50 each	National Institute for Health Research (NIHR) School for Primary Care Research (SPCR)
UCL Statistician based at the UCL department of Primary Care and Population Health	UCL Department of Primary Care and Population Health is already contributing Seedcorn funding to the statistician's salary	UCL Department of Primary Care and Population Health
Printing final report	5p per A4 black and white page	UCL Department of Primary Care and Population Health

12 DATA HANDLING AND MANAGEMENT

There is a low risk of breach of confidentiality. This is because the only data that will be collected are the anonymised questionnaire responses and the year group of study participants. In keeping with UCL data protection regulations, year group is not considered personal data as data subjects cannot be identified either directly or indirectly by reference to their year group. The data will be collected electronically using Google Forms. No identifying data or pseudonymized data will be collected. Each response will be given an identification number but there will be no student identifiable data. Therefore, it will not be possible to trace the identification number and the response back to individual students.

The researcher will ask RUMS MSA to distribute a weblink to the anonymous questionnaire to current students of UCL Medical School on behalf of the researcher. The researcher will also ask student societies, such as UCL Medical Society, the RUMS review and RUMS Islamic Society, to include a weblink to the anonymous questionnaire in their electronic newsletters. Therefore, the researcher will have no access to email addresses of the potential study participants.

The researcher will input questionnaire responses onto an Excel spreadsheet. The researcher will save the questionnaire responses and identification numbers in a password-protected file on the S:drive in the UCL department of Primary Care and Population Health at the Royal Free Hospital Campus.

For peer review only - http://bmjopen.bmj.com/site/about/guidelines.xhtml

Anonymous free-text questionnaire responses will be printed out for the analysis process. The physical copies of the free-text questionnaire responses will be stored in a locked cabinet at the UCL department of Primary Care and Population Health at the Royal Free Hospital Campus.

Only the principal researcher (Jessica Xie), project supervisor (Shoba Poduval), chief investigator (Sophie Park), the PPI and a statistician based at the UCL department of Primary Care and Population Health will have access to the password-protected file on the S:drive and therefore the data.

All data will be stored in compliance with the Data Protection Act.

Data protection registration number: Z6364106/2018/11/17.

UCL department of Primary Care and Population Health policy: anonymised data will be stored within the department for 20 years.

13 MATERIAL/SAMPLE STORAGE

Not applicable to this study.

14 PEER AND REGULATORY REVIEW

The study will be peer reviewed by 2 academic staff from the Department of Primary Care and Population Health at UCL Medical School and PPI (two UCL medical students (one in fifth-year and one in third-year)).

15 ASSESMENT AND MANAGEMENT OF RISK

This is a low risk study. There are no risks to the participants in completing the questionnaire. Prior to starting the questionnaire, participants will be reminded that they may withdraw from the study at any moment in time, without having to provide a reason for withdrawal.

16 RECORDING AND REPORTING OF EVENTS AND INCIDENTS

Not necessary at present.

17 MONITORING AND AUDITING

Not necessary at present.

18 TRAINING

Not necessary at present.

19 INTELLECTUAL PROPERTY

Not necessary at present.

For peer review only - http://bmjopen.bmj.com/site/about/guidelines.xhtml

20 INDEMNITY ARRANGEMENTS

Covered by the UCL indemnity policy.

21 ARCHIVING

Not necessary at present.

22 PUBLICATION AND DISSEMINATION POLICY

The results of this project will be disseminated.

UCL Medical School will receive a report of the study findings. If participants wish, they may contact the researcher via email (an email address will be provided on the recruitment flyer) to ask for a summary of the study findings. Participants will be informed that the researcher may be contacted on the PIS and at the end of the questionnaire.

If appropriate, the research will be submitted for publication of the findings in a peer-reviewed journal and/or present the findings at a conference.

23 REFERENCES

Bibliography

Adam, O., Beringer, C., Kless, T., Lemmen, C., Adam, A., Wiseman, M., Adam, P., Klimmek, R., Forth, W. (2003). Anti-inflammatory effects of a low arachidonic acid diet and fish oil in patients with rheumatoid arthritis. *Rheumatology international*, 23(1), pp.27–36.

Bendici, Ray (2018). Colleges serve up culinary medicine courses. University Business. Available from: https://www.universitybusiness.com/article/colleges-serve-culinary-medicine-courses [Accessed 11th August 2018].

Braun, V. & Clarke, V. (2006). Using thematic analysis in psychology. Qualitative Research in Psychology [Online], 3(2), pp.77-101.

The British Broadcasting Corporation (BBC) (2010). Nutrition training for doctors ‘must be improved’. British Broadcasting Corporation (BBC) Health. Available from: http://news.bbc.co.uk/1/hi/health/8572874.stm [Accessed 9th August 2018].

Buck, D. (2018). Second’s out, round two: Is the government’s latest childhood obesity plan a knockout? The King’s Fund. Available from: https://www.kingsfund.org.uk/blog/2018/06/second-government-childhood-obesity-plan [Accessed 13th September 2018].

Campbell, D. (2016). Doctors ‘know too little about nutrition and exercise’. The Guardian. Available from: https://www.theguardian.com/society/2016/oct/19/doctors-know-too-little-about-effects-of-nutrition-and-exercise [Accessed 30th July 2018].

For peer review only - http://bmjopen.bmj.com/site/about/guidelines.xhtml

Coles, L. (eds) (2013). *Clinical Nutrition: The Interface Between Metabolism, Diet, and Disease*. CRC Press.

Conroy, M. B., Delichatsios, H. K., Hafler, J. P. and Rigotti, N. A. (2004). Impact of a preventive medicine and nutrition curriculum for medical students. American journal of preventive medicine, 27(1), pp.77–80.

Cooke, N. K., Ash, S. L. and Goodell, L. S. (2017). Medical students' perceived educational needs to prevent and treat childhood obesity. Education for Health, 30(2), pp.156-62.

Crawford, A. L., and Aspry, K. E. (2016). Teaching doctors-in-training about nutrition: Where are we going in 2016?. *Rhode Island Medical Journal*, *99*(3), pp.23-25.

Dillon, S. (2018). We learn nothing about nutrition, claim medical students. British Broadcasting Corporation (BBC) Health. Available from: https://www.bbc.co.uk/news/health-43504125 [Accessed 30th July 2018].

Engel, S. S., Crandall, J., Basch, C. E., Zybert, P. and Wylie-Rosett, J. (1997). Computer-assisted diabetes nutrition education increases knowledge and self-efficacy of medical students. The Diabetes educator, 23(5), pp.545–549.

Ferreria, S. (2018). The Art of Cooking for Health: Culinary Medicine. The American Society for Nutrition. Available from: https://nutrition.org/the-art-of-cooking-for-health-culinary-medicine/ [Accessed 13th September 2018].

General Medical Council (GMC) (2018). Outcomes for graduates 2018. General Medical Council. Available from: https://www.gmc-uk.org/-/media/documents/dc11326-outcomes-for-graduates-2018_pdf-75040796.pdf [Accessed 30th July 2018].

Grewal, J. (2016). Could cooking change the way we approach medicine? Public Radio International. Available from: https://www.pri.org/stories/2016-02-10/could-cooking-change-way-we-approach-medicine [Accessed 13th September 2018].

Hargrove, E. J., Berryman, D. E. and Yoder, J. M., Beverly, E. A. (2017). Assessment of Nutrition Knowledge and Attitudes in Preclinical Osteopathic Medical Students. The Journal of the American Osteopathic Association, 117(10), pp.622–633.

Hewitt-Taylor, J. (2001). Use of constant comparative analysis in qualitative research. Nursing standard (Royal College of Nursing (Great Britain) : 1987), 15(42), pp.39–42.

Jacka, F. N., O'Neil, A., Opie, R., Itsiopoulos, C., Cotton, S., Mohebbi, M., Castle, D., Dash, S., Mihalopoulos, C., Chatterton, M. L., Brazionis, L., Dean, O. M., Hodge, A. M. and Berk, M. (2017). A randomised controlled trial of dietary improvement for adults with major depression (the `SMILES' trial). *BMC Medicine*, 15(1), p.23.

La Puma, J. and Marx, R. (2008). ChefMD's Big Book of Culinary Medicine: A Food Lover's Road Map to Losing Weight, Preventing Disease, and Getting Really Healthy. New York, The Crowne Publishing Group.

For peer review only - http://bmjopen.bmj.com/site/about/guidelines.xhtml

Mlodinow, S. G. and Barrett-Connor, E. (1989). Physicians' and medical students' knowledge of nutrition. *Academic medicine : journal of the Association of American Medical Colleges*, 64(2), pp.105—106.

Moorhouse, J. (2018). Nutritank – Why is nutrition in medical education so important? Food and Behaviour Research. Available from: https://www.fabresearch.org/viewItem.php?id=11464 [Accessed 11th August 2018].

Morrish, V. (2018). Are you eating your medicine? British Society of Lifestyle Medicine. Available from https://bslm.org.uk/are-you-eating-your-medicine/ [Accessed 11th August 2018].

The Organisation for Economic Co-operation and Development (2017). Health at a Glance 2017: OECD Indicators, OECD Publishing, Paris. Available from: https://read.oecd-ilibrary.org/social-issues-migration-health/health-at-a-glance-2017_health_glance-2017-en#page4 [Accessed 9th August 2018].

Leith, P. (2018). How culinary medicine could solve many of the NHS's problems. The Telegraph. Available from: https://www.telegraph.co.uk/health-fitness/nutrition/diet/culinary-medicine-need-unlock-secrets-medical-kitchen-cabinet/ [Accessed 11th August 2018].

Perlstein, R., McCoombe, S., Shaw, C. and Nowson, C. (2016). Medical students' perceptions regarding the importance of nutritional knowledge and their confidence in providing competent nutrition practice. Public Health, 140, pp.27-34.

Sarris, J., Logan, A., Akbaraly, T. N., Amminger, G. P., Balanzá-Martínez, V., Freeman, M. P., Hibbeln, J., Matsuoka, Y., Mischoulon, D., Mizoue, T., Nanri, A., Nishi, D., Ramsey, D., Rucklidge, J. J., Sanchez-Villegas, A., Scholey, A., Su, K-P. and Jacka, F. N. (2015). Nutritional medicine as mainstream in psychiatry. *The Lancet Psychiatry*, 2(3), pp.271–274.

Schlair, S., Hanley, K., Gillespie, C., Disney, L., Kalet, A., Darby, P. C., Frank, E., Spencer, E., Harris, J. and Jay, M. (2012). How Medical Students' Behaviors and Attitudes affect the Impact of a Brief Curriculum on Nutrition Counseling. *Journal of Nutrition Education and Behavior*, 44(6), pp.653–657.

Strauss, A., & Corbin, J. M. (1990). Basics of qualitative research: Grounded theory procedures and techniques. Newbury Park, CA, US: Sage Publications, Inc.

Stuckey, H. (2015). The second step in data analysis: Coding qualitative research data . Journal of Social Health and Diabetes, 3(1), pp.7–10.

Sullivan, G. M. and Artino, A. R. (2013). Analyzing and Interpreting Data From Likert-Type Scales. Journal of Graduate Medical Education, 5(4), pp. 541–542.

Tappenden, K. A., Quatrara, B., Parkhurst, M. L., Malone, A. M., Fanjiang, G. and Ziegler, T R. (2013). Critical Role of Nutrition in Improving Quality of Care: An Interdisciplinary Call to Action to Address Adult Hospital Malnutrition. *Journal of the Academy of Nutrition and Dietetics*, 113(9), pp.1219–1237.

For peer review only - http://bmjopen.bmj.com/site/about/guidelines.xhtml

Vettel, S. (2018). The Rold of Medical Nutrition Therapy in Addressing Food Insecurity and Disease Prevention. Emitro Health. Available from: https://emtirohealth.org/knowledge/2018/1/7/the-role-of-medical-nutrition-therapy-in-addressing-food-insecurity-and-disease-prevention [Accessed 13th September 2018]

Walsh, C. O. Walsh, Carolyn O., Ziniel, S. I., Delichatsios, H. K. and Ludwig, D. S. (2011). Nutrition attitudes and knowledge in medical students after completion of an integrated nutrition curriculum compared to a dedicated nutrition curriculum: a quasi-experimental study. *BMC Medical Education*, 11(58).

Ward, N. (2003). Nutrition support to patients undergoing gastrointestinal surgery. *Nutrition Journal*, *2*(18).

Womersley, K. and Ripullone, K. (2017). Medical schools should be prioritising nutrition and lifestyle education. The British Medical Journal (BMJ) Opinion. Available from: https://blogs.bmj.com/bmj/2017/09/07/medical-schools-should-be-prioritising-nutrition-and-lifestyle-education/ [Accessed 30th July 2018].

For peer review only - http://bmjopen.bmj.com/site/about/guidelines.xhtml

24 APPENDICES

Appendix 1

For peer review only

For peer review only - http://bmjopen.bmj.com/site/about/guidelines.xhtml

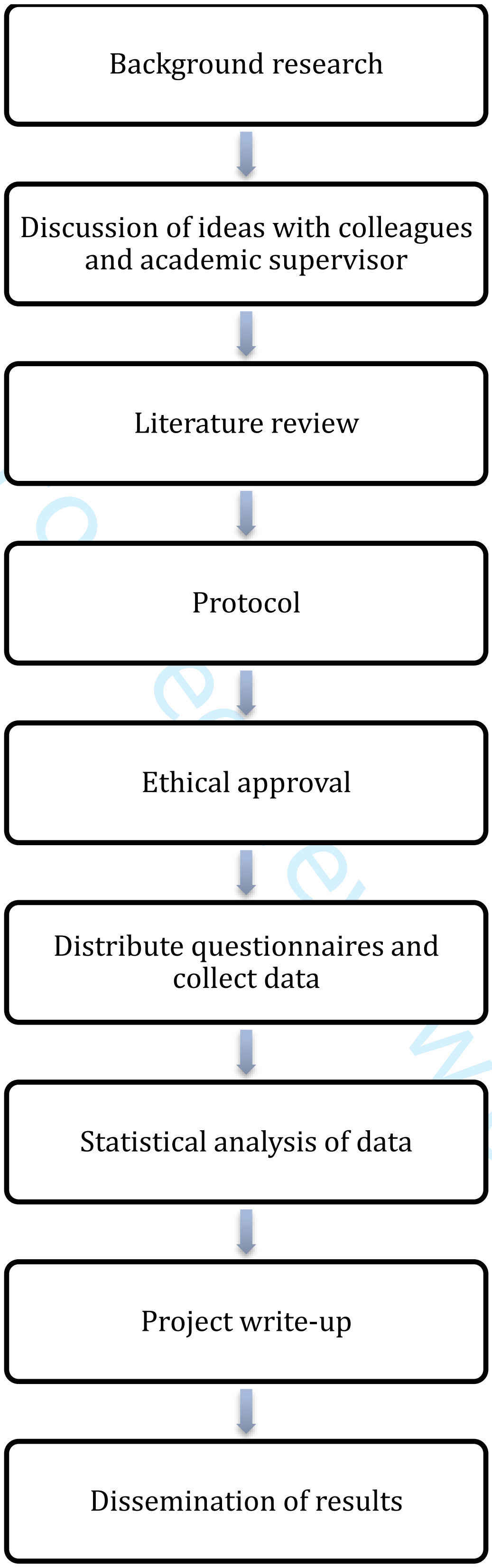

For peer review only - http://bmjopen.bmj.com/site/about/guidelines.xhtml

Appendix 2 tSearch Strategy for Literature Review

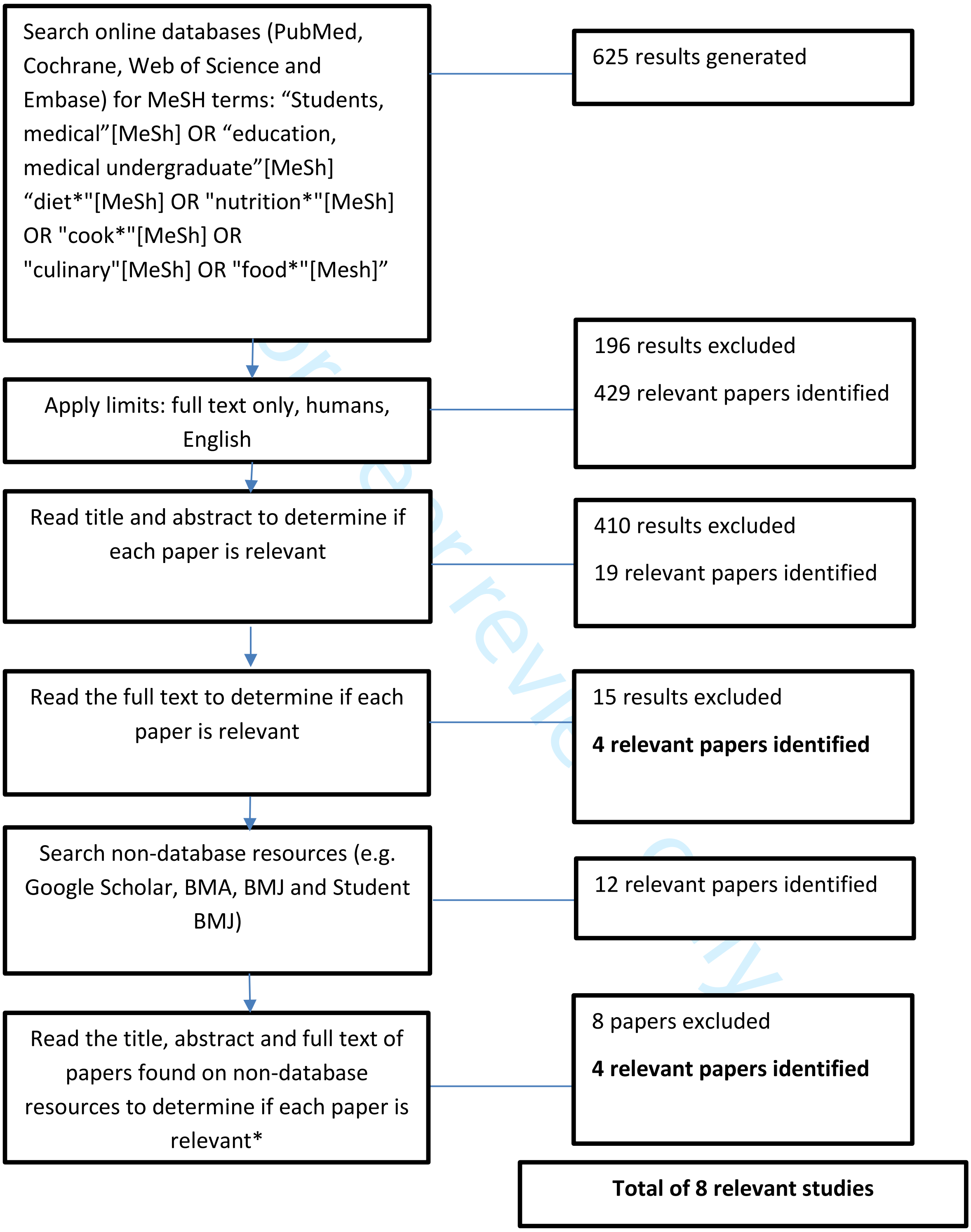

For peer review only - http://bmjopen.bmj.com/site/about/guidelines.xhtml

* When viewing the full text of each paper, also read the title and abstracts of papers in the sections "We recommend" or "Similar articles" or "Other content recommended for you"

For peer review only

For peer review only - http://bmjopen.bmj.com/site/about/guidelines.xhtml

For peer review only

For peer review only - http://bmjopen.bmj.com/site/about/guidelines.xhtml

Appendix 3 Literature Review: Summary Table of Literature

Author, Date, Country	Title of Study	Objective	Method	Strengths	Limitations	Results	Conclusions
Schlair, S., Hanley, K., Gillespie, C., Disney, L., Kalet, A., Darby, P. C., Frank, E., Spencer, E., Harris, J. and Jay, M.<br><br>2012<br><br>USA	How Medical Students' Behaviors and Attitudes affect the Impact of a Brief Curriculum on Nutrition Counselling	To evaluate a nutrition curriculum and explore the influence of medical students' own nutrition practices on its impact.	Quantitative study: 19-item a survey to measure self-reported nutrition-related competency, attitude, counselling confidence, ability to assess diet and nutrition knowledge measured using 4-point Likert scales	• Questions were selected from previously validated surveys<br>• No individual demographic information was collected to preserve individuals' anonymity<br>• Preserving participants' anonymity may have encouraged participants to answer the questions honestly, increasing the validity of the results<br>• A statistical test (Mann-Whitney U) was used during data analysis<br>• The response rates were high: 69% and 75% for responses to the pre-curriculum and post-curriculum surveys respectively, suggesting that the results are valid<br>• Most P-values were less than 0.05, suggesting that the data is statistically significant and that the results are precise	• A small sample size (111 pre-curriculum and 121 post-curriculum) students from a single medical school suggests that the study sample may be unrepresentative of the medical student population and the results may not be generalisable. The credibility of the study may be limited<br>• The sample consisted of first-year medical students only, who may have had limited knowledge of nutrition and limited pre-clinical exposure<br>• Given that participants were aware that they had just completed a nutrition course, there was a potential for social desirability, in addition to other biases<br>• The curriculum was delivered for one day	• There is an overall pre-post difference in dietary assessment ability (2.65 vs 3.05, P<.001) and counselling confidence (1.86 vs. 2.22, P<0.001)<br>• At post-test, students reported being more knowledgeable and confident in all areas<br>• Students' own attitudes and behaviours are important determinants of dietary counselling: students with greater nutrition self-efficacy and	A nutrition curriculum for medical students improves students' nutrition counselling-related confidence, knowledge, and skills even when controlling for personal nutrition-related behaviours

For peer review only - http://bmjopen.bmj.com/site/about/guidelines.xhtml

					without reinforcement so the long-term impact(s) of the curriculum is unknown<br>• No direct patient outcomes were measured post-curriculum<br>• No power calculation<br>• No critical examination of the possible impact of the researchers' biases on the method or results	greater fruit and vegetable intake are more likely to have higher nutrition counselling confidence and ability to assess diet and dietary knowledge	
Mlodinow, S. G. and Barrett-Connor, E.<br><br>1989<br><br>USA	Physicians' and medical students' knowledge of nutrition.	To assess the knowledge of nutrition of family practitioners, general internists and first- and second-year medical students before they receive	Quantitative study: a 69-item questionnaire was mailed to study participants	• A total of 461 physicians were sent questionnaires. Physicians were chosen at random to take part in the study and the sample size is fairly large, implying that they study sample may be representative of family practitioners and general internists populations<br>• Participants' demographics (physicians' speciality, age and gender) were collected and analysed for differences between individual physicians and between physicians and medical students, reducing the effect of confounding factors on the results<br>• The high response rate for students (100%) suggests that the results may be valid for this group	• A very small sample size (24) of medical students suggests that the study sample may be unrepresentative of the medical student population and the results may not be generalisable. The credibility of the study may be limited<br>• The low response rate for physicians (40%) suggests that the results may be invalid for this group<br>• No power calculation<br>• No critical examination of the possible impact of the researchers' biases on the method or results	• Physicians answered 69.2% of the questions correctly, answering better on well-researched topics (for example, coronary heart disease), compared to less-well-researched topics (for example, iron absorption)<br>• Medical students answered 62.5% of the questions correctly<br>• A significant negative correlation was	Physicians and medical students' knowledge on nutrition varies between the two groups. Each group is knowledgeable in a different area

For peer review only - http://bmjopen.bmj.com/site/about/guidelines.xhtml

		medical school instruction in clinical nutrition				found between the number of years since graduation form medical school and respondents' knowledge about nutrition	
Walsh, C. O. Walsh, Carolyn O., Ziniel, S. I., Delichatsios, H. K. and Ludwig, D. S.<br><br>2011<br><br>USA	Nutrition attitudes and knowledge in medical students after receiving nutrition training as part of an integrated nutrition curriculum compared to a dedicate	To assess the nutrition knowledge and attitudes of medical students following completion of a nutrition curriculum	A quantitative study: a quasi-experimental survey with two groups (Preventive Medicine and Nutrition (PMN) – a dedicated nutrition course - and Introduction to Clinical Nutrition (ICN) – an integrated curriculum) completed the 60-item	• The survey was available online for 3 weeks and weekly invitation emails were sent to participants, which may have increased the response rate to the surveys and may therefore have increased the validity of the results<br>• Preserving participants' anonymity may have encouraged participants to answer the questions honestly, increasing the validity of the results<br>• Power calculation for response rate of 50%: 0.8, with alpha level of 0.05<br>• 5 questions were included as validation items stated negatively rather than positively to control for possible response bias	• A small sample size (266) students from a single medical school suggests that the study sample may be unrepresentative of the medical student population and the results may not be generalisable. The credibility of the study may be limited<br>• The low response rates (50.4% and 42.2% for PMN and ICN students respectively) suggest that the results may be invalid	• There was no difference between the two groups in attitude scores (P = 0.43) or knowledge scores (P = 0.63)<br>• Students on the integrated curriculum were less satisfied with both the quantity (P<0.0001) and quality (P = 0.008) and were more likely to have completed optional online nutrition training modules (P = 0.0089)	Medical students prefer learning about nutrition on a dedicated nutrition course rather than an integrated curriculum

	d nutrition curriculum: a quasi-experimental study		survey two years after completing the nutrition course. Most questions were rated on a five-point Likert scale was used. A few questions were open-ended				
Engel, S. S., Crandall, J., Basch, C. E., Zybert, P. and Wylie-Rosett, J.<br><br>1997	Computer-assisted diabetes nutrition education increases knowledge and self-efficacy of	To develop and evaluate a computer programme targeting basic competencies for prescribing a	Quantitative: a 10-item multiple-choice knowledge test was used to evaluate participants' knowledge of diabetes nutrition prior to and after	• All multiple choice questions were reviewed by an expert group of dieticians specializing in diabetes, ensuring validity of the questions<br>• The computer programme used had been evaluated for content validity by an expert panel of dieticians and endocrinologists with a specialty in diabetes<br>• Feedback from a pilot test (conducted with medical residents, medical students, nurses and dieticians) was incorporated into the revised version of the computer programme to improve the reliability of the questionnaire	• A very small sample size (41) students from a single medical school suggests that the study sample may be unrepresentative of the medical student population and the results may not be generalisable. The credibility of the study may be limited<br>• There was no control or standard teaching method to allow evaluation of the efficacy of CAI as a teaching method	• The mean knowledge score amongst medical students prior to CAI was 3.7 (standard deviation = 1.7), which was similar to the score among the general care nurses with no diabetes-related nutrition training<br>• The mean knowledge score	CAI is a practical and efficient method of teaching medical students about nutrition

For peer review only - http://bmjopen.bmj.com/site/about/guidelines.xhtml

USA	medical students	diabetic diet	completing a computer-assisted instruction (CAI) on diabetes nutrition. An 8-item self-efficacy scale was used to measure participants' perceived self-efficacy for making dietary prescriptions	• All participants who entered the study completed it	• No critical examination of the possible impact of the researchers' biases on the method or results	among medical students after completing CAI was 6.4 (standard deviation = 2), which is similar to the mean score among dieticians (post-CAI mean for dieticians = 7.8)<br>• Significant increases in knowledge occurred for seven out of ten knowledge items<br>• The mean self-efficacy scores among medical students increased from 13.54 (standard deviation = 5.1) to 30.24 (standard deviation = 5.9) for all eight items	
Cooke, N. K., Ash, S. L. and	Medical students' perceived	To explore US medical	Qualitative: semi-structured phone	• Third- and fourth-year medical students were recruited from a large sample (25) of different US medical schools in 16 different states. Participants' ages ranged	• The sample size was small (78) and the study subjects over-represent the percentage of students pursuing careers in	• Five major themes that arose:<br>• Perceived barriers to	US medical schools need to provide

For peer review only - http://bmjopen.bmj.com/site/about/guidelines.xhtml

Goodell, L. S.<br><br>2017<br><br>USA	educational needs to prevent and treat childhood obesity	students' understanding of childhood obesity, specifically barriers to childhood obesity prevention and treatment and students' perceived educational deficits.	interviews during which students were asked to describe the aetiology of childhood obesity and reflect on where they acquire knowledge of the aetiology and what additional resources they would need to treat obesity in children	from 23 to 44 years old and they came from a range of different educational backgrounds. This increases the likelihood that the study sample was representative of the US medical student population and the results may be generalisable. This may have increased credibility of the study<br>• Participant recruitment was initially through listervs<br>• Participants were also recruited by snowball sampling, which would have enabled the researchers to contact potential study participants who would otherwise not have been easily-accessible, but it may have resulted in oversampling of a select group of similar medical students<br>• Participant recruitment ceased when no new themes emerged, implying that data saturation had been reached, implying that sufficient data has been obtained, strengthening study credibility<br>• Semi-structured interviews allowed deep exploration of topics to reveal the complexity of medical students' experiences and opinions<br>• The interviews were audio-recorded and all bar two were transcribed verbatim. Transcribing	paediatrics and family medicine. Also, students were asked to volunteer to participate in the study, which may mean that the study participants may have been more interested in, and therefore may be more knowledgeable on, the subject of nutrition/obesity than a typical US medical student. Therefore, this decreases the likelihood that the study sample is representative of the US medical student population<br>• Interviews over the phone would not have allowed the interviewer to observe, and appropriately respond to, interviewees' verbal narrative. Face-to-face are often more in-depth and yield richer data<br>• Two interviews were not transcribed verbatim due to technical faults with the audio files. Therefore, not all data was analysed<br>• The interview questions may have been shared	medical students' childhood obesity training<br>1. Medical student-centred<br>2. Patient-centred<br>3. Health-care system-centred<br>• Perceived needs associated with medical students' childhood obesity training<br>4. Knowledge<br>5. Counselling skills<br>• Students requested more applicable nutrition information and counselling skills relevant to preventing and treating childhood obesity	students with childhood obesity-specific and general nutrition education

For peer review only - http://bmjopen.bmj.com/site/about/guidelines.xhtml

				verbatim ensures that all aspects of the interviewees' verbal narrative are recorded<br>• Multiple (four) coders individually coded transcripts. Investigator triangulation allowed for different perspectives on the emerging data and themes to be gathered<br>• The interviewer used non-judgemental and unbiased language to increase trustworthiness of the data<br>• Participant validation was conducted to increase the trustworthiness of the data	between the study participants, which would have produced inorganic answers to the interview questions and this may decrease the validity of responses<br>• No critical examination of the possible impact of the researchers' biases on the method or results		
Conroy, M. B., Delichatsios, H. K., Hafler, J. P. and Rigotti, N. A.<br><br>2004<br><br>USA	Impact of a Preventive Medicine and Nutrition Curriculum for Medical Students	To evaluate the impact of an innovative preventive medicine and nutrition (PMN) course on	Quantitative study: second-year medical students at Harvard Medical School completed a 43-item written survey before and after completing	• The high response rate for students (98% and 86% for the baseline and follow-up surveys respectively) suggests that the results may be valid for this group<br>• Students were only identified by their unique confidential code, which was unknown to investigators. Therefore, this reduced observer bias and increased the credibility of the study<br>• A comparison group was included to ensure that changes in students' attitudes and behaviours over time were not attributable to temporal changes. This increases the validity of the results	• A small sample size (137) students from a single year group (second-year) at a single medical school (Harvard University) suggests that the study sample may be unrepresentative of the medical student population and the results may not be generalisable. The credibility of the study may be limited. However, the authors acknowledged this limitation and explained that "the PNM course is required for most HMS students, [therefore]	• Students' confidence in their ability to assess and counsel about diet and exercise significantly improved after the course (all p =0.001).<br>• The course was associated with a decrease in students' self-reported consumption of saturated fat (p	An innovative PMN course improved medical students' confidence in diet and exercise counselling and perceived dietary habits. Improving these

For peer review only - http://bmjopen.bmj.com/site/about/guidelines.xhtml

		medical students' confidence about addressing patients' diet and exercise patterns and on their own health habits	the PMN course		it is not possible to increase the small size of the comparison group or randomly assign students to groups"<br>• Self-reported confidence is an subjective measure of counselling skills. Therefore, this may have increased the risk of measurement bias<br>• No critical examination of the possible impact of the researchers' biases on the method or results	=0.002) and trans fatty acids (*p* =0.001).<br>• Following the course, 72% of students perceived an improvement in their diet and s18% perceived an improvement in exercise habits	mediators of physician counselling in medical students may translate into changes in their practice pattern
Perlstein, R., McCoombe, S., Shaw, C. and Nowson, C.<br><br>2016	Medical students' perceptions regarding the importance of nutritio	To examine the perceived importance, knowledge and confidence in nutritio	Quantitative study: postgraduate medical students in years 1-4 were anonymously surveyed using a 20- to 23-item survey to	• Follow-up reminder emails were sent to students requesting them to complete the survey to increase the response rate<br>• No individual demographic information was collected to preserve individuals' anonymity<br>• Preserving participants' anonymity may have encouraged participants to answer the questions honestly,	• A small sample size (131 first- and second- year students and 66 third- and fourth-year students) from a single medical school (Deakin University) completed the questionnaire. This suggests that the study sample may be unrepresentative of the	• Most preclinical students agreed that medical graduates should understand nutritional issues in managing cardiovascular disease (99%),	The study participant recognised the importance of nutrition in disease

For peer review only

Australia	nal knowledge and their confidence in providing competent nutrition practice.	nal management in a sample of Australian medical students undertaking a 4-year postgraduate medical degree	assess students' perceived importance of nutrition, and knowledge and confidence in nutritional management	increasing the validity of the results<br>• The surveys were modified according to the year of medical training, ensuring that students had sufficient knowledge to be able to answer the questions	medical student population and the results may not be generalisable. The credibility of the study may be limited.<br>• The low response rates (46% for first- and second-year students and 24% response rate for third- and fourth-year students suggest that the results may be invalid<br>• Self-reported confidence in knowledge is an subjective measure. Therefore, this may have increased the risk of measurement bias<br>• No critical examination of the possible impact of the researchers' biases on the method or results	type 2 diabetes (93%), coeliac disease (95%), and renal impairment (97%), however students lacked confidence in their knowledge in these areas (range of confidence: 26%e41%)<br>• >80% reported confidence in the dietary management of type 2 diabetes, cardiovascular disease and coeliac disease<br>• >60% indicated they would refer onto nutrition professionals<br>•	
Hargrove, E. J., Berryman, D. E. and	Assessment of Nutritio	To assess the nutritio	Descriptive, cross-sectional study:	• The large sample size (424), obtained from three different university campuses. suggest that the study sample may be	• The quiz was not validated<br>• Not all students completed every question. This may have been because some	• The average score on the nutrition	Most first- and second-year

For peer review only

For peer review only - http://bmjopen.bmj.com/site/about/guidelines.xhtml

Yoder, J. M. and Beverly, E. A.<br><br>2017<br><br>USA	n Knowledge and Attitudes in Preclinical Osteopathic Medical Students	n knowledge of osteopathic medical students and their attitudes regarding the importance of nutrition counselling in their future role as practicing physicians	authors evaluated first- and second-year students' nutrition knowledge and attitudes towards nutrition counselling.<br><br>Quantitative study: participants completed questionnaire to assess their attitude towards nutrition counselling and completed a quiz to test their nutrition knowledge	representative of osteopathic medical students<br>• Students were offered an incentive ($15 gift card) to participate in the study, which would have increased the study sample to produce more data and results that are more representative of the study population<br>• Follow-up reminder emails were sent to students requesting them to complete the survey and quiz to increase the response rates<br>• The questions that formed the quiz were from a reliable nutrition textbook and had been used in a previous study. This ensured quality of the questions<br>• Questions that assessed general nutrition knowledge had been developed specifically for use in this study. This ensured that the questions were specific to the research question, and would yield answers that fulfilled the study's aims and objectives<br>• Informed consent was obtained from participants, ensuring that the study was ethical<br>• To avoid coercion, both the online screen to the survey and the informed consent document	questions were poor quality. Less data was yielded<br>• The study sample was homogenous as participants all belonged to one osteopathic medical school in the USA (Ohio University Heritage College of Osteopathic Medicine (OU-HCOM)). Therefore, the ability to generalise the findings to all osteopathic medical schools is limited<br>• Participating students who volunteered may have been more willing or motivated to answer questions about nutrition compared to those who did not volunteer. Self-reported findings are susceptible to selection bias<br>• No critical examination of the possible impact of the researchers' biases on the method or results	knowledge quiz was 69.5%<br>• Second-year students performed better than first-year students on the knowledge quiz (P<0.001)<br>• 55.6% of participants felt comfortable counselling patients on nutrition recommendations<br>• 11.9% were aware of the current dietary reference intakes	medical students felt comfortable providing nutritional counselling, but most participants lacked knowledge of dietary reference intakes and medical nutrition therapy. Students expressed the importance of providing patient education, promoting overall

For peer review only - http://bmjopen.bmj.com/site/about/guidelines.xhtml

				clarified the that participation was voluntary<br>• Anonymity at the level of the data prevented observation bias			health and wellness and preventin g and treating disease

For peer review only - http://bmjopen.bmj.com/site/about/guidelines.xhtml

For peer review only

For peer review only - http://bmjopen.bmj.com/site/about/guidelines.xhtml

Appendix 4 - Questionnaire

About You

1) Which year of the medical (MBBS) programme are you currently in? *Please select one option*.

1 2 3 4 5 6

2) What is your career intention? *Please select one option that is most accurate at present.*

Academic Medicine	Obstetrics and Gynaecology
Acute Medicine	Ophthalmology
Anaesthesia	Orthopaedics
Cardiology	Paediatrics and Child Health
Dermatology	Palliative Medicine
Emergency Medicine	Pathology
Ears, Nose and Throat	Psychiatry
Endocrinology	Public Health Medicine
Gastroenterology	Radiology
General Medicine	Respiratory Medicine
General Practice	Rheumatology
Geriatric Medicine	Sport and Exercise Medicine
Haematology	Surgery
Histopathology	Tropic Medicine
Infectious Diseases	Urology
Nephrology	A career not in Medicine
Neurology	Other (please specify) ………………………………………………..

Education

3) What do you understand by the term 'Culinary Medicine'?

……..

……..

4) Which of the following, if any, Culinary Medicine teaching have you received so far in the MBBS programme?

Please choose as many statements as relevant.

Problem-based learning from patient cases

For peer review only - http://bmjopen.bmj.com/site/about/guidelines.xhtml

Practical kitchen session

Evidence evaluation

Self-directed learning

Small-group seminars/ tutorials

Lectures

Webinar (internet) version of the lecture or seminar

Other(s) (please specify below)

…….

5) Have you received any extracurricular Culinary Medicine teaching? If so, please describe below, including which organisation(s) ran the session, the topic(s) that were covered, the style of teaching and your opinion of the learning experience.

……

……

…….

…….

Communication and Understanding

Please place a tick for each statement as appropriate.

	Strongly disagree	Disagree	Uncertain	Agree	Strongly agree
6) I am able to facilitate a discussion with patients about diet and/or dietary change					
7) I am able to take dietary history from a patient					
8) I am able to apply principles, methods and knowledge relating to nutrition to medical practice and integrate these into patient care					
9) I am able to provide dietary advice for patients with varying cultural, social and economic needs					

Delivery of an Culinary Medicine Course for Undergraduate UK Medical Students

Please place a tick for each statement as appropriate.

	Strongly disagree	Disagree	Uncertain	Agree	Strongly agree
9) I believe that learning Culinary Medicine is important for my future clinical practice					
10) I am satisfied with the *quality* of Culinary Medicine training that my medical school provides					
11) I am satisfied with the *quantity* of Culinary Medicine training that my medical school provides					

12) Please rank the following teaching methods in order of appropriateness for a Culinary Medicine course (1 = most effective; 7 = least effective). Please note that one or more of the below may be used in combination in teaching.

Problem-based learning from patient cases Rank:
Practical kitchen session Rank:
Evidence evaluation Rank:
Self-directed learning Rank:
Small-group seminars/ tutorials Rank:
Lectures Rank:
Webinar (internet) version of the lecture or seminar Rank:

Features of a Culinary Medicine Course for Undergraduate UK Medical Students

13) In which year(s) of the MBBS course do you think the Culinary Medicine course should be delivered? *Please tick as many boxes as appropriate*.

Year 1	Year 2	Year 4	Year 5	Final Year

14) Do you think Culinary Medicine should be taught as a horizontal (systems-based) module or vertical module? *Please tick one box*.

Horizontal	Vertical

For peer review only - http://bmjopen.bmj.com/site/about/guidelines.xhtml

15) What knowledge and skills do you think you should gain from an undergraduate Culinary Medicine course?

....

....

....

....

16) Please list 3 topics that you think should be taught on the Culinary Medicine course.

....

....

....

If you have any additional comments, please write them below:

....

....

....

....

....

....

....

....

....

Thank you for taking the time to complete this questionnaire.

For peer review only - http://bmjopen.bmj.com/site/about/guidelines.xhtml

Appendix 5 tParticipant Recruitment Flyer and Email to Society

Dear [society name],

My name is Jessica Xie, a fourth-year medical student. I am writing to you to ask if you would please assist me in a research project.

UCL Medical School is currently developing a new Culinary Medicine course for its undergraduate medical students. With the help of staff at the UCL Primary Care and Population Health department, I have designed an online questionnaire for UCL medical students with the aim of gaining of an understanding of the preferences and priorities of students towards nutrition education. The results of this study will be used to aid the design and development of a Culinary Medicine course. The ultimate aim of this study is to improve the training of future UCL medical students.

I would be grateful if you would please distribute the following link to your society members and/or include the link on your website/ on your social media platforms/ in your e-newsletters/ in the next edition of the RUMS Review:

[weblink to questionnaire]

Please note that student participation in this study is entirely voluntary, is outlined in the link above and only students of UCL Medical School in the academic year 2018-19 are eligible to take part. The questionnaires are completed anonymously.

If you have any questions, please do not hesitate to contact me.

Thank you in advance for your help.

Kind regards,

Jessica Xie

MBBS Year 4

UCL Medical School

For peer review only

UCL

Interested in Culinary Medicine? Want to Contribute to Undergraduate Medical Education?

If you are a UCL Medical student, tell us what you think about learning Culinary Medicine!

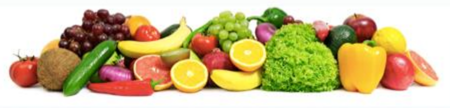

UCL Medical School is developing a **Culinary Medicine course** for its undergraduate medical students.

We need your help to make this course as **interesting**, **engaging** and **informative** as possible for future students!

Please take our **15-minute survey**, which will ask you about your **understanding** and **experiences** of learning Culinary Medicine and for your opinion on the **ideal features** of a Culinary Medicine course.

The National Institute for Health and Care Excellence (NICE) envisages that, in less than two years from now, **improving patients' diets** could become the **first-line intervention** to tackle conditions such as **heart disease** and **cancer**[1].

This survey is to be completed by students of UCL Medical School during the academic year 2018-19 only.

Survey responses are anonymous and participants are not asked to share any personal information that they can be identified from.

For more information about this study, please email Jessica Xie at jessica.xie.15@ucl.ac.uk.

Complete the survey: [link]

[1] Womersley, K. and Ripullone, K.. Medical schools should be prioritising nutrition and lifestyle education. The British Medical Journal (BMJ) Opinion. Weblog. [Online]. Available from: https://blogs.bmj.com/bmj/2017/09/07/medical-schools-should-be-prioritising-nutrition-and-lifestyle-education/ [Cited 30th July 2018].

For peer review only - http://bmjopen.bmj.com/site/about/guidelines.xhtml

Appendix 6 tParticipant Information Sheet (PIS)

Study Title

A survey of the preferences and expectations of UK undergraduate medical students from a Culinary Medicine course.

Invitation to Take Part in A Research Project

You are being invited to take part in a research project. Before you decided it is important for you to understand why the research us being done and what participation will involve. Please take time to read the following information carefully and discuss it with others if you wish. Ask us if there is anything that is not clear or if you would like more information. Take time to decide whether or not you wish to take part. Thank you for reading this.

What is the project's purpose?

Culinary Medicine is a new evidence-based field in Medicine that combines nutrition and culinary knowledge to improve the health and wellbeing of patients.

University College London (UCL) Medical School is in currently developing a Culinary Medicine course for its undergraduate students.

The aim of this project is to determine UCL medical students' learning needs and preferences in the area of Culinary Medicine with a view to informing development of a Culinary Medicine course at UCL.

The objectives of this project are:

- To determine undergraduate UCL medical students' understanding of Culinary Medicine and their preferences and expectations of a Culinary Medicine course in their medical programme.
- To determine the level of experiences and/or training in Culinary Medicine.
- To determine undergraduate medical students' perception of their current abilities to facilitate discussion about diet and provide dietary advice for patients.
- To evaluate the level of satisfaction of undergraduate medical students with the current quality and quantity of Culinary Medicine training delivered by UCL Medical School.

This project will run from November 2018 – February 2019.

Why have I been chosen?

You have been chosen to take part in this study because you are currently a medical student at UCL. We have asked various UCL and RUMS student societies and organisations to distribute the weblink to this questionnaire to UCL medical students. This questionnaire should not be completed by anyone who is not currently a UCL medical student.

Do I have to take part?

It is up to you to decide whether or not to take part. If you do decide to take part, it is important that you read and fully understand this participant information sheet and read, fully understand and complete the consent form (next page).

For peer review only - http://bmjopen.bmj.com/site/about/guidelines.xhtml

You may withdraw from the study at any point in time, without having to provide a reason for your withdrawal.

What will happen to me if I take part?

The study involves you completing an online questionnaire that should take no longer than 15 minutes to complete.

The questionnaires are completed anonymously. Your personal details (name, age and demographic data) will be not be recorded. We will ask for your year group of study. Therefore, it will not be possible for researchers to contact you

What are the possible disadvantages or risks of taking part?

There is a very low risk of breach of confidentiality.

All the information that we collect from your questionnaire response will be kept strictly confidential, as no participant-identifying data will be collected.

Only the research and analysis team will have access to the data.

Your questionnaire responses will be used for research purposes only.

What are the possible benefits of taking part?

The importance of nutrition and diet in undergraduate medical education teaching across all disciplines has long been under-recognized. It is reported that the majority doctors in the UK feel incompetent to provide patients with lifestyle advice.

By taking part in this project, you may be contributing to the training of future UCL medical students, and therefore future doctors.

What if something goes wrong?

If you would like to raise any concerns or complaints, please contact the chief investigator, Dr Sophie Park, at sophie.park@ucl.ac.uk.

What will happen to the results of this research project?

Your questionnaire responses may be used to develop the Culinary Medicine course in the MBBS undergraduate programme that is due to be introduced into the curriculum in 2019.

We aim to finish collecting and analysing the data around February 2019. If you would like to obtain a copy of the results of this research project please contact the principal researcher, Jessica Xie at: jessica.xie.15@ucl.ac.uk.

Data Protection Privacy Notice

Notice: The data controller for this project will be University College London (UCL). The UCL Data Protection Office provides oversight of UCL activities involving the processing of personal data, and can be contacted at data-protection@ucl.ac.uk. UCL's Data Protection Officer can also be contacted at data-protection@ucl.ac.uk.

Further information on how UCL uses participant information can be found here:

www.ucl.ac.uk/legal-services/privacy/participants-health-and-care-research-privacy-notice

For peer review only - http://bmjopen.bmj.com/site/about/guidelines.xhtml

The only data that will be collected are the anonymised questionnaire responses and the year group of study participants.

In keeping with UCL Data Protection regulations, year group is not considered personal data as data subjects cannot be identified either directly or indirectly by reference to their year group.

Provided that your responses do not contain any data that may suggest that you completed the questionnaire, we will not collect any identifying data or pseudonymized data from you. Each response will be given an identification number but there will be no person-identifiable data. Therefore, it will not be possible to trace the identification number and the response back to you.

You have certain rights under data protection legislation in relation to the personal information that is held about you. These rights apply only in particular circumstances and are subject to certain exemptions such as public interest (for example the prevention of crime). They include:

- The right to access your personal information;
- The right to rectification of your personal information;
- The right to erasure of your personal data;
- The right to restrict or object to the processing of your personal data;
- The right to object to the use of your data for direct marketing purposes;
- The right to data portability;
- Where the justification for processing is based on your consent, the right to withdraw such consent at any time; and
- The right to complain to the Information Commissioner's Office (ICO) about the use of your personal data.

If you are concerned about how your personal data is being processed, or if you would like to contact us about your rights, please contact UCL in the first instance at data-protection@ucl.ac.uk.

If you remain unsatisfied, you may wish to contact the ICO. Contact details, and further details of data subject rights, are available on the ICO website at: https://ico.org.uk/for-organisations/data-protection-reform/overview-of-the-gdpr/individuals-rights/

Who is organising and funding the research?

This project is organised by the UCL department of Primary Care and Population Health and is funded by National Institute for Health Research (NIHR) School for Primary Care Research (SPCR).

Contact for further information

If you have any questions or comments, or would like to obtain a copy of the results please contact the principal researcher, Jessica Xie at: jessica.xie.15@ucl.ac.uk or the chief investigator, Dr Sophie Park, at sophie.park@ucl.ac.uk.

Thank you for reading this information sheet and for considering to take part in this research study.

For peer review only - http://bmjopen.bmj.com/site/about/guidelines.xhtml

For peer review only

Appendix 7 tConsent Form

CONSENT FORM

Please tick boxes

Title of Project: A survey of the preferences and expectations of UK undergraduate medical students from a Culinary Medicine course.

Name of Researcher: Jessica Xie

1. I confirm that I have read the participant information (above) for this study. I have had the opportunity to consider the information, ask questions and have had these answered satisfactorily. ☐

2. I understand that my participation is voluntary and that I am free to withdraw at any time without giving any reason, without my medical care or legal rights being affected. ☐

3. I understand that the information collected about me will be used to support other research in the future, and may be shared anonymously with other researchers. ☐

4. I understand that the information collected about me will be used to support other research in the future, and may be shared anonymously with other researchers. ☐

5. I agree to take part in the above study. ☐

For peer review only

For peer review only - http://bmjopen.bmj.com/site/about/guidelines.xhtml

Culinary Medicine Course for UK Undergraduate Medical Students

SUMMARY

• "UK doctors must be competent in recognising ill health as a result of poor nutrition, and applying the principles, methods and dietary knowledge to medical practice and integrating these into patient care" - GMC (2018).
• In September 2019, UCL Medical School will introduce “Primary Care Nutrition”, a Culinary Medicine course, into the MBBSY5 curriculum.
• Help shape the MBBS course prior to its launch to maximise your learning potential and improve the training of future UCL medical students!
• Study aim: to determine UCL medical students’ learning needs and preferences in the area of Culinary Medicine with a view to informing development of a Culinary Medicine course at UCL.
• This questionnaire will take no longer than 5 minutes to complete.

// Read the full Participant Information Sheet (directly below) or click NEXT (bottom of page) \\

****

A survey of the preferences and expectations of UK undergraduate medical students from a Culinary Medicine course.

1) Invitation to Take Part in A Research Project

You are being invited to take part in a research project which will involve you completing a questionnaire consisting of 15 questions. Before you decided it is important for you to understand why the research us being done and what participation will involve. Please take time to read the following information carefully and discuss it with others if you wish. Ask us if there is anything that is not clear or if you would like more information. Take time to decide whether or not you wish to take part. Thank you for reading this.

2) What is the project’s purpose?

Culinary Medicine is a new evidence-based field in Medicine that combines nutrition and culinary knowledge to improve the health and wellbeing of patients.

The aim of this project is to determine UCL medical students’ learning needs and preferences in the area of Culinary Medicine with a view to informing development of a Culinary Medicine course at UCL.

The objectives of this study are:
> To determine undergraduate UCL medical students’ understanding of Culinary Medicine and their preferences and expectations of a Culinary Medicine course in their medical programme.
> To determine the level of experiences and/or training in Culinary Medicine.
> To determine undergraduate medical students’ perception of their current abilities to facilitate discussion about diet and provide dietary advice for patients.
> To evaluate the level of satisfaction of undergraduate medical students with the current quality and quantity of Culinary Medicine training delivered by UCL Medical School. This project will run from December 2018 – February 2019.

3) Why have I been asked to take part?

You have been asked to take part in this study because you are currently a medical student at UCL. We have asked various UCL and RUMS student societies and organisations to distribute the weblink to this questionnaire to UCL medical students. This questionnaire should not be completed by anyone who is not currently a UCL medical student.

4) Do I have to take part?

It is up to you to decide whether or not to take part. If you do decide to take part, it is important that you read and fully understand this participant information sheet and read, fully understand

and complete the consent form (next page).

You may withdraw from the study at any point in time, without having to provide a reason for your withdrawal.

5) What will happen to me if I take part?

The study involves you completing an online questionnaire that should take no longer than 10 minutes to complete.

The questionnaires are completed anonymously. Your personal details (name, age and demographic data) will be not be recorded. We will ask for your year group of study. Therefore, it will not be possible for the researchers and data analysts to contact you.

6) What are the possible disadvantages or risks of taking part?

There is a very low risk of breach of confidentiality.

No participant-identifying data will be collected. All the information that we collect from your questionnaire response will be kept strictly confidential.

Only the research and analysis team will have access to the data. Data will not be passed onto third parties.

Your questionnaire responses will be used for research purposes only.

7) What are the possible benefits of taking part?

The importance of nutrition and diet in undergraduate medical education teaching across all disciplines has long been under-recognised. It is reported that the majority of doctors in the UK feel incompetent to provide patients with lifestyle advice.

By taking part in this project, you may be contributing to the training of current and future UCL medical students, and therefore future doctors.

8) What if something goes wrong?

If you would like to raise any concerns or complaints, please contact the chief investigator, Dr Sophie
Park, at sophie.park@ucl.ac.uk.

9) What will happen to the results of this research project?

Your questionnaire responses may be used to develop the Culinary Medicine course in the MBBS undergraduate programme that is due to be introduced into the curriculum in 2019.

We aim to finish collecting and analysing the data around February 2019. If you would like to obtain a copy of the results of this research project please contact the principal researcher, Jessica Xie, at: jessica.xie.15@ucl.ac.uk.

10) Data Protection Privacy Notice

Notice: The data controller for this project will be University College London (UCL). The UCL Data Protection Office provides oversight of UCL activities involving the processing of personal data, and can be contacted at data-protection@ucl.ac.uk. UCL's Data Protection Officer can also be contacted at data-protection@ucl.ac.uk.

Further information on how UCL uses participant information can be found here:
www.ucl.ac.uk/legal-services/privacy/participants-health-and-care-research-privacy-notice
53.

The only data that will be collected are the anonymised questionnaire responses and the year group of study participants.
In keeping with UCL Data Protection regulations, year group is not considered personal data as data subjects cannot be identified either directly or indirectly by reference to their year group.

Provided that your responses do not contain any data that may suggest that you completed the

questionnaire, we will not collect any identifying data or pseudonymized data from you. Each response will be given an identification number but there will be no person-identifiable data. Therefore, it will not be possible to trace the identification number and the response back to you.

You have certain rights under data protection legislation in relation to the personal information that is held about you. These rights apply only in particular circumstances and are subject to certain exemptions such as public interest (for example the prevention of crime). They include:
• The right to access your personal information;
• The right to rectification of your personal information;
• The right to erasure of your personal data;
• The right to restrict or object to the processing of your personal data;
• The right to object to the use of your data for direct marketing purposes;
• The right to data portability;
• Where the justification for processing is based on your consent, the right to withdraw such consent at any time; and
• The right to complain to the Information Commissioner's Office (ICO) about the use of your personal data.

If you are concerned about how your personal data is being processed, or if you would like to contact us about your rights, please contact UCL in the first instance at data-protection@ucl.ac.uk.

If you remain unsatisfied, you may wish to contact the ICO. Contact details, and further details of data subject rights, are available on the ICO website at: https://ico.org.uk/for-organisations/data-protection-reform/overview-of-the-gdpr/individuals-rights/.

10) Who is organising and funding the research?

This project is being developed with the help of Dr Rupy Aujla by the The UCL Primary Care Medical Education team in the UCL department of Primary Care and Population Health and is funded by National Institute for Health Research (NIHR) School for Primary Care Research (SPCR).

11) If you have any questions or comments, or would like to obtain a copy of the results please contact the principal researcher, Jessica Xie at: jessica.xie.15@ucl.ac.uk or the chief investigator, Dr Sophie Park, at sophie.park@ucl.ac.uk.

Thank you for reading this information sheet and for considering to take part in this research study.

* Required

Consent Form

1. **Please tick each box to consent to taking part in this study. ***
   Check all that apply.

   - [ ] I confirm that I have read the participant information (above) for this study. I have had the opportunity to consider the information, ask questions and have had these answered satisfactorily.
   - [ ] I understand that my participation is voluntary and that I am free to withdraw at any time without giving any reason, without my medical education or legal rights being affected.
   - [ ] I understand that the information collected about me will be used to support other research in the future, and may be shared anonymously with other researchers.
   - [ ] I agree to take part in this study.

About You

2. **Which year of the medical (MBBS) programme are you currently in? ***

Mark only one oval.

- Year 1
- Year 2
- Year 3
- Year 4
- Year 5
- Year 6 (Final Year)

3. **What is your career intention? Select 1 option that is most accurate at present.** *

Mark only one oval.

- Academic Medicine
- Acute Medicine
- Anaesthesia
- Cardiology
- Dermatology
- Emergency Medicine
- Ears, Nose and Throat
- Endocrinology
- Gastroenterology
- General Medicine
- General Practice
- Geriatric Medicine
- Haematology
- Histopathology
- Infectious Diseases
- Nephrology
- Neurology
- Obstetrics and Gynaecology
- Ophthalmology
- Orthopaedics
- Paediatrics and Child Health
- Palliative Medicine
- Pathology
- Psychiatry
- Public Health Medicine
- Radiology
- Respiratory Medicine
- Rheumatology
- Sport and Exercise Medicine
- Surgery
- Tropical Medicine
- Urology
- A career not in Medicine
- Undecided
- Other: ____________________

Education

4. **What do you understand by the term 'Culinary Medicine'? ***

5. **Which of the following, if any, Culinary Medicine teaching have you received so far in the MBBS programme? Choose as many statements as relevant. ***
Check all that apply.

- [ ] Problem-based learning from patient cases
- [ ] Practical kitchen session
- [ ] Evidence evaluation
- [ ] Self-directed learning
- [ ] Small-group seminars/ tutorials
- [ ] Lectures
- [ ] Webinar (internet) version of the lecture or seminar
- [ ] None
- [ ] Other: ______________________

6. **Have you received any extracurricular Culinary Medicine teaching? If so, please describe below, including which organisation(s) ran the session, the topic(s) that were covered, the style of teaching and your opinion of the learning experience**
Mark only one oval.

- ( ) No
- ( ) Other: ______________________

Communication and Understanding

To what extent you agree with the following statements:

7. **I am able to facilitate a discussion with patients about diet and/or dietary change. ***
Mark only one oval.

	1	2	3	4	5	
Strongly disagree	( )	( )	( )	( )	( )	Strongly agree

8. **I am able to take dietary history from a patient. ***
Mark only one oval.

	1	2	3	4	5	
Strongly disagree	( )	( )	( )	( )	( )	Strongly agree

9. **I am able to apply principles, methods and knowledge relating to nutrition to medical practice and integrate these into patient care.** *

Mark only one oval.

	1	2	3	4	5	
Strongly disagree	◯	◯	◯	◯	◯	Strongly agree

10. **I am able to provide dietary advice for patients with varying cultural, social and economic needs.** *

Mark only one oval.

	1	2	3	4	5	
Strongly disagree	◯	◯	◯	◯	◯	Strongly agree

Delivery of an Culinary Medicine Course for UK Undergraduate Medical Students

To what extent you agree with the following statements:

11. **I believe that learning Culinary Medicine is important for my future clinical practice.** *

Mark only one oval.

	1	2	3	4	5	
Strongly disagree	◯	◯	◯	◯	◯	Strongly agree

12. **I am satisfied with the QUALITY of Culinary Medicine training that my medical school provides** *

Mark only one oval.

	1	2	3	4	5	
Strongly disagree	◯	◯	◯	◯	◯	Strongly agree

13. **I am satisfied with the QUANTITY of Culinary Medicine training that my medical school provides** *

Mark only one oval.

	1	2	3	4	5	
Strongly disagree	◯	◯	◯	◯	◯	Strongly agree

14. **Select the 3 most appropriate teaching methods for a Culinary Medicine course (N.B 1 or more of the below may be used in combination in teaching).** *

Check all that apply.

- [ ] Problem-based learning from patient cases
- [ ] Practical kitchen session
- [ ] Evidence evaluation
- [ ] Self-directed learning
- [ ] Small-group seminars/ tutorials
- [ ] Lectures
- [ ] Webinar (internet) version of the lecture or seminar
- [ ] Other: ____________________

Features of a Culinary Medicine Course for Undergraduate UK Medical Students

15. **In which year(s) of the MBBS course do you think the Culinary Medicine course should be delivered? Select as many options as appropriate.** *

Check all that apply.

- [ ] Year 1
- [ ] Year 2
- [ ] Year 3
- [ ] Year 4
- [ ] Year 5
- [ ] Year 6 (Final Year)

16. **Do you think Culinary Medicine should be taught as/within a horizontal (i.e systems-based (e.g. Circulation and Breathing) module or vertical module (e.g. Pharmacology)?** *

Mark only one oval.

- ( ) Horizontal
- ( ) Vertical

17. **Which topics and/or skills do you think should be taught on the Culinary Medicine course? Select as many options as appropriate or offer a suggestion.** *

Check all that apply.

- [ ] Weight management and portion control
- [ ] Types of diet (e.g. "Mediterranean" and Dietary Approaches to Stop Hypertension (DASH)) and their evidence-base
- [ ] Paediatric and geriatric diets
- [ ] Nutrition psychology
- [ ] Dietary needs of patients with varying cultural, social and economic backgrounds
- [ ] Taking a dietary history
- [ ] Applying principles, methods and knowledge relating to nutrition to medical practice and integrating these into patient care
- [ ] Practical culinary skills
- [ ] Other: ______________________

18. **If you have any additional comments, please write them below:**

____
____
____
____
____

Thank you for completing this questionnaire!

Questions should be directed to Jessica Xie (jessica.xie.15@ucl.ac.uk) or Dr Sophie Park (sophie.park@ucl.ac.uk).

Here are some links that can provide you with more information about Culinary Medicine:
> http://www.culinarymedicineuk.org
> https://www.ncbi.nlm.nih.gov/pmc/articles/PMC4739343/
> https://www.telegraph.co.uk/health-fitness/nutrition/diet/culinary-medicine-need-unlock-secrets-medical-kitchen-cabinet/
> https://www.telegraph.co.uk/health-fitness/nutrition/food-medicine-arent-doctors-trained-science-nutrition/

Powered by
Google Forms

Standards for Reporting Qualitative Research (SRQR)*

http://www.equator-network.org/reporting-guidelines/srqr/

Page/line no(s).

Title and abstract

**Title** - Concise description of the nature and topic of the study Identifying the study as qualitative or indicating the approach (e.g., ethnography, grounded theory) or data collection methods (e.g., interview, focus group) is recommended	Page 1, lines 1 - 5
**Abstract** - Summary of key elements of the study using the abstract format of the intended publication; typically includes background, purpose, methods, results, and conclusions	Page 2, lines 1 - 30

Introduction

**Problem formulation** - Description and significance of the problem/phenomenon studied; review of relevant theory and empirical work; problem statement	Page 3, lines 15 – 26, 37 – 41
**Purpose or research question** - Purpose of the study and specific objectives or questions	Page 3, line 41<br>Page 4, line 1 - 2

Methods

**Qualitative approach and research paradigm** - Qualitative approach (e.g., ethnography, grounded theory, case study, phenomenology, narrative research) and guiding theory if appropriate; identifying the research paradigm (e.g., postpositivist, constructivist/ interpretivist) is also recommended; rationale**	Page 4, lines 4 – 8, 20 - 23
**Researcher characteristics and reflexivity** - Researchers' characteristics that may influence the research, including personal attributes, qualifications/experience, relationship with participants, assumptions, and/or presuppositions; potential or actual interaction between researchers' characteristics and the research questions, approach, methods, results, and/or transferability	Page 4, lines 5 – 8<br><br>Page 5, lines 14 – 17
**Context** - Setting/site and salient contextual factors; rationale**	Page 4, lines 9 - 10
**Sampling strategy** - How and why research participants, documents, or events were selected; criteria for deciding when no further sampling was necessary (e.g., sampling saturation); rationale**	Page 4, lines 11 – 14
**Ethical issues pertaining to human subjects** - Documentation of approval by an appropriate ethics review board and participant consent, or explanation for lack thereof; other confidentiality and data security issues	Page 4, lines 15 – 21<br>Page 5, lines 11 - 12
**Data collection methods** - Types of data collected; details of data collection procedures including (as appropriate) start and stop dates of data collection and analysis, iterative process, triangulation of sources/methods, and modification of procedures in response to evolving study findings; rationale**	Page 4, lines 4 – 5, lines 22 – 25<br><br>Page 5, lines 5 - 9

For peer review only

For peer review only - http://bmjopen.bmj.com/site/about/guidelines.xhtml

**Data collection instruments and technologies** - Description of instruments (e.g., interview guides, questionnaires) and devices (e.g., audio recorders) used for data collection; if/how the instrument(s) changed over the course of the study	Page 4, lines 4 – 5
**Units of study** - Number and relevant characteristics of participants, documents, or events included in the study; level of participation (could be reported in results)	Page 4, lines 9 – 12, 20 – 21<br><br>Page 6, lines 12 - 14
**Data processing** - Methods for processing data prior to and during analysis, including transcription, data entry, data management and security, verification of data integrity, data coding, and anonymization/de-identification of excerpts	Page 5, lines 15 - 23<br>Page 6, lines 1 - 2
**Data analysis** - Process by which inferences, themes, etc., were identified and developed, including the researchers involved in data analysis; usually references a specific paradigm or approach; rationale**	Page 5, lines 15 - 23<br>Page 6, lines 1 - 2
**Techniques to enhance trustworthiness** - Techniques to enhance trustworthiness and credibility of data analysis (e.g., member checking, audit trail, triangulation); rationale**	Page 5, lines 16 - 18<br>Page 12, lines 21 - 23

Results/findings

**Synthesis and interpretation** - Main findings (e.g., interpretations, inferences, and themes); might include development of a theory or model, or integration with prior research or theory	Page 6, line 15 - page 10, line 11
**Links to empirical data** - Evidence (e.g., quotes, field notes, text excerpts, photographs) to substantiate analytic findings	Page 7, line 1 – page 10, line 11

Discussion

**Integration with prior work, implications, transferability, and contribution(s) to the field -** Short summary of main findings; explanation of how findings and conclusions connect to, support, elaborate on, or challenge conclusions of earlier scholarship; discussion of scope of application/generalizability; identification of unique contribution(s) to scholarship in a discipline or field	Page 11, lines 15– 40<br><br>Page 12 lines 1 – 15, 37 – 39<br>Page 13, lines 1 - 39
**Limitations** - Trustworthiness and limitations of findings	Page 12, lines 24 - 39

Other

For peer review only

For peer review only - http://bmjopen.bmj.com/site/about/guidelines.xhtml

**Conflicts of interest** - Potential sources of influence or perceived influence on study conduct and conclusions; how these were managed	Page 14, line 34
**Funding** - Sources of funding and other support; role of funders in data collection, interpretation, and reporting	Page 14, lines 30 - 32

*The authors created the SRQR by searching the literature to identify guidelines, reporting standards, and critical appraisal criteria for qualitative research; reviewing the reference lists of retrieved sources; and contacting experts to gain feedback. The SRQR aims to improve the transparency of all aspects of qualitative research by providing clear standards for reporting qualitative research.

**The rationale should briefly discuss the justification for choosing that theory, approach, method, or technique rather than other options available, the assumptions and limitations implicit in those choices, and how those choices influence study conclusions and transferability. As appropriate, the rationale for several items might be discussed together.

For peer review only

Reference:

O'Brien BC, Harris IB, Beckman TJ, Reed DA, Cook DA. **Standards for reporting qualitative research: a synthesis of recommendations.** *Academic Medicine*, Vol. 89, No. 9 / Sept 2014 DOI: 10.1097/ACM.0000000000000388

BMJ Open

A Cross-sectional Questionnaire Study to Gather the Teaching Preferences and Expectations of UK Undergraduate Medical Students For Culinary Medicine Learning

Journal:	*BMJ Open*
Manuscript ID	bmjopen-2019-036410.R2
Article Type:	Original research
Date Submitted by the Author:	03-Aug-2020
Complete List of Authors:	Xie, Jessica; University College London Research Department of Primary Care and Population Health,<br>Poduval, Shoba; University College London, Research Department of Primary Care & Population Health<br>Vickerstaff, Victoria; University College London, Division of Psychiatry; University College London, University College London Research Department of Primary Care and Population Health<br>Park, Sophie; University College London Research Department of Primary Care and Population Health
**Primary Subject Heading**:	Medical education and training
Secondary Subject Heading:	Nutrition and metabolism, General practice / Family practice, Medical education and training
Keywords:	MEDICAL EDUCATION & TRAINING, NUTRITION & DIETETICS, EDUCATION & TRAINING (see Medical Education & Training), PRIMARY CARE

SCHOLARONE™
Manuscripts

I, the Submitting Author has the right to grant and does grant on behalf of all authors of the Work (as defined in the below author licence), an exclusive licence and/or a non-exclusive licence for contributions from authors who are: i) UK Crown employees; ii) where BMJ has agreed a CC-BY licence shall apply, and/or iii) in accordance with the terms applicable for US Federal Government officers or employees acting as part of their official duties; on a worldwide, perpetual, irrevocable, royalty-free basis to BMJ Publishing Group Ltd ("BMJ") its licensees and where the relevant Journal is co-owned by BMJ to the co-owners of the Journal, to publish the Work in this journal and any other BMJ products and to exploit all rights, as set out in our licence.

The Submitting Author accepts and understands that any supply made under these terms is made by BMJ to the Submitting Author unless you are acting as an employee on behalf of your employer or a postgraduate student of an affiliated institution which is paying any applicable article publishing charge ("APC") for Open Access articles. Where the Submitting Author wishes to make the Work available on an Open Access basis (and intends to pay the relevant APC), the terms of reuse of such Open Access shall be governed by a Creative Commons licence – details of these licences and which Creative Commons licence will apply to this Work are set out in our licence referred to above.

Other than as permitted in any relevant BMJ Author's Self Archiving Policies, I confirm this Work has not been accepted for publication elsewhere, is not being considered for publication elsewhere and does not duplicate material already published. I confirm all authors consent to publication of this Work and authorise the granting of this licence.

For peer review only

A Cross-sectional Questionnaire Study to Gather the Teaching Preferences and Expectations of UK Undergraduate Medical Students For Culinary Medicine Learning

For peer review only

Author 1: Miss Jessica Ying-Yi Xie

Research Department of Primary Care and Population Health, Upper Third Floor, UCL Medical School, Rowland Hill Street, London, NW3 2PF, UK

jx9988_@hotmail.co.uk

Author 2: Dr Shoba Poduval

Research Department of Primary Care and Population Health, Upper Third Floor, UCL Medical School, Rowland Hill Street, London, NW3 2PF, UK

s.poduval@ucl.ac.uk

+44 (0)20 8016 8357

Author 3: Dr Victoria Vickerstaff

Division of Psychiatry, UCL, 6th Floor, Wing B, Maple House, 149 Tottenham Court Road London, W1T 7NF

Research Department of Primary Care and Population Health, Upper Third Floor, UCL Medical School, Rowland Hill Street, London, NW3 2PF, UK
v.vickerstaff@ucl.ac.uk

+44 (0)20 7679 9721

Author 4: Dr Sophie Park (Corresponding Author)

Research Department of Primary Care and Population Health, Upper Third Floor, UCL Medical School, Rowland Hill Street, London, NW3 2PF, UK

sophie.park@ucl.ac.uk

+44 (0)20 8016 8276

Total word count: 3893

Keywords: undergraduate medical education, quantitative research, questionnaire, culinary medicine, general practice, primary care

3 tables, 4 figures

Abstract

Aim: To determine undergraduate medical students' teaching preferences and expectations for Culinary Medicine (CM) learning with a view to informing development of a CM course at a UK medical school.

Setting: A single, urban UK medical school.

Participants: 180 undergraduate medical students.

Study Design: A cross-sectional questionnaire study collecting quantitative and qualitative (free-text) data.

Methods and Outcome Measures: An online questionnaire consisting of 16 questions of various styles (Likert-type, multiple choice and free-text). Quantitative analysis of multiple choice and Likert-type scale questions was conducted. Qualitative thematic analysis was used to analyse the free-text responses and identify themes.

Results: Three core themes related to students' understanding of CM were identified: (1) "CM Learning": students' perceived relevance of CM knowledge, perceived relevance of CM to healthcare, and their expectations for teaching; (2) "The Relationship between Food and Health": links between diet, social factors and health; and (3) "Evidence-based Medicine": students' perceptions about scientific principles underlying CM. Quantitative analysis revealed that, although 83% of students felt that learning CM is important for their future clinical practice, 56% felt unable to take a dietary history. 73% of students were dissatisfied with the quality, and 78% were dissatisfied with the quantity, of existing medical school teaching understood to be relevant to CM. Topics that students would like to be taught on a CM course included weight management and portion control. Students felt that problem-based-styled learning would be the most appropriate method for delivering CM teaching.

Conclusions: This study revealed that medical students felt their dietary counselling skills could be improved with further clinically-relevant teaching in the undergraduate medical curriculum. Students' preferences for CM learning have been taken into consideration in the development of a CM course for fifth-year undergraduate students at a UK medical school, which is delivered during their General Practice placements.

For peer review only

Article Summary

Strengths and Limitations of This Study

- The questionnaire was piloted to confirm readability and ensure that questions addressed the study aim.
- At interim analysis of the first 148 questionnaire responses, no new codes were generated during qualitative thematic analysis of free-text responses, signifying that theoretical data saturation had been reached.
- The study was conducted at one medical school only, limiting the transferability of the study findings to other medical schools.
- The response rate (11%) was low which limits the generalisability of the results to the student population.

Introduction

Culinary Medicine (CM) may be defined as "an evidence-based field in Medicine that blends the art of food and cooking with the science of Medicine".[(1)] A CM course can offer online learning, didactic teaching and practical hands-on culinary skills in a kitchen setting. The aim is to give students the knowledge and skills to help patients make informed decisions about accessing and eating healthy meals. The General Medical Council (GMC) expects UK medical school graduates to be competent in recognising ill health as a result of poor nutrition, and to be able to apply dietary knowledge to clinical practice.[(2)] However, it has been reported that medical students and doctors worldwide feel under-equipped to provide dietary advice for patients.[(3–6)] Studies have found that medical students who learn CM are likely to develop positive attitude towards, and better knowledge of, diet and nutrition; adopt better health habits themselves; and become more competent and confident in dietary counselling.[(7–10)]

The setting for this study was a single, urban UK medical school, with a five-year undergraduate medical (MBBS) curriculum plus an Integrated Bachelor of Science (iBSc) in the third year. The first three years are the pre-clinical part of the MBBS curriculum. In their first year, students receive lectures on nutritional science, such as the digestion of macronutrients and gastrointestinal physiology, and public health nutrition.

The Primary Care Medical Education Team at this UK medical school collaborated with a team of doctors, dietitians and chefs from Culinary Medicine UK[(11)] to create a CM course that was introduced into the Primary Care module of the fifth year of the MBBS curriculum in September 2019. Teaching consisted of online learning and one day of face-to-face teaching in a teaching kitchen. The course teaches students culinary skills, evidence-based medicine related to dietary counselling and motivational interviewing skills. Believed to be the first medical school in Europe to provide mandatory CM teaching, the aims of the course are to improve students' confidence in raising the subject of healthy eating and in giving simple dietary advice based on evidence-based recommendations; and to increase students' awareness of potential barriers to healthy eating (for example, cultural norms and low household income). This study was conducted prior to the introduction of the CM course to

For peer review only

inform its development by determining students' teaching preferences and expectations for CM learning.

Methods

This was a cross-sectional questionnaire study, collecting quantitative and qualitative data using an online (Google Forms) questionnaire. JX (a fourth-year medical student and National Institute for Health Research School for Primary Care Research (NIHR SPCR) intern in the University College London (UCL) Research Department of Primary Care and Population Health), led the study design, data collection and analysis.

The study was conducted at a single, urban UK medical school from December 2018 to April 2019.

The study population was 1669 undergraduate medical students enrolled in the academic year 2018/19 (data supplied by UCL Student Data Analyst).

The Questionnaire

There was a total of 16 questions of various styles (Likert-type, multiple choice and free-text) across four main topics (see Table 1). The first topic included establishing students' understanding of the term 'CM'. A free-text response was requested to explore the differences in students' understanding and interpretation of the concept.

Table 1: *The topic, description and style of question(s) included in the questionnaire.*

Topic	Information to Gather	Question type
(1) Education	Students' understanding of the term 'CM'<br>Previous experience(s) of teaching understood to be relevant to CM	Free-text
(2) Integrating CM knowledge into clinical practice	Students' self-perceived abilities to facilitate discussions about diet and provide dietary advice for patients	Likert-type
(3) Teaching CM	Students' satisfaction with existing medical school teaching understood to be relevant to CM	Multiple choice and Likert-type
(4) Features of a CM course	Students' expectations for a CM course for undergraduate medical students, including methods of delivering teaching and teaching topics	Multiple choice

For peer review only

At the end of the questionnaire, a free-text box was provided for additional general comments, as well as weblinks for students who wished to learn more about CM.

Patient and Public Involvement

Prior to data collection, two medical students (iBSc third-year and MBBS fifth-year) were invited to pilot the questionnaire and comment on the readability. These students suggested re-wording of a few questions to improve clarity. The questionnaire was updated accordingly. Students were not invited to contribute to the study design, analysis or writing of this manuscript.

Data Collection

The questionnaire was advertised on posters and via social media and student societies' e-newsletters.

Ethical Approval

Ethical approval to carry out this study was received from UCL Research Ethics Committee (ref.12471/002).

Study participants were asked provide informed consent by reading the participant information sheet (PIS) on the first page of the questionnaire and confirming that they fully understood their role in the study and how the data collected would be used. Once consent had been gained, participants were able to proceed to the questionnaire on the next page.

Questionnaires were completed anonymously. No participant-identifiable data was collected. The only personal data collected was year of study, but participants could not be identified from the information that they provided.

Analysis

Qualitative

NVivo 12[12] was used to store free-text data in order to conduct a qualitative thematic analysis and identify core themes.[13] JX and ShP independently coded all free-text responses before sharing their interpretations with the qualitative results analysis team (JX, ShP and SP) to discuss any areas of dissonance or contrasting interpretation. Codes were derived deductively from the questionnaire (for example, 'Understanding of CM'), and inductively (for example, 'Social factors contributing to diet') upon data analysis.[14] Careful consideration was given upon coding: new codes were compared against preliminary codes and refined, and the data was re-coded using the updated set of codes. This improved the quality of analysis.[15] Similar codes were categorized under a sub-theme or core theme. No new codes emerged after coding responses from the first 148 students in an interim analysis, suggesting that theoretical data saturation had been reached.

Quantitative

SPSS Statistics 25[16] was used to conduct the quantitative analysis. The quantitative results analysis team (JX and VV) carried out descriptive statistics to describe students' year of study. Responses to Likert-type and multiple-choice questions were presented using proportions and frequencies, and responses from pre-clinical (years one to three) and clinical

For peer review only

(years four to six) students were compared using Chi-squared and Fisher's Exact tests where possible. Statistical significance level was set to 5%.

Results

STUDY POPULATION

An 11% response rate (180 responses) was achieved. Fourth-year students were the largest contributors to the study (25% of respondents, see Figure 1).

STUDENTS' UNDERSTANDING OF CM

There was a wide range of expectations for CM teaching. Three core themes were identified (see Table 2).

Table 2: *Themes and sub-themes identified from qualitative thematic analysis of responses to the free-text question 'What do you understand by the term 'CM'?'.*

Core Theme		Sub-themes	
1	**CM Learning**	1	Students' Perceived Relevance of CM Knowledge and Its Application for their Future Clinical Practice
		2	Students' Perceived Relevance of CM to Healthcare Professionalisms
		3	How to Approach Learning CM
2	**The Relationship Between Food and Health**	1	Diet and Physical and Mental Health
		2	Social Factors
3	**Evidence-based Medicine**		

<u>**Core Theme 1: CM Learning**</u>

Medical students shared their understanding of CM, suggesting where CM teaching might fit their undergraduate medical training.

<u>Sub-theme 1: Students' Perceived Relevance of CM Knowledge and Its Application for their Future Clinical Practice</u>

Students shared their expectations to gain clinical knowledge and practical skills from the CM course.

> *"**applicable to a clinical session**"* (Third Year).

Learning how to facilitate discussions about diet with patients arose as a key topic.

> *"**sensitively debunk health fads**"* (Fifth Year).

> *"**conduct a consultation without body shaming**"* (Fifth Year).

Students stated that they would like to be taught how to recognise different eating habits in order to increase their understanding of patients' diets to better facilitate future planning discussions.

For peer review only

*“**understanding different people's feeding behaviours**”* (Second Year).

*“**diet lifestyles**, such as **veganism**”* (Second Year).

Other students were unfamiliar with the term CM.

“***first time I came across this term***” (Fifth Year).

Students who were in favour of a CM course stressed the importance of the topic and considered how the knowledge and skills that they will learn from the course will make them more competent to educate patients about diet and thus improve patients’ health outcomes.

“*this **valuable knowledge**… is **applicable to every specialty***” (Fifth Year).

“*CM involves using nutrition to **improve patient's health**, including **teaching patients how to eat and cook healthily***” (Fourth Year).

However, some students felt that learning CM would not benefit them greatly. Students expressed worry that other topics in the current MBBS curriculum, which they felt were more useful than CM, might be compromised or completely removed.

*“**other parts of the curriculum… should be better developed and prioritised**”* (Third Year).

<u>Sub-theme 2: Students’ Perceived Relevance of CM to Healthcare Professional Practice</u>

A variety of perspectives on how CM relates to medical specialities arose.

Students associated CM with nutrition and lifestyle medicine.

*“related to **nutrition**”* (Fifth Year).

*“part of the wider discipline of **‘lifestyle medicine’**”* (Third Year).

Students felt that it may be more appropriate for HCPs who have greater clinical expertise in diet and nutrition compared to doctors to counsel patients about diet.

*“**allied health professionals… are better suited in coaching patients in dietary change,** such as **dietitians**”* (Fifth Year).

<u>Sub-theme 3: How to Approach Learning CM</u>

There was a wide variety of opinions on the most appropriate methods for teaching undergraduate medical students CM. Students who were enthusiastic about the idea of learning culinary skills anticipated that practical learning would be engaging and would also allow them to gain knowledge and skills that would enable them to improve their own health and wellbeing.

*“a practical kitchen/cooking session… would add **variety**”* (First Year).

*“practical kitchen sessions would **provide great insight into how to prepare healthy meals for students as well as future doctors**”* (Fourth Year).

However, other students questioned the feasibility of teaching in a kitchen and whether the skills gained would be useful in future clinical practice.

*“a practical "kitchen session" could be very **difficult to organise**”* (Fifth Year).

> *"I **don't really see how it is practical** to teach practical culinary skills [or] **what advantage this would confer**… **we aren't going to be cooking patients' food**"* (Fourth Year).

Some students felt that observing HCPs with expertise in diet and nutrition in clinical practice would be a valuable learning experience.

> *"**shadow a dietitian**"* (Fifth Year).

> *"sitting in with **diabetic nurses** and going through some of the **dietary restrictions** [for] patients"* (Fifth Year).

Other students expressed preference for more traditional teaching methods.

> *"a **formal tutorial** on the… **DAFNE** [Dose Adjustment For Normal Eating] **diet** would [be] even more useful"* (Fifth Year).

Students shared their thoughts on how to integrate CM into the current MBBS curriculum. CM was felt to be closely associated with motivational interviewing, which is covered more broadly elsewhere in the MBBS curriculum outside of the context of diet. Motivational interviewing involves engaging people in exploring their strengths and aspirations regarding a lifestyle habit, evoking motivation to change their behaviour, and supporting them in achieving goals for health and wellbeing improvement.(17)

> *"**how to elicit behaviour change**"* (Second Year).

> *"lifestyle advice [teaching] could be **combined with the patient centred pathway teaching** in year 5 **on motivational interviewing**"* (Fifth Year).

Some students felt that it would be appropriate to dedicate up to one day to learning CM and explained that medical students are likely to already have a basic understanding of what constitutes a healthy diet from teaching received elsewhere in the MBBS curriculum.

> *"CM **day**… **any more than that would be excessive**. **Most students will already know the basics of constructing a good diet**"* (Fifth Year).

There were a range of opinions on which year(s) of the MBBS curriculum the CM course should be delivered in.

> *"integrate **preclinical** science into FNM ['Fluids, Nutrition and Metabolism' - a module taught in year one of the MBBS curriculum], then teach **clinically** in **year 5**"* (Fourth Year).

> *"take this module as a **final year** medical student… as an **optional extra module**"* (Fifth Year).

<u>Core Theme 2: The Relationship Between Food and Health</u>

Students described their views on the links between diet, social factors and health.

<u>Sub-theme 1: Diet and Physical and Mental Health</u>

Students highlighted the role of diet in preventing, treating and managing disease, and alleviating symptoms due to ill-health.

> "*nutrition and lifestyle factors associated with diet to both **prevent disease** and to **improve morbidity and mortality***" (Fifth Year).

Students also recognised mental health problems related to eating.

For peer review only

> *"**eating disorders** including **anorexia**, **bulimia**, **binge eating disorder** and other **behavioural disorders**"* (Fourth Year).

Students emphasised the need for doctors to recognise the relationships that patients have with food and to promote the health benefits of a balanced diet.

> *"**dieting** has been proven to have numerous **negative effects on health**. [CM] teaching should **focus [on] the health benefits of good diet** and should not label foods as **'good'** or **'bad'** as this leads to **moral judgement**, **restriction** and **reactive bingeing** on **'forbidden' foods**"* (Fourth Year).

Sub-theme 2: Social Factors

Students suggested social factors that determine diet and health, including ethnic and cultural background, socio-economic status and level of education.

> "***level of education**... affects your health*" (Fourth Year).

Students stressed the importance of doctors identifying social factors related to diet and nutrition, such as cultural variation in diets, to enable them to provide patient-centred dietary advice.

> "***tailor** [patients' diets] to **lifestyle**, **ethnic**, **economic/ social factors***" (Third Year).

> *"my mum **cooks traditional Indian food on a daily basis**... [my parents] **prefer seeing an Indian GP when they go to the doctors**"* (Third Year).

Core Theme 3: Evidence-Based Medicine

Students expressed mixed opinions about the evidence base for CM.

> *"CM is the **evidence-based practice** of influencing health through lifestyle changes in food consumption"* (First Year).

> *"I'm worried about the **quality** and the **evidence base** for CM. **Diet and nutrition are... affected by... low-quality research**"* (Fifth Year).

INTEGRATING CM KNOWLEDGE INTO CLINICAL PRACTICE

Students' self-perceived confidence and competence related to providing nutritional care were identified (see Table 3).

Table 3: *Results from quantitative analysis of responses to the Likert-type questions. Results are presented as number (percentage).*

Statement		Pre-Clinical (n=81)	Clinical (n=99)	Total (n=180)	p-value
**I am able to facilitate a discussion with patients about diet and/or dietary change.**	Disagree	39 (48.1)	25 (25.3)	64 (35.6)	p = 0.005
	Neutral	28 (34.6)	44 (44.4)	72 (40.0)	
	Agree	14 (17.3)	30 (30.3)	44 (24.4)	
**I am able to take dietary history from a patient.**	Disagree	59 (71.6)	43 (43.4)	101 (56.1)	p = 0.001
	Neutral	14 (17.3)	31 (31.3)	45 (25.0)	
	Agree	9 (11.1)	25 (25.3)	34 (18.9)	
**I am able to apply principles,**	Disagree	52 (64.2)	56 (56.6)	108 (60.0)	p =

For peer review only

**methods and knowledge relating to nutrition to medical practice and integrate these into patient care.**	Neutral	22 (27.2)	30 (30.3)	52 (28.9)	0.498
	Agree	7 (8.6)	13 (13.1)	20 (11.1)	
**I am able to provide dietary advice for patients with varying cultural, social and economic needs.**	Disagree	57 (70.4)	63 (63.6)	120 (66.7)	p = 0.617
	Neutral	18 (22.2)	26 (26.3)	44 (24.4)	
	Agree	6 (7.4)	10 (10.1)	16 (8.9)	
**I believe that learning CM is important for my future clinical practice.**	Disagree	6 (7.4)	4 (4.0)	10 (5.6)	p = 0.482
	Neutral	10 (12.3)	10 (10.1)	20 (11.1)	
	Agree	65 (80.2)	85 (85.9)	150 (83.3)	

PROVISION OF CM TEACHING

62% of students reported having received no prior teaching perceived to be related to CM (see Figure 2).

68% pre-clinical and 77% clinical students were dissatisfied with the quality of existing medical school teaching understood to be relevant to CM. 72% of pre-clinical and 84% of clinical students were dissatisfied with quantity of existing medical school teaching understood to be relevant to CM (see Figure 3).

FEATURES OF CM TEACHING

Students felt that it is most appropriate for a CM course to include teaching on weight management and portion control (157 responses, see Figure 4), closely followed by the types of diet and their evidence base (156 responses). Nutrition psychology was the least popular topic (128 responses).

Students felt that the most appropriate methods of providing CM teaching are problem-based learning (140 responses, see Figure 2) and small-group seminars/ tutorials (138 responses).

67% of students expressed a preference for CM to be taught in pre-clinical training (pre-clinical students 72%, clinical students 64%, p = 0.257).

Discussion

Although 83% of students in this study felt that learning CM is important for their future clinical practice, 67% did not feel confident in their knowledge, nor competent to provide specific dietary advice. These findings are similar to that of an Australian study[5], which found that most pre-clinical medical students felt that it is important for doctors to have an understanding of nutrition in relation to medical conditions, such as coeliac disease, but fewer than half were confident in applying their knowledge to clinical practice.

Some students in this study anticipated that the task of dietary counselling would be delegated to other HCPs, such as dietitians, rather than doctors. The latter views are contradicted by literature that suggest the health benefits for patients when dietary advice is given by doctors, especially in General Practice.[18–20] This supports the delivery of CM teaching in the year five General Practice placement in the MBBS curriculum. The first aim is to enable students to draw upon their experiences of encounters with patients in General Practice and to share these experiences in group discussion about how to approach case-based

For peer review only

problems effectively. The second aim is to allow students to apply the dietary counselling skills taught on the CM course to future interactions with patients, including during their on-going General Practice placements.

The CM course was introduced in September 2019. This medical school now meets the nutrition education recommendation for undergraduate medical students of the European Society for Clinical Nutrition and Metabolism (ESPEN),[21] which recommends the provision of teaching in three domains: basic nutritional science, public health and clinical nutrition.

There may be several reasons why clinical students in this study reported higher levels of self-perceived confidence and competence in their abilities to facilitate discussions about diet and nutrition with patients, compared to pre-clinical students. Firstly, nutrition teaching outside of CM teaching is delivered in first year of the MBBS curriculum only. During the time that this questionnaire study was receiving responses (December 2018 to April 2019), students in their first year would not yet have received this teaching. Therefore, their knowledge in this area may have been limited. Secondly, clinical exposure predominantly takes place during years four to six of the MBBS curriculum. Senior students therefore have increased patient contact and are more likely to observe HCPs discussing diet and health with patients. Students may have also been offered the opportunity to counsel these patients themselves. Such experiences are likely to have increased clinical students' confidence in this area and may have contributed to the differences in self-reported capabilities between these two groups.

This overall low self-perceived confidence and competence reported by questionnaire respondents may be related to their dissatisfaction with the quality and quantity of pre-existing teaching perceived to be relevant to CM in the MBBS curriculum at the time of this study. This may suggest that this cohort of students had particular interest in, and therefore concern for, undergraduate medical education.

The findings from this study suggest that key components of a CM teaching course placed in the clinical part of an undergraduate medical curriculum are: (i) explaining the clinical and professional relevance of drawing upon patient cases from General Practice placements to aid CM learning; and (ii) supporting medical students in developing an understanding of how this knowledge might be applied in clinical practice to maximise opportunities for supporting health promotion and managing ill health. These findings were subsequently used to inform the development of a CM course at a UK medical school, as described below.

Strengths and Limitations

A strength of this study is that the questionnaire was piloted by two undergraduate medical students, who checked the readability. The students also confirmed that the questions would enable medical students' teaching preferences and expectations for CM learning to be gathered. This validation step increased the reliability of the questionnaire.

Moreover, JX and ShP conducted the early stages of thematic analysis independently to allow for a wider breadth of interpretations to be captured. The analysis team (JX, ShP and SP) then synthesised and discussed the interpretations before reaching an agreement.

For peer review only

The questionnaire response rate was low (11%) which limits the generalisability of the findings. The low response rate may be due to the fact that CM is a relatively new term in Medicine and is not commonly used amongst HCPs in the UK and CM is not yet included on most UK medical schools' curricula. The authors anticipated that students with prior understanding and/or interest in CM, were more likely to complete the questionnaire. Therefore, in an attempt to increase the response rate, advertisements for the questionnaire, as well as the PIS, emphasised that all medical students were eligible to take part in the study and that a wide breadth of experiences and perspectives, including no prior understanding of CM, were welcome. Even with this additional encouragement, the response rate remained low. However, theoretical saturation of the qualitative data was judged to have been reached after coding the first 148 responses in an interim analysis, implying that sufficient qualitative data had been gathered for the views of students to be represented in the free-text responses. This strengthened the credibility of the study.

Another limitation is that questionnaire responses were received from one UK medical school only. Factors such as differences between medical school curricula may limit the transferability of the study findings to other medical schools.

Implications

The findings from this study have been used to inform the development of a CM course for undergraduate students at a UK medical school during their year five General Practice placement.

CM Course Content

The course includes a wide range of teaching topics. The aim is for teaching to meet the learning needs of students, which were identified in this study, as well as GMC[(2)] requirements for nutrition education. The course topics are weight management and portion control, nutritional screening tools, taking a dietary history, motivational interviewing skills, addressing the dietary needs of patients with varying cultural and ethnic backgrounds, types of diet and their evidence-base, and practical culinary skills and food preparation.

CM Teaching Methods

The teaching methods that students in this study expressed preferences for have been included in the course to optimise their learning potential. These teaching methods include an online module, a face-to-face tutorial, case-based discussions, motivational interviewing role-play and culinary skills training. The latter has been suggested to be an effective teaching method to improve students' nutrition knowledge and food identification skills.[(8)]

The course creators prepared patient cases for discussion to maximise clinical relevance. Students also prepare their own summaries of relevant patient cases encountered during their General Practice placements, and are supported by course facilitators to think about how they will apply the principles from CM teaching in future consultations.

Integrating CM Teaching into the Clinical Curriculum

For peer review only

CM teaching has been integrated into the clinical curriculum to enable students to have a basic grounding in the knowledge and practical skills of CM, which are highly-relevant to all healthcare professions.

Future Research in This Area

The questionnaire that was created for this study has been adapted into a post-course questionnaire and is being used to gather students' feedback on their CM learning experiences. The feedback data will be analysed in a separate study.

Conducting this cross-sectional questionnaire study at other medical schools will enable faculties to determine whether there is a need to introduce or improve CM teaching at their medical school to better meet their students' needs and expectations for CM learning and/or GMC[2] requirements for nutrition education.

Future qualitative research in this area will allow deeper exploration of students' thoughts regarding CM teaching.

Conclusion

This cross-sectional quantitative study has provided insight into medical students' understanding of and attitudes towards learning CM. The study has highlighted students' perceptions of how they feel CM knowledge and skills might help them to meet the GMC's Outcomes for Graduates and fulfil their future role as doctors. Students reported a lack of confidence in their dietary counselling skills and felt that they could benefit from further integrated and clinically-relevant teaching. Students' preferences for CM teaching have been taken into consideration in the development of a CM course for fifth-year undergraduate students at a UK medical school during their General Practice placements. This medical school is an exemplar of how CM teaching can be introduced into the clinical component of an undergraduate medical curriculum.

Acknowledgements

The authors acknowledge Professor Ann Griffin, Professor Elizabeth Murray, Miss Valeriya Kopanitsa, Dr Deepash Hosadurg, the National Institute for Health Research School for Primary Care Research (NIHR SPCR) and UCL Student Data Analyst, and are thankful to all the study participants.

Contributions

JX: main author and lead for the study design, participant recruitment, data collection and analysis and write-up. SP: chief investigator, co-supervisor and contributed to the study design, data analysis and write-up. ShP: co-supervisor and contributed to the study design, participant recruitment, data analysis and write-up. VV: supervised the statistical analysis and write-up.

Funding

This work was supported by the National Institute for Health Research School for Primary Care Research (NIHR SPCR) grant number 156780. VV receives Seedcorn Funding from the NIHR SPCR.

For peer review only

Competing Interests

None declared.

Patient Consent for Publication

Not required.

Ethics Approval

University College London Ethics (ref.12471/002).

Provenance and Peer Review

This paper was not peer reviewed prior to submission.

Data Sharing Statement

The data generated from this study is not suitable for sharing beyond that contained within the report. Further information can be obtained from the corresponding author.

For peer review only

References

1. La Puma J. What Is Culinary Medicine and What Does It Do? Popul Health Manag. 2016 Feb;19(1):1–3.
2. General Medical Council. Outcomes For Graduates 2018. 2018; Available from: https://www.gmc-uk.org/-/media/documents/dc11326-outcomes-for-graduates-2018_pdf-75040796.pdf
3. Campbell D. Doctors ‘know too little about nutrition and exercise’ [Internet]. The Guardian; 2016 [cited 2019 Feb 17]. Available from: https://www.theguardian.com/society/2016/oct/19/doctors-know-too-little-about-effects-of-nutrition-and-exercise
4. Crowley J, Ball L, Hiddink GJ. Nutrition in medical education: a systematic review. Lancet Planet Heal. 2019 Sep;3(9):e379–89.
5. Perlstein R, McCoombe S, Shaw C, Nowson C. Medical students’ perceptions regarding the importance of nutritional knowledge and their confidence in providing competent nutrition practice. Public Health [Internet]. 2016;140:27–34. Available from: http://www.sciencedirect.com/science/article/pii/S0033350616302207
6. Telegraph Reporters. GP training is outdated and needs to teach young doctors about nutrition, experts claim [Internet]. Telegraph. 2018 [cited 2019 Feb 17]. Available from: https://www.telegraph.co.uk/news/2018/03/25/gp-training-outdated-needs-teach-young-doctors-nutrition-experts
7. Conroy MB, Delichatsios HK, Hafler JP, Rigotti NA. Impact of a preventive medicine and nutrition curriculum for medical students. Am J Prev Med. 2004 Jul;27(1):77–80.
8. Pang B, Memel Z, Diamant C, Clarke E, Chou S, Gregory H. Culinary medicine and community partnership: hands-on culinary skills training to empower medical students to provide patient-centered nutrition education. Med Educ Online. 2019 Dec;24(1):1630238.
9. Ring M, Cheung E, Mahadevan R, Folkens S, Edens N. Cooking Up Health: A Novel Culinary Medicine and Service Learning Elective for Health Professional Students. J Altern Complement Med. 2019 Jan;25(1):61–72.

10. Schlair S, Hanley K, Gillespie C, Disney L, Kalet A, Darby PC, et al. How Medical Students' Behaviors and Attitudes affect the Impact of a Brief Curriculum on Nutrition Counseling. J Nutr Educ Behav [Internet]. 2012;44(6):653–7. Available from: http://www.sciencedirect.com/science/article/pii/S149940461100529X

11. Culinary Medicine UK. Culinary Medicine UK [Internet]. 2020 [cited 2020 May 12]. Available from: https://culinarymedicineuk.org

12. QSR International Pty Ltd. NVivo (Version 12). QSR International Pty Ltd; 2018.

13. Braun V, Clarke V. Using thematic analysis in psychology. Qual Res Psychol [Internet]. 2006 Jan 1;3(2):77–101. Available from: https://www.tandfonline.com/doi/abs/10.1191/1478088706qp063oa

14. Stuckey H. The second step in data analysis: Coding qualitative research data . J Soc Heal Diabetes [Internet]. 2015 Jan 1;3(1):7–10. Available from: http://www.joshd.net/article.asp?issn=2321-0656

15. Hewitt-Taylor J. Use of constant comparative analysis in qualitative research. Nurs Stand. 2001 Jul;15(42):39–42.

16. IBM Corp. IBM SPSS Statistics for Macintosh, Version 25.0. Armonk, NY: IBM Corp; 2017.

17. Miller WR, Rollnick S. Ten Things that Motivational Interviewing Is Not. Behav Cogn Psychother. 2009;37(2):129–140.

18. Willaing I, Ladelund S, Jørgensen T, Simonsen T, Nielsen LM. Nutritional counselling in primary health care: a randomized comparison of an intervention by general practitioner or dietician. Eur J Cardiovasc Prev Rehabil [Internet]. 2004;11(6):513–20. Available from: https://doi.org/10.1097/01.hjr.0000152244.58950.5f

19. Pritchard DA, Hyndman J, Taba F. Nutritional counselling in general practice: a cost effective analysis. J Epidemiol Community Health [Internet]. 1999 May;53(5):311–6. Available from: https://www.ncbi.nlm.nih.gov/pubmed/10396539

20. Sacerdote C, Fiorini L, Rosato R, Audenino M, Valpreda M, Vineis P. Randomized controlled trial: effect of nutritional counselling in general practice. Int J Epidemiol [Internet]. 2005;35(2):409–15. Available from: https://doi.org/10.1093/ije/dyi170

21. Cuerda C, Muscaritoli M, Donini LM, Baqué P, Barazzoni R, Gaudio E, et al. Nutrition education in medical schools (NEMS). An ESPEN position paper. Clin Nutr [Internet]. 2019;38(3):969–74. Available from: http://www.sciencedirect.com/science/article/pii/S0261561419300500

Figure 1: *Results from quantitative analysis of responses to the single choice question 'Which year of the medical (MBBS) programme are you currently in?'.*

Figure 2: *A comparison of the results from quantitative analysis of responses to two multiple choice questions: 'What types of CM training have you received so far in the MBBS curriculum?' and 'Select the 3 most appropriate teaching methods for a CM course'.*

Figure 3: *Results from quantitative analysis of responses to the Likert-type questions: 'To what extent do you agree with the statements: "I am satisfied with the QUALITY of CM training that my medical school provides"? and 'To what extent do you agree with the*

For peer review only

statement "I am satisfied with the QUANTITIY of CM training that my medical school provides"?'.

Figure 4: *Results from quantitative analysis of responses to the multiple choice question 'Which topics and/or skills do you think should be taught on the CM course?'.*

For peer review only

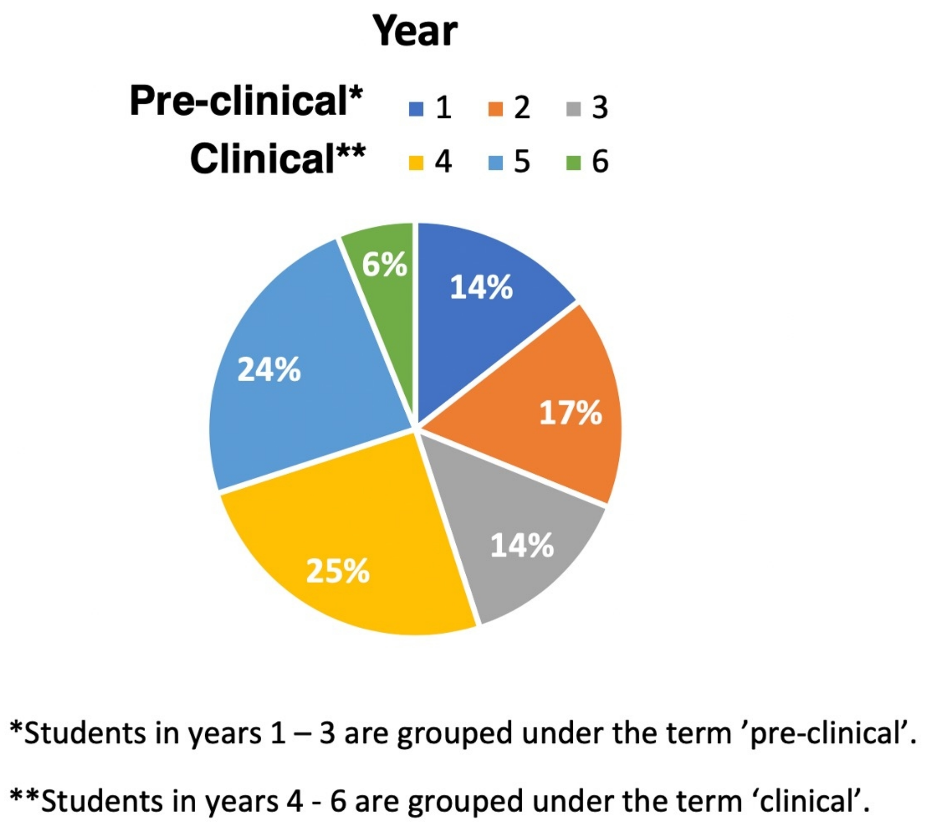

Figure 1: Results from quantitative analysis of responses to the single choice question ‘Which year of the medical (MBBS) programme are you currently in?'.

210x185mm (144 x 144 DPI)

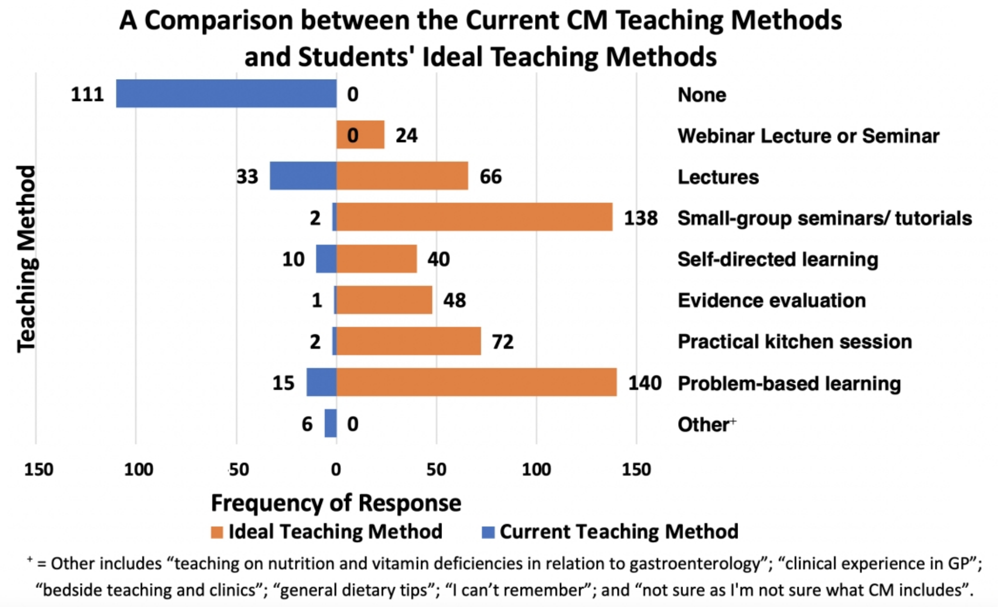

Figure 2: A comparison of the results from quantitative analysis of responses to two multiple choice questions: 'What types of CM training have you received so far in the MBBS curriculum?' and 'Select the 3 most appropriate teaching methods for a CM course'.

412x250mm (144 x 144 DPI)

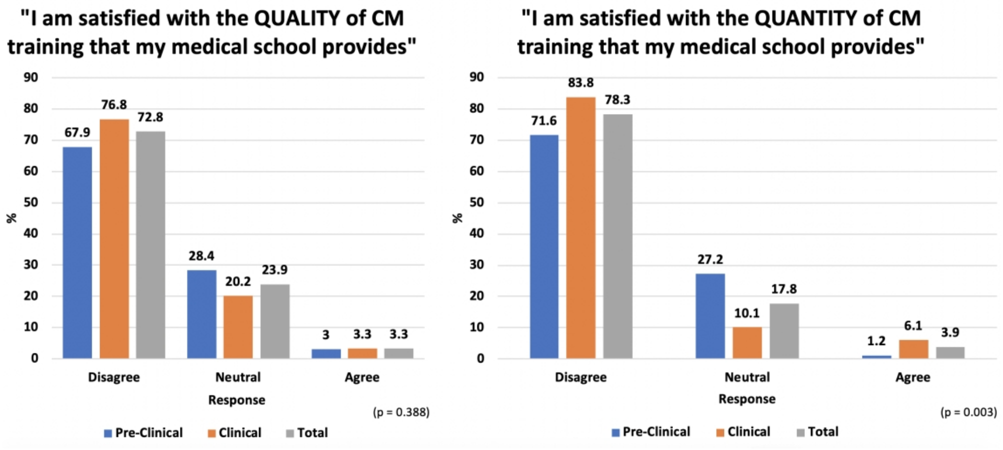

Figure 3: Results from quantitative analysis of responses to the Likert-type questions: ‘To what extent do you agree with the statements: “I am satisfied with the QUALITY of CM training that my medical school provides”? and ‘To what extent do you agree with the statement “I am satisfied with the QUANTITIY of CM training that my medical school provides”?’.

496x223mm (144 x 144 DPI)

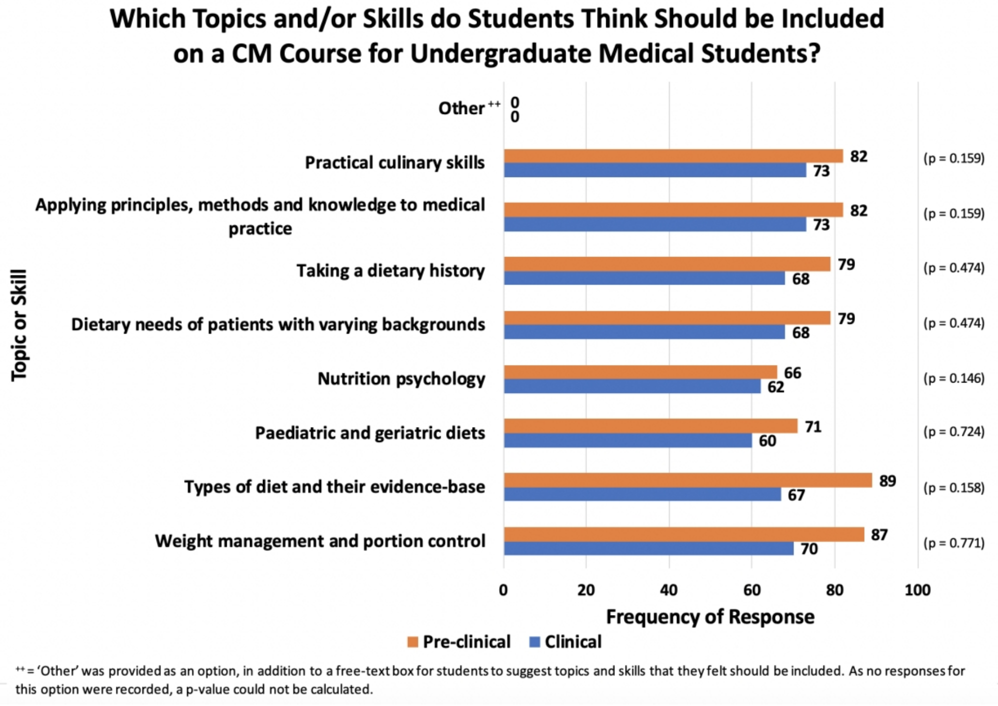

Figure 4: Results from quantitative analysis of responses to the multiple choice question 'Which topics and/or skills do you think should be taught on the CM course?'.

363x257mm (144 x 144 DPI)

Standards for Reporting Qualitative Research (SRQR)*

http://www.equator-network.org/reporting-guidelines/srqr/

Page/line no(s).

Title and abstract

**Title** - Concise description of the nature and topic of the study Identifying the study as qualitative or indicating the approach (e.g., ethnography, grounded theory) or data collection methods (e.g., interview, focus group) is recommended	Page 1, lines 1 - 5
**Abstract** - Summary of key elements of the study using the abstract format of the intended publication; typically includes background, purpose, methods, results, and conclusions	Page 2, lines 1 - 28

Introduction

**Problem formulation** - Description and significance of the problem/phenomenon studied; review of relevant theory and empirical work; problem statement	Page 3, lines 14 – 24, 36 – 40
**Purpose or research question** - Purpose of the study and specific objectives or questions	Page 3, line 40<br>Page 4, lines 1-2

Methods

**Qualitative approach and research paradigm** - Qualitative approach (e.g., ethnography, grounded theory, case study, phenomenology, narrative research) and guiding theory if appropriate; identifying the research paradigm (e.g., postpositivist, constructivist/ interpretivist) is also recommended; rationale**	Page 4, lines 4 – 8, 20 - 23
**Researcher characteristics and reflexivity** - Researchers' characteristics that may influence the research, including personal attributes, qualifications/experience, relationship with participants, assumptions, and/or presuppositions; potential or actual interaction between researchers' characteristics and the research questions, approach, methods, results, and/or transferability	Page 4, lines 5 – 8
**Context** - Setting/site and salient contextual factors; rationale**	Page 4, lines 9 - 10
**Sampling strategy** - How and why research participants, documents, or events were selected; criteria for deciding when no further sampling was necessary (e.g., sampling saturation); rationale**	Page 4, lines 11 – 12
**Ethical issues pertaining to human subjects** - Documentation of approval by an appropriate ethics review board and participant consent, or explanation for lack thereof; other confidentiality and data security issues	Page 5, lines 13 - 21
**Data collection methods** - Types of data collected; details of data collection procedures including (as appropriate) start and stop dates of data collection and analysis, iterative process, triangulation of sources/methods, and modification of procedures in response to evolving study findings; rationale**	Page 4, lines 4 – 5, 13 – 18, table 1<br>Page 5, lines 10 - 11

For peer review only - http://bmjopen.bmj.com/site/about/guidelines.xhtml

**Data collection instruments and technologies** - Description of instruments (e.g., interview guides, questionnaires) and devices (e.g., audio recorders) used for data collection; if/how the instrument(s) changed over the course of the study	Page 4, lines 4 – 5, 13 – 18 , table 1
**Units of study** - Number and relevant characteristics of participants, documents, or events included in the study; level of participation (could be reported in results)	Page 4, lines 9 – 12<br><br>Page 5, lines 4 - 8
**Data processing** - Methods for processing data prior to and during analysis, including transcription, data entry, data management and security, verification of data integrity, data coding, and anonymization/de-identification of excerpts	Page 5, lines 24 - 34
**Data analysis** - Process by which inferences, themes, etc., were identified and developed, including the researchers involved in data analysis; usually references a specific paradigm or approach; rationale**	Page 5, lines 24 – 39<br>Page 6 lines 1 - 2
**Techniques to enhance trustworthiness** - Techniques to enhance trustworthiness and credibility of data analysis (e.g., member checking, audit trail, triangulation); rationale**	Page 5, lines 3 – 8<br>Page 11 lines 35 - 37

Results/findings

**Synthesis and interpretation** - Main findings (e.g., interpretations, inferences, and themes); might include development of a theory or model, or integration with prior research or theory	Page 6, line 3- page 10, line 17
**Links to empirical data** - Evidence (e.g., quotes, field notes, text excerpts, photographs) to substantiate analytic findings	Page 6, line 3- page 10, line 17

Discussion

**Integration with prior work, implications, transferability, and contribution(s) to the field -** Short summary of main findings; explanation of how findings and conclusions connect to, support, elaborate on, or challenge conclusions of earlier scholarship; discussion of scope of application/generalizability; identification of unique contribution(s) to scholarship in a discipline or field	Page 10, line 18 - page 11 line 29
**Limitations** - Trustworthiness and limitations of findings	Page 11, lines 30 – 41<br>Page 12 lines 1 - 12

Other

**Conflicts of interest** - Potential sources of influence or perceived influence on study conduct and conclusions; how these were managed	Page 13, line 37
**Funding** - Sources of funding and other support; role of funders in data collection, interpretation, and reporting	Page 13, lines 33 - 35

For peer review only - http://bmjopen.bmj.com/site/about/guidelines.xhtml

*The authors created the SRQR by searching the literature to identify guidelines, reporting standards, and critical appraisal criteria for qualitative research; reviewing the reference lists of retrieved sources; and contacting experts to gain feedback. The SRQR aims to improve the transparency of all aspects of qualitative research by providing clear standards for reporting qualitative research.

**The rationale should briefly discuss the justification for choosing that theory, approach, method, or technique rather than other options available, the assumptions and limitations implicit in those choices, and how those choices influence study conclusions and transferability. As appropriate, the rationale for several items might be discussed together.

Reference:

O'Brien BC, Harris IB, Beckman TJ, Reed DA, Cook DA. **Standards for reporting qualitative research: a synthesis of recommendations.** *Academic Medicine*, Vol. 89, No. 9 / Sept 2014 DOI: 10.1097/ACM.0000000000000388

For peer review only
